# 2′-*O*-(*N*-(Aminoethyl)carbamoyl)methyl Modification Allows for Lower Phosphorothioate Content in Splice-Switching Oligonucleotides with Retained Activity

**DOI:** 10.1089/nat.2021.0086

**Published:** 2022-06-01

**Authors:** Dmytro Honcharenko, Cristina S.J. Rocha, Karin E. Lundin, Jyotirmoy Maity, Stefan Milton, Ulf Tedebark, Merita Murtola, Malgorzata Honcharenko, Andis Slaitas, C.I. Edvard Smith, Rula Zain, Roger Strömberg

**Affiliations:** ^1^ Department of Biosciences and Nutrition, Karolinska Institutet, Huddinge, Sweden.; ^2^ Department of Laboratory Medicine, Clinical Research Center, Karolinska Institutet, Karolinska University Hospital, Huddinge, Sweden.; ^3^ Syntagon Baltic, Babites novads, Latvia.; ^4^ Department of Clinical Genetics, Center for Rare Diseases, Karolinska University Hospital, Stockholm, Sweden.

**Keywords:** *2′-*O*-(*N*-(aminoethyl)carbamoyl)methyl modification*, splice-switching oligonucleotides, cellular uptake, cell-penetrating ONs, phosphorothioate linkage, oligonucleotide–peptide conjugate

## Abstract

2′-*O*-(*N*-(Aminoethyl)carbamoyl)methyl (2′-*O*-AECM)-modified oligonucleotides (ONs) and their mixmers with 2′-*O*-methyl oligonucleotides (2′-OMe ONs) with phosphodiester linkers as well as with partial and full phosphorothioate (PS) inclusion were synthesized and functionally evaluated as splice-switching oligonucleotides in several different reporter cell lines originating from different tissues. This was enabled by first preparing the AECM-modified A, C, G and U, which required a different strategy for each building block. The AECM modification has previously been shown to provide high resistance to enzymatic degradation, even without PS linkages. It is therefore particularly interesting and unprecedented that the 2′-*O*-AECM ONs are shown to have efficient splice-switching activity even without inclusion of PS linkages and found to be as effective as 2′-OMe PS ONs. Importantly, the PS linkages can be partially included, without any significant reduction in splice-switching efficacy. This suggests that AECM modification has the potential to be used in balancing the PS content of ONs. Furthermore, conjugation of 2′-*O*-AECM ONs to an endosomal escape peptide significantly increased splice-switching suggesting that this effect could possibly be due to an increase in uptake of ON to the site of action.

## Introduction

Therapeutic oligonucleotides (ONs) provide an opportunity for treating severe diseases where options are limited using traditional low-molecular-weight molecules and antibody drugs [1]. The bulk of nucleic acid medicines in clinical trials are inhibitory antisense ONs (AONs) directed against messenger RNA (mRNA). Today, the field encompasses therapeutic ONs with a number of different modes of action, including effects on microRNAs, splice-switching, aptamers, and mRNA therapy [2–5]. Although there are many ongoing clinical trials and some approved drugs [5–8], ON therapeutics are only lately starting to meet the early expectations. Several recent FDA approvals predict a new dawn for this class of drugs [6–11]. It is, however, recognized that the most severe limitations in ON therapeutics are the *in vivo* bioavailability and delivery (or rather lack thereof) to the target site [5,12,13].

Numerous chemical modifications of ONs have been developed to improve their properties, such as nuclease stability, target affinity, and specificity [5,14,15]. In particular, 2′-*O*-alkyl-modified ONs are of great interest, displaying enhanced affinity to RNA, and have been successfully employed as splice-switching ONs (SSOs), in antisense gapmers for mRNA depletion or in small interfering RNA (siRNA) applications [16–18]. 2′-*O*-Alkyl [eg, 2′-*O*-methyl (2′-OMe), 2′-*O*-methoxyethyl, cEt] ONs combined with phosphorothioate (PS) modifications [19,20] are among the most exploited ONs in clinical trials [5,8,14].

To provide more efficient delivery of ONs, various methods, including covalent attachment of small molecules, cationic lipids, peptides, and polymers, or the use of natural and synthetic nanocarriers, have been developed [21–26]. Other proposed solutions to enhance cell membrane penetration by ONs include masking negative charges using a prodrug-like approach [27–29] as well as introduction of positive charges to reduce their overall negative charge [30,31].

We have shown an ON containing 2′-*O*-(*N*-(aminoethyl)carbamoyl)methyl (2′-*O*-AECM) modifications [32], which is taken up by cells without any additives, such as cationic lipids or cell-penetrating peptides (CPPs). Initial studies using dinucleotides showed that the 2′-*O*-AECM modification also confers high stability to 3′- and 5′-exonucleases [33].

We also have shown that an oligo-A sequence fully modified with 2′-*O*-AECM but with phosphodiester linkages was resistant to degradation in human serum [32]. These properties make 2′-*O*-AECM ONs highly interesting candidates for further development into ON therapeutics. Thus, we describe, in this study, the synthesis of 2′-*O*-AECM-modified ribonucleosides, and evaluate the properties of ONs containing 2′-*O*-AECM units and their potential for splice-switching. The cellular uptake is studied by confocal microscopy, and by the efficiency of 2′-*O*-AECM ONs in splice-switching assays in reporter cell lines derived from different tissues.

We also study the influence of the number of 2′-*O*-AECM modifications on cellular uptake, and evaluate the effect of modulation of PS content on the splice-switching efficiency of 2′-*O*-AECM containing ONs. The ability to balance the PS content is especially important with regard to known PS ON toxicity [34–40]. Recent studies show that the replacement of even one PS linkage at a specific site of DNA-gap can reduce toxicity of hepatoxic gapmer AONs [41]. In addition, a conjugate of a 2′-*O*-AECM-modified ON with an endosomal escape promoting peptide was also evaluated. The new chemistry was further evaluated versus a gene target using a mutated *BTK* intron 4 where splice correction was assessed by quantitative polymerase chain reaction.

## Materials and Methods

General information and methods as well as experimental details on synthesis of the new 2′-*O*-(*N*-(aminoethyl)carbamoyl)methyl modified building blocks are presented in the Supplementary Data pp. S2–S10.

### Synthesis of 2′-O-AECM-modified ONs

**ONs1-10**, **ONs12-15**, **ON17**, **ON19**, and **ON22** (for sequences see Table 1) were assembled in TWIST^™^ synthesis columns (Glen Research) on an Applied Biosystems 392A DNA/RNA synthesizer using monomers **5**, **11**, **17**, and **2′-*O*-AECM-A** [32,33] (dried *in vacuo* in the presence of P_2_O_5_ before use) and/or commercial phosphoramidites 2′-OMe-A^Bz^, 2′-OMe-G^iBu^, 2′-OMe-U, and 2′-OMe-C^Ac^ (Glen Research) typically with overall yields from 50% to 80% (based on detritylation data). Glen UnySupport-controlled pore glass (CPG) 500 (Glen Research) was used as a solid support for **ON1** and **ONs12-15**, Glen UnySupport FC CPG 1000 (Glen Research) was used for **ON22**, 3′-(6-fluorescein) CPG (Glen Research) was used for **ONs2-10**, and 2′-OMe-A-RNA-CPG for **ON17** and **ON19**.

**Table 1.** Oligonucleotides Used in the Study

ON	Sequence 5′ → 3′^a^	ESI-TOF mass m/z^b^		T_m_ (°C)^c^	ΔT_m_/Mod (°C)^d^
		Calculated	Found		
**ON1**	**CCUCUUACCUCAGUUACA**	1,474.09^e^	1,473.89^e^	69.0	+0.4
**ON2**	**CCUCUUACCUCAGUUACA**-6Fam	1,587.59^e^	1,587.68^e^		
**ON3**	**CCUCUUACCUCAGUUA**-6Fam	1,420.66^e^	1,420.47^e^		
**ON4**	**CCUCUUACCUCA**-6Fam	1,354.28^f^	1,354.45^f^		
**ON5**	**CCUCUU**ACCUCA-6Fam	1,225.14^f^	1,225.36^f^		
**ON6**	**CC**UCUUACCUCA-6Fam	911.04^e^	910.96^e^		
**ON7**	C^*^C^*^U^*^C^*^U^*^U^*^ACCUCAGUUACA-6Fam	1,503.56^e^	1,503.58^e^		
**ON8**	C^*^C^*^U^*^C^*^U^*^U^*^A^*^C^*^C^*^U^*^C^*^A^*^G^*^U^*^U^*^A^*^C^*^A^*^-6Fam	1,542.11^e^	1,542.19^e^		
**ON9**	C^*^C^*^U^*^C^*^U^*^U^*^A^*^C^*^C^*^U^*^C^*^A^*^G^*^U^*^U^*^A^*^C^*^A^*^-6Fam	1,645.43^e^	1,645.19^e^		
**ON10**	C^*^C^*^U^*^C^*^U^*^U^*^A^*^C^*^C^*^U^*^C^*^A^*^G^*^U^*^U^*^A^*^C^*^A^*^-6Fam	1,335.49^e^	1,335.75^e^		
**ON11**	CCUCUUACCUCAGUUACA	^g^	^g^	76.0	+0.8
**ON12**	**CCUCUU**ACCUCA**GUUACA**	1,370.78^e^	1,370.71^e^	70.0	+0.5
**ON13**	C^*^C^*^U^*^C^*^U^*^U^*^A^*^C^*^C^*^U^*^C^*^A^*^G^*^U^*^U^*^A^*^C^*^A	1,425.41^e^	1,425.33^e^	63.0	+0.1
**ON14**	**CCUCUU**A^*^C^*^C^*^U^*^C^*^A^*^GUUACA	1,390.06^e^	1,390.02^e^	67.0	+0.3
**ON15**	C^*^C^*^U^*^C^*^U^*^U^*^A ^*^ **C** ^*^ **C** ^*^ **U** ^*^ **C** ^*^ **A** ^*^ G^*^U^*^U^*^A^*^C^*^A	1,528.72^e^	1,528.82^e^	59.0	−0.1
**ON16**	C^*^C^*^U^*^C^*^U^*^U^*^A^*^C^*^C^*^U^*^C^*^A^*^G^*^U^*^U^*^A^*^C^*^A	^g^	^g^	72.0	+0.6
**ON17**	**CCUCUUACCUCAGUUAC**A	1,821.34^f^	1,821.48^f^		
**ON18** ^h^	ELL-peptide-**CCUCUUACCUCAGUUAC**A	2,345.14^f^	2,345.45^f^		
**ON19**	**CAGAGUUCUCAGGAUGU**A	1,867.13^f^	1,867.05^f^		
**ON20** ^h^	ELL-peptide-**CAGAGUUCUCAGGAUGU**A	2,394.44^f^	2,394.45^f^		
**ON21**	C^*^A^*^G^*^A^*^G^*^U^*^U^*^C^*^U^*^C^*^A^*^G^*^G^*^A^*^U^*^G^*^U^*^A	^g^	^g^		
**ON22**	**CCGCUGGUCCUCAGGAA**C	1,854.62^f^	1,854.67^f^		
**ON23** ^h^	ELL-peptide-**CCGCUGGUCCUCAGGAA**C	1,905.34^e^	1,905.39^e^		
**ON24**	C^*^C^*^G^*^C^*^U^*^G^*^G^*^U^*^C^*^C^*^U^*^C^*^A^*^G^*^G^*^A^*^A^*^C	^g^	^g^		

^a^ In all sequences **A** = 2′-*O*-AECM-adenosine, **G** = 2′-*O*-AECM-guanosine, **C** = 2′-*O*-AECM-cytidine, **U** = 2′-*O*-AECM-uridine, A = 2′-OMe-adenosine, G = 2′-OMe-guanosine, C = 2′-OMe-cytidine, U = 2′-OMe-uridine, 6Fam = 6-fluorescein, ^*^ = PS linkages.

^b^ Negative mode.

^c^ Duplex thermal stability (*T*_m_ °C) with target RNA; *T*_m_ values measured as the maximum of the first derivative (absorbance vs. temperature) of the UV melting curves at 260 nm with 4 μM strand concentration in 10 mM phosphate buffer, 100 mM NaCl, 0.1 mM EDTA, pH 7.0.

^d^ Δ*T*_m_/mod is the difference in *T*_m_ relative to the unmodified DNA [5′-d(CCTCTTACCTCAGTTACA)-3′] duplex with complementary RNA per modification (*T*_m_ = 61.5°C).

^e^ [M]^−5^.

^f^ [M]^−4^.

^g^ Purchased ONs.

^h^ The sequence (N → C) of the ELL-peptide is WGELLEALAELLEG, see Supplementary Scheme S1.

2′-*O*-AECM, 2′-*O*-(*N*-(aminoethyl)carbamoyl)methyl; 2′-OMe, 2′-*O*-methyl; EDTA, ethylenediamine tetraacetate; ON, oligonucleotide; PS, phosphorothioate; UV, ultraviolet.

Significance of bold in column 1 refers to compound identifiers.

ONs were synthesized using a standard RNA synthesis procedure on a 1.0 μmol scale with 0.3 M 5-benzylthio-1-*H*-tetrazole (BTT) as activator and 600 s coupling time. For the preparation of ONs with PS linkages (**ONs7-10**, and **ONs13-15**), the sulfurizing reagent PADS [42] (0.2 M phenylacetyl disulfide in CH_3_CN/3-methylpyridine) was used (120 s) after the coupling step. Detritylation was accomplished with 3% (w/v) trichloroacetic acid (TCA) in dichloromethane (DCM; 145 s). Deprotection of ONs and their release from the solid support were carried out with an anhydrous solution of ethylenediamine–methanol (1:4 v/v) for 24 h at room temperature. The CPG was removed by filtration and washed with methanol (1 mL) and water (3 × 1 mL).

The combined filtrate was evaporated under reduced pressure at 30°C, water was added to the residue, and the crude product was lyophilized. The crude **ONs1-6**, **ON12**, **ON17**, **ON19**, and **ON22** were purified by reversed-phase (RP) HPLC, whereas crude PS **ONs7-10** and **ONs13-15** were first purified by ion-exchange (IE) HPLC and then desalted using RP-HPLC (Supplementary Data). All ON sequences and their characteristics are shown in Table 1. Concentrations of ONs were determined by ultraviolet (UV) absorption at 260 nm and calculated from extinction coefficients obtained by the nearest neighbor approximation [43].

### Synthesis of 2′-O-AECM-modified ON–ELL-peptide conjugates (ON18, ON20, and ON23)

The CPG-supported **ON17**, **ON19**, or **ON22** (0.75 μmol) was each placed in 1 μmol TWIST synthesis column, washed with anhydrous pyridine (2 mL), and flashed with nitrogen gas. The 4-methoxytrityl (MMTr)-protected aminolinker H-phosphonate [44] (16.8 mg, 31 μmol) was dissolved in anhydrous pyridine (0.4 mL, 62 mM coupling solution) and added to the solid-supported ON. Immediately pivaloyl chloride (4.6 μL, 37 μmol) dissolved in anhydrous acetonitrile was added (final proportion of pyridine–acetonitrile is 4:1 v/v). The reaction mixture was slowly agitated for 25 min at ambient temperature. After that time, the coupling solution was removed and the support was washed with pyridine. Next, the solid-supported ON was treated with 2% of I_2_ in pyridine–water (98:2 v/v) for 15 min. The oxidizing solution was removed and support was then washed extensively with pyridine, CH_3_CN, and DCM. The 5′-amino-protecting group (MMTr) was removed by treatment with 3% (w/v) TCA in DCM (continuous flow of 7 mL for 5 min) and support was washed with DCM and CH_3_CN.

In an Eppendorf tube, 4-((2-(prop-2-yn-1-yloxy)acetamido)methyl) benzoic acid (PAMBA) [45] (24.5 mg, 0.1 mmol) was dissolved in anhydrous *N*,*N*-dimethylformamide (DMF; 0.5 mL). To the resulting solution HBTU (37.9 mg, 0.1 mmol) was added followed by the addition of NMM (110 μL, 1 mmol) and the reaction mixture was agitated on a vortex for 30 min at ambient temperature. The preactivation solution was then transferred to the CPG-supported ON in the TWIST synthesis column, which was then gently agitated on a vortex for 2 h at ambient temperature.

The reaction solution was removed and the solid-supported ON was extensively washed with DMF, CH_3_CN, and DCM and dried by a 1 min flush with nitrogen gas. The resulting CPG-supported 5′-alkyne-modified ON (0.25 μmol) was placed in a separate 2-mL Eppendorf tube with a screw cap and the azido functionalized peptide (ELL-peptide; 1.71 mg, 1 μmol) dissolved in 195 μL of *tert*-butanol/H_2_O (1:1 v/v) was added.

To the resulting mixture, the *N*,*N*-diisopropylethylamine (DIPEA) solution [1.25 μmol, 5 μL from a stock solution of 1 mL of *tert*-butanol/H_2_O (1:1 v/v) containing 43 μL of DIPEA] was added followed by the addition of CuI [0.5 mmol, 50 mL from a stock solution of 1.9mg of CuI in 1mL of dimethyl sulfoxide (DMSO)]. The reaction mixture was gently agitated on a vortex for 24 h at ambient temperature.

After that time the mixture was centrifuged and the solution above the support was carefully removed using a syringe with a needle. CPG-supported ON–peptide conjugate was then washed sequentially with *tert*-butanol/H_2_O (1:1 v/v, 2 × 0.5 mL), 1 mM solution of ethylenediamine tetraacetate (EDTA) in *tert*-butanol/H_2_O (1:1 v/v, 2 × 0.5 mL), *tert*-butanol/H_2_O (1:1 v/v, 3 × 0.5 mL), and CH_3_CN (2 × 0.5 mL), and dried by a 1 min flush with nitrogen gas. The resulting solid-supported product was treated with an anhydrous mixture of ethylenediamine–methanol (1:4 v/v) for 24 h at room temperature.

The CPG was removed by filtration using Millex syringe-driven filter (0.22 μm) into 50-mL round-bottomed flask and the residue in the Eppendorf tube and filter were washed with methanol (1 mL) and Milli-Q water (3 × 1 mL). The combined filtrate was concentrated under reduced pressure at 30°C, and the residue was redissolved in water, transferred to Eppendorf tube, and lyophilized.

The crude deprotected **ON18**, **ON20**, or **ON23** were purified [an aqueous solution containing EDTA (0.25 μmol) was added to the solution of crude ON before purification] using RP HPLC on C18 column (Supelco Discovery^®^ BIO Wide Pore C18-5) with a linear gradient from 50% to 100% of buffer B in buffer A over 45 min at 25°C, *t*_R_ = 32.8 min (**ON18**), *t*_R_ = 34.4 min (**ON20**), and *t*_R_ = 36.0 min (**ON23**). Buffer (A): 50 mM triethylammonium acetate (TEAA), pH 6.5; buffer (B): 50 mM TEAA, pH 6.5 in H_2_O-CH_3_CN (1:1 v/v). No particular solubility problems were observed for the ON peptide conjugates and 40-50 μM aqueous stock solutions were prepared. Concentrations of ONs were determined by UV absorption at 260 nm and calculated from extinction coefficients obtained by the nearest neighbor approximation [43].

### ON cellular uptake visualization by confocal laser scanning microscopy

Human osteosarcoma U-2 OS cells were maintained and cultivated in Dulbecco's modified Eagle's medium (DMEM), high glucose plus 10% fetal bovine serum (FBS) at 37°C, 5% CO_2_, and 95% humidity. Cells were seeded at a density of 2 × 10^4^ cells per well in a 175 μm glass bottom 96-well plate (Greiner Bio-One) until they reached 60% confluence. The fluorescein-labeled ONs (**ON2-10**) were diluted to 1 or 4 μM in OptiMEM (Life Technologies) at 37°C and added to cells after medium removal.

After 8 or 24 h of incubation, the cells were processed for the microscopy as follows: the medium was removed, cells washed once with Opti-MEM at 37°C, and stained with a 6.25 μg/mL solution of CellMask^™^ Deep Red membrane stain (Life Technologies) in Opti-MEM for 10 min at 37°C. After staining, cells were fixed with 4% paraformaldehyde, pH 7.4, in OptiMEM for 5 min at 37°C, followed by three times washing with phosphate-buffered saline (PBS).

The cells were left in PBS and imaged immediately. Confocal laser scanning microscopy was performed using an Inverted Nikon A1R+ Confocal Microscope (Nikon Corporation) with Apo 60 × oil λS DIC N2 objective (NA 1.4, refractive index 1.515) and galvano scanner. Pictures were acquired with the NIS-Elements Advanced Research Software (Nikon Corporation) using a pinhole size of 39.6 μm, and Ti ZDrive performed the Z stack bottom-to-top with ∼0.2 μm/step.

### Cell lines and culture conditions' treatment of different reporter cell lines

Reporter cell lines (HeLa Luc/705, HuH7_705, C2C12_705, Neuro-2a_705, and U-2 OS_705) [46] were maintained in DMEM, high glucose plus 10% FBS and for HuH7_705, C2C12_705, and Neuro-2a_705, 400 μg/mL Geneticin (Life Technologies) at 37°C, 5% CO_2_ in 95% humidity. The stably transfected U-2 OS cell line containing the *EGFPLuc* reporter gene with a mutated *BTK* intron 4 (U-2 OS-mBTKi4) [47] was cultured in the same medium without antibiotics.

For the Luciferase assay, cells were seeded in medium without antibiotic at a density of 8 × 10^3^ (U-2 OS_705; C2C12_705; HuH7_705; and Neuro-2a_705) and 1 × 10^4^ (HeLa Luc/705) cells per well in 96-well plates the day before treatment. The next day, the medium was removed and 100 μL fresh DMEM supplemented with 10% FBS and 9 mM of CaCl_2_ with or without the respective ON was added. After 48 h, another 50 μL of fresh normal medium was added/well and the cultures were incubated for additional 24 h. For the reverse transcription polymerase chain reaction (RT-PCR) experiments, 50,000 cells/well were seeded in 24-well plates. The next day the medium was removed and replaced with 500 μL DMEM/10% fetal calf serum/9 mM CaCl_2_ and ONs as described. Cells were harvested for both Luciferase measurements and for RNA extraction 72 h after the initial addition of the SSOs.

### Luciferase assay

To measure luciferase activity, the medium was removed, wells washed twice with 1 × PBS and cells lysed in 25 μL of 1 × PBS with 0.1% Triton X-100 per well. The plates were incubated for 20 min at 4°C, followed by a frost at −80°C/defrost cycle. Twenty microliters of the lysates were mixed by injector with 100 μL of the Luciferase Assay Reagent [48] (1 mM EDTA pH 8.0, 20 mM Tricine pH 7.8, 1 mM MgCO_3_ pH 7.8, 5 mM MgSO_4_, 25 mM DTT, 1 mM ATP, 25 μM Coenzyme A, and 1 mM d-Luciferin). The relative light units of luciferase were determined using the GloMax^®^ 96 Microplate Luminometer (Promega) with an integration time of 10 s. The values were normalized by the total protein quantity, determined using the *DC* Protein Assay (Bio-Rad) according to the manufacturer's protocol.

## Results and Discussion

**FIG. 1.**
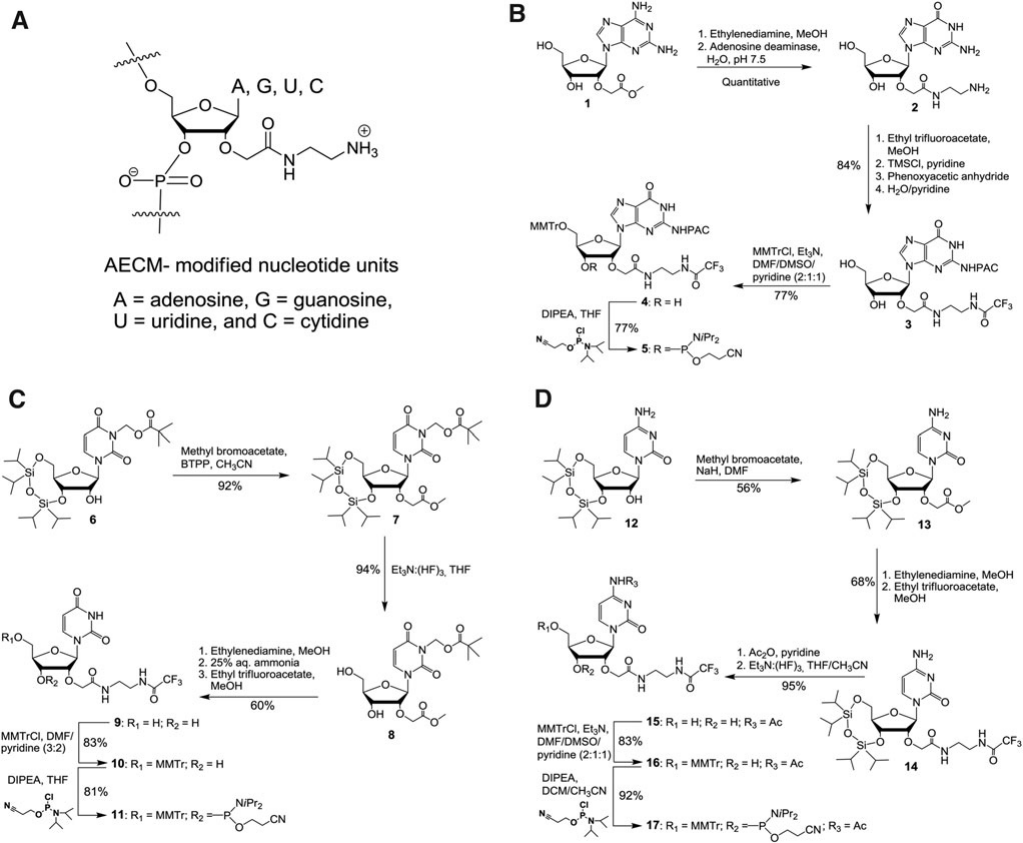
**(A)** The 2′-*O*-(*N*-(aminoethyl)carbamoyl)methyl-modified adenosine (2′-*O*-AECM-A), guanosine (2′-*O*-AECM-G), uridine (2′-*O*-AECM-U), and cytidine (2′-*O*-AECM-C) moieties. **(B)** Synthesis of the 2′-*O*-AECM-G phosphoramidite. **(C)** Synthesis of the 2′-*O*-AECM-U phosphoramidite. **(D)** Synthesis of the 2′-*O*-AECM-C phosphoramidite. Ac, acetyl; BTPP, (*tert*-butylimino)tris(pyrrolidino)phosphorane; DCM, dichloromethane; DIPEA, *N*,*N*-diisopropylethylamine; DMF, *N*,*N*-dimethylformamide; DMSO, dimethyl sulfoxide; *i*Pr, isopropyl; MMTr, 4-methoxytrityl; PAC, phenoxyacetyl; THF, tetrahydrofuran; TMS, trimethylsilyl.

To investigate the properties of 2′-*O*-AECM-modified ONs, we first needed to enable synthesis of ONs with mixed base sequences. Thus, we prepared building blocks from the four most common ribonucleosides (Fig. 1A) and synthesized a number of 2′-*O*-AECM ONs with partial and full PS content and ONs, where the 2′-*O*-AECM is partially replaced with 2′-OMe nucleosides (Table 1). For confocal microscopy studies, all ONs were 3′-end labeled with fluorescein.

### Synthesis of 2′-O-AECM-modified building blocks

Synthesis of the 2′-*O*-AECM adenosine monomer was performed as previously reported [32,33]. For the preparation of the other 2′-*O*-AECM-modified pyrimidine and purine nucleotide units, different synthesis methodologies were developed. The synthesis of the 2′-*O*-(*N*-(aminoethyl)carbamoyl)methylguanosine (2′-*O*-AECM-G) nucleoside and its phosphoramidite was achieved as described in Fig. 1B.

Unprotected 2,6-diaminopurine riboside was regioselectively alkylated at the 2′-position with methyl 2-bromoacetate to obtain compound **1** [49]. This intermediate was first treated with excess of ethylenediamine in methanol and then converted to guanosine derivative **2** by further treatment with adenosine deaminase [50–52]. The primary amino function of the aminoethyl moiety in **2** was protected with a trifluoroacetyl-protecting group using ethyl trifluoroacetate in methanol. Protection of the exocyclic amino group with a phenoxyacetyl (PAC) group, by treatment with phenoxyacetic anhydride under transient protection conditions [53], gave **3** in 84% yield.

Compound **3** was then converted to 5′-*O*-4-methoxytrityl derivative **4** by treatment with 4-methoxytrityl chloride (MMTrCl) in a DMF–DMSO–pyridine (2:1:1 v/v/v) mixture in the presence of triethylamine in 77% yield. Phosphitylation of compound **4** at the 3′-position with 2-cyanoethyl *N*,*N*-diisopropylphosphoramidochloridite in the presence of DIPEA in tetrahydrofuran (THF) afforded the 2′-*O*-AECM-G phosphoramidite **5** (Fig. 1B).

Synthesis of the 2′-*O*-(*N*-(aminoethyl)carbamoyl)methyluridine (2′-*O*-AECM-U) phosphoramidite began with preparation of *N*^3^-pivaloyloxymethyl-3′,5′-*O*-(1,1,3,3-tetraisopropyl-1,3-disiloxanediyl)uridine **6** [54] (Fig. 1C). The pivaloyloxymethyl (Pom) group was chosen for the protection of *N*^3^-position of uracil because of its stability under the alkylation conditions. Compound **6** was alkylated at the 2′-position with 1.3 equiv. of methyl 2-bromoacetate and 1.4 equiv. of the strong organic phosphazene base (*tert*-butylimino)tris(pyrrolidino)phosphorane (BTPP) to afford compound **7** in 92% yield. At this point, compound **7** was desilylated smoothly with triethylamine trihydrofluoride in THF.

Aminolysis of **8** was performed with ethylenediamine in methanol to give an intermediate, which was subsequently treated with aqueous ammonia to remove the Pom protection of the nucleobase. The crude product was further treated with ethyl trifluoroacetate in methanol to afford compound **9** (60% yield over three steps). Compound **9** was then selectively monomethoxytritylated at the 5′-position by treatment with MMTrCl in DMF–pyridine (3:2 v/v) to give **10,** which was further converted to the 2′-*O*-AECM-U phosphoramidite **11** (Fig. 1C).

The 2′-*O*-(*N*-(aminoethyl)carbamoyl)methylcytidine (2′-*O*-AECM-C) phosphoramidite was synthesized starting from 3′,5′-*O*-(1,1,3,3-tetraisopropyl-1,3-disiloxanediyl)cytidine (**12**) [55] (Fig. 1D). Protection of the exocyclic amino function with a benzoyl group under transient protection conditions before the alkylation step, as well as alkylation of **12** with the strong organic base BTPP, gave lower yields of the desired 2′-*O*-alkylated product. The highest yield was achieved by direct treatment of **12** with 1.1 equiv. of methyl 2-bromoacetate and 1.1 equiv. of sodium hydride to obtain compound **13** in 56% yield and some recovered **12**. Aminolysis of **13** with ethylenediamine in methanol followed by the trifluoroacetylation of the aminoethylcarbamoyl moiety afforded compound **14**.

The exocyclic amino group in **14** was acetylated and the crude product was desilylated using triethylamine trihydrofluoride to obtain base-protected compound **15** in good yield. This material was converted to 5′-*O*-4-methoxytrityl derivative **16,** which was further phosphitylated at the 3′-position with 2-cyanoethyl *N*,*N*-diisopropylphosphoramidochloridite in the presence of DIPEA in dichloromethane–acetonitrile (5:3 v/v) mixture to afford the 2′-*O*-AECM-C phosphoramidite **17**.

### Synthesis of 2′-O-AECM-modified ONs

Having the four different 2′-*O*-AECM building blocks, we proceeded with synthesis of the 18-mer, fully2′-*O*-AECM-modified ON (**ON1**) (Table 1), which was prepared on a 1 μmol scale using conventional automated solid-phase synthesis with 2-cyanoethyl phosphoramidite chemistry. BTT was used as activator in an extended 600 s coupling step. To ensure that no hydrolysis of the 2′-*O*-AECM group would occur [33], ethylenediamine in methanol, instead of aqueous ammonia, was used to release the ON from the solid support and remove all protecting groups. Crude **ON1** (Supplementary Fig. S18) was purified by RP-HPLC and characterized by electrospray ionization time-of flight (ESI-TOF) mass spectrometry (Supplementary Fig. S50). The RP-HPLC profile reveals that the ON synthesis protocol is quite satisfactory.

A series of fluorescein-labeled ONs was then prepared using the same synthesis methodology, but on a fluorescein-containing support. Thus, fluorescein-labeled fully 2′-*O*-AECM-modified 18-mer (**ON2**), 16-mer (**ON3**), and 12-mer (**ON4**) sequences, as well as the mixed 2′-*O*-AECM/2′-OMe-containing 12-mer sequences **ON5** and **ON6** were prepared (Table 1).

### Cellular uptake decreases with reduction of ON size and number of modifications

**FIG. 2.**
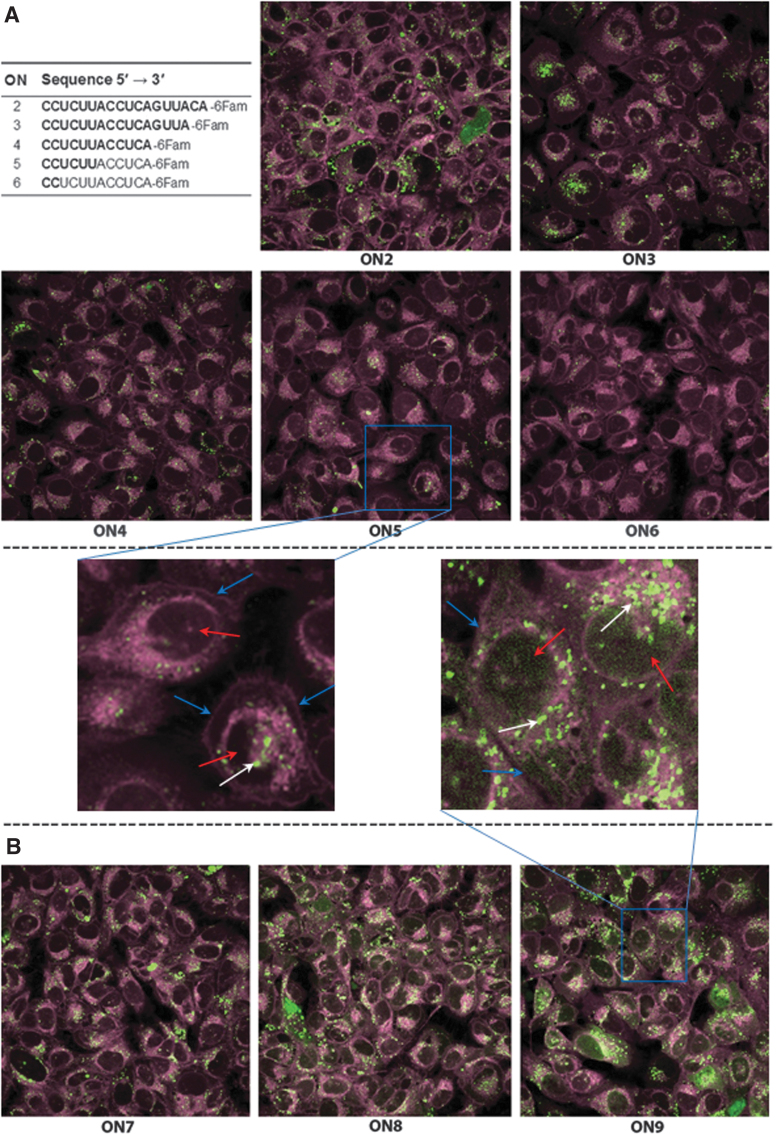
**(A)** Cellular uptake decreases with decreasing ON size and reduced number of 2′-*O*-AECM modifications. Confocal laser scanning microscopy images of U-2 OS cells treated with 4 μM of either fluorescein-labeled fully (**ON2**, **ON3**, and **ON4**) or partially (**ON5** and **ON6**) 2′-*O*-AECM-modified ONs for 24 h. **(B)** Inclusion of PS linkages result in a different, more diffuse pattern. Confocal laser scanning microscopy images of U-2 OS cells treated with 4 μM of either fluorescein-labeled partially (**ON7**) or fully PS with either a 2′-*O*-Me window (**ON8**) or fully 2′-*O*-AECM-modified (**ON9**) ONs for 24 h. Cells were washed before being processed for microscopy and the CellMask™ Deep Red dye was used to stain membrane structures (staining of cell membrane as well as intracellular membranes). In the zoomed areas, the *white arrows* indicate the fluorescein-labeled ONs visualized in *green*; the *blue arrows* indicate the outer boundaries of cells visualized in *pink*; the *red arrows* indicate the darker areas surrounded by membrane staining corresponding to the nucleus locations. Images represent the central plane of the cell in the *Z*-axis. ON, oligonucleotide; PS, phosphorothioate.

Cellular uptake of the fluorescein-labeled ONs was evaluated by confocal microscopy after incubation of U-2 OS cells with the respective ON for 24 h (Fig. 2). Based on the cellular density of the fluorescence from fluorescein-labeled ONs (in green), **ON2** is clearly taken up by the cells. Under these conditions, decreasing the length from 18 to 16 to a 12-mer of a 2′-*O*-AECM-modified ON resulted in lower levels of cellular uptake (**ON2**, **ON3**, and **ON4**). It is also evident that for ONs of the same length (12-mers), but with fewer (2 or 6 instead of 12) 2′-*O*-AECM modifications (**ON5** and **ON6**), the cellular uptake is reduced.

Taken together, these observations show that 2′-*O*-AECM modification substantially affects the ON internalization and that ON length is also important. The appearance of the ON intracellular distribution, that is, as dots rather than a diffuse distribution is similar to what was found for a 10-mer ON with only 2′-*O*-AECM-A [32]. This could indicate a higher concentration of ON in the endosomes.

### Confocal microscopy in U-2 OS cells with ONs carrying different combinations of 2′-O-AECM and 2′-OMe-modified ribonucleotides and PS backbone

To determine the influence of a partial or complete substitution of the phosphodiester backbone with PS, together with a combination of the 2′-*O*-AECM modification and 2′-OMe modification, ONs, **ON7**, **ON8**, and **ON9**, were synthesized (Table 1). The cellular uptake of **ON7**, **ON8**, and **ON9** was compared with the 2′-*O*-AECM-modified **ON2** and the 2′-OMe-PS-modified **ON10** (Table 1). The last one consists of both 2′-OMe groups and PS linkages throughout the sequence, which is a combination known to be taken up by cells [19,56] and thus used as a positive control.

Cellular uptake was visualized by confocal microscopy at two time points, 8 h and 24 h. All ONs reveal uptake, being more substantial at 24 h than at 8 h (Supplementary Fig. S77). As expected, and consistent with previous reports [19,56], after 8 h **ON10** shows cellular internalization, nuclear localization, and a more diffuse pattern, whereas the 2′-*O*-AECM-containing ONs (**ON2**, **ON7**, **ON8**, and **ON9**) also show uptake but the distribution is different and appear more in dots. A change in the distribution pattern toward a more diffuse pattern is observed as the 2′-*O*-AECM ONs are functionalized with an increasing amount of PS backbone linkages, especially for the longer incubation time (24 h) (Fig. 2B and Supplementary Fig. S77).

One should be cautious when interpreting the above results, since as previously reported for PS ONs [56], 2′-*O*-AECM-modified ON internalization and distribution within cells can also be time and cell line dependent. PS ONs are also known to associate with proteins [57] and the apparent distribution may then not represent the concentration of ON that is free to interact with the target. A functional assay may give a picture representing how much of the internalized ON is available, so the next experiment was to assess activity in a splice-switching cell assay.

### Splice-switching activity of 2′-O-AECM-modified ONs in different reporter cell lines

To obtain a better picture, representing how much of the internalized ON is available for the interaction with the target, the 2′-*O*-AECM-modified ONs were evaluated in functional assay for the splice-switching activity using the pLuc/705 splice-switching reporter system [58]. This standard assay has been normally used for evaluation of new modified ONs and delivery vectors and is based on a luciferase-encoding gene interrupted by a mutated β-globin intron 2. This mutation creates an aberrant 5′ splice site that activates a cryptic 3′ splice site, resulting in aberrant splicing of luciferase pre-mRNA and the translation of nonfunctional luciferase [58].

When an splice-switching oligonucleotide masks the aberrant site, splicing is redirected generating the correct mRNA and consequently the luciferase activity is restored. Recently, development and characterization of a small library of new, stable, pLuc/705 splice-switching reporter cell lines that represent different tissues [46] was described. Thus, the splice-switching activity of 2′-*O*-AECM-modified ONs was assessed in these different cell lines also to reflect ON uptake in different tissues.

Correlation between *in vitro* and *in vivo* studies is complex and requires the development of *in vitro* experimental conditions able to provide a better understanding of ON uptake and a reasonable interrelationship with *in vivo* results [59–61]. It has been shown that calcium ions enhance uptake of peptide nucleic acids (PNA) and PNA–peptide conjugates [62]. Most interestingly, studies from Hori *et al.* [63] demonstrate that medium with a somewhat higher calcium concentration than in serum potentiates an ON effect at lower concentrations. Recently, an evaluation of different ON modifications in different reporter cell lines arising from different tissues using this protocol for calcium supplementation was reported [46].

Thus, the splice-switching activity of 2′-*O*-AECM-modified ONs was evaluated using Ca^2+^ enrichment medium (CEM) method [63–66], which enhances naked ON delivery into cells by the addition of only 9 mM CaCl_2_ and allows to use lower concentrations of ONs compared with lower calcium concentration of the “gymnosis” method [59], in which higher concentrations of ON (normally >10 μM) are used. The CEM method also showed excellent positive correlation between ON activity in cell culture and mice, and can be used for the accurate prediction of *in vivo* gene silencing potency [63,67].

For the functional splice-switching assays, several 2′-*O*-AECM-containing ONs were prepared. Apart from the fully 2′-*O*-AECM-modified ON **ON1** with all linkages being nonmodified, that is, phosphodiesters, the combined 2′-*O*-AECM/2′-OMe ON **ON12**, the combined 2′-*O*-AECM/2′-OMe ONs with complete PS backbone **ON13**, the combined 2′-*O*-AECM/2′-OMe with partial PS backbone **ON14**, and the 2′-*O*-AECM ON with complete PS backbone **ON15** were synthesized (Table 1). To evaluate the influence of the modification it seems reasonable to make a first comparison with 2′-OMe oligoribonucleotides since the affinity to the target should then not be a major cause of any differences found. The affinity to the target RNA was evaluated and *T*_m_ analysis shows that there are relatively small differences between the different sequences. **ON1** displays an increase of 0.4°C per modification when compared with unmodified DNA (Table 1), a value quite similar to the one found for the fully 2′-OMe- and PS-modified sequence (**ON16**). It is interesting that the effect of the 2′-*O*-AECM modification on Δ*T*_m_ may be sequence and distribution dependent as variation between +0.4°C (in this study) and +2.3°C [32] per modification has been found so far.

**FIG. 3.**
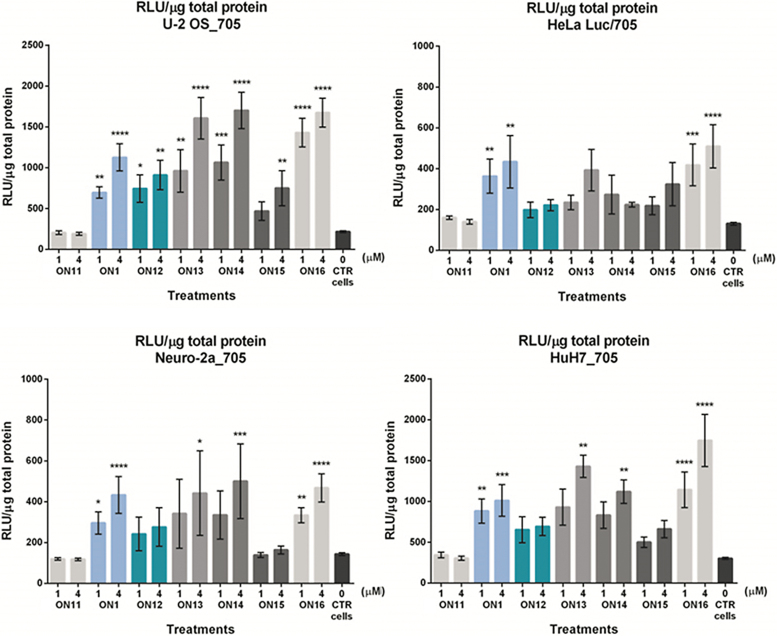
Luciferase production following splice correction with **ON1** or **ONs11-16** by “naked” delivery in Ca^2+^-enriched media is cell-type dependent. Graphs represent the RLU normalized by micrograms of total protein from U-2 OS_705, HeLa Luc/705 (*upper panel*), Neuro-2a_705, and HuH7_705 (*lower panel*) reporter cell lines when concentrations of 1 and 4 μM of the 2′-OMe **ON11**, 2′-OMe PS **ON16**, or 2′-*O*-AECM **ON1**, **ON12**, **ON13**, **ON14**, or **ON15** were delivered and the effect measured after 72 h. Data represent the mean ± SEM obtained from four independent experiments. *P* values calculated by one-way ANOVA test and comparison between treatment and untreated groups were determined by *post hoc* Fisher's LSD test. *P* < 0.05 was considered statistically significant. *P < 0.05; **P ≤ 0.01; ***P ≤ 0.001; ****P ≤ 0.0001. 2′-OMe, 2′-*O*-methyl; ANOVA, analysis of variance; LSD, Least Significant Difference; RLU, relative luminescence units; SEM, standard error of the mean.

The splice-switching activity of **ON1** and **ON11-16** (Table 1) was evaluated in the new reporter cell lines (C2C12_705, HuH7_705, Neuro-2a_705, and U-2 OS_705) [46] in parallel to the commonly used HeLa Luc/705 [58]. Figure 3 shows the luciferase readout normalized by total protein, which corresponds to the amount of mRNA correctly spliced, after treatment of different cell lines with the modified ONs at 1 or 4 μM for 72 h. In the difficult-to-transfect, nondifferentiated C2C12_705 cell line, no significant increase of luciferase was obtained, neither with the 2′-*O*-AECM-containing ONs nor with the 2′-OMe PS **ON16** (Supplementary Fig. S80). However, in all other cell lines a statistically significant, concentration-dependent increase of luciferase levels was found with the 2′-*O*-AECM-containing ONs.

In general, there are only small differences between the fully 2′-*O*-AECM-modified **ON 1** with a phosphodiester backbone and the fully modified 2′-OMe PS **ON16**, especially in the Neuro-2a_705 and the HeLa luc/705 cells where there is no significant difference in splice-switching activity detected (Fig. 3). Partial replacement of 2′-*O*-AECM with 2′-OMe modification (**ON12**) with a phosphate backbone in general gave a slightly lower activity, which is consistent with the confocal microscopy experiments with different 2′-*O*-AECM ON lengths and degree of incorporation of 2′-*O*-AECM modifications.

For the hepatocyte HuH7_705 and the U-2 OS_705 cell lines (Fig. 3), a somewhat higher activity was found with the 2′-OMe PS ON (**ON16**) compared with for **ON1**. For the U-2 OS_705 cell line, there was some benefit of combining 2′-*O*-AECM with full or partial PS modification in combination with partial replacement of 2′-*O*-AECM with 2′-OMe modifications (**ON13** and **ON14**).

In general, complete 2′-*O*-AECM and PS backbone modifications (**ON15**) gave somewhat lower activity, which could possibly correlate with the ON's *T*_m_ value. For Neuro-2a_705 and HeLa Luc/705 cells, there was no significant benefit of partial 2′-OMe and PS inclusion (**ON13** and **ON14**).

These results indicate that there is, in general, little additional advantage in combining the 2′-*O*-AECM modification with a PS backbone or in mixmers with 2′-OMe PS nucleotides. Even though the experimental data from confocal microscopy suggest that the incorporation of PS backbones improves the endosomal escape of AECM ONs (dotted vs. diffuse distribution pattern), the identified localization may not reflect availability of the ON to exert its desired action. It is possible that PS-containing ONs are at least partially associated with proteins, which makes them less available for binding to the target RNA, although they are seemingly escaping endosomes more readily. Additionally, other factors such as inclusion of a fluorophore, and experimental conditions may influence the experimental outcome.

If we attend to the *T*_m_ values of the different ONs, their small variation is an unlikely explanation for the differential effect on the splice-switching activity, except perhaps for **ON15**. The indication is rather that the activity differences are due to the cell type-dependent, intracellular uptake/availability of ONs. This is also consistent with the observation that splice-switching obtained with locked nucleic acid (LNA)-containing PS ONs that, despite their higher affinity to RNA, only give similar or lower activity in some of the cell line assays, as compared with 2′-OMe PS ONs [46].

### 2′-O-AECM-modified ON–ELL-peptide conjugate (ON18) and its splice-switching activity

It is generally considered that the endosomal escape barrier is one of the most major problems to the development of effective ON therapeutics [11]. Our above studies showed that AECM ONs can be effectively taken up by cells and give similar splice-switching activity as 2′-OMe PS ONs, however, it is likely that there is still a limitation in their release from endosomes (as indicated by the dotted pattern from confocal microscopy).

Neundorf *et al.* [68] demonstrated that the attachment of a short sequence of the hemagglutinin subunit HA2 [69] to CPPs increased membrane interaction and endosome disruption. Since the 2′-*O*-AECM ON can carry positive charges, in other words “a built-in CPP” (hence a cell-penetrating ON, CPO), we decided to investigate if a similar effect could be obtained as with CPPs, by attaching this type of peptide to 2′-*O*-AECM ONs. Thus, we synthesized an AECM ON conjugated to a short partially modified sequence of the HA2 (here called the ELL-peptide) and evaluated if this construct would give an increased splice-switching activity.

The synthesis was based on our methodologies for generation of ON–peptide conjugates on solid support that has proven to be, both, efficient and to reduce the number of purification steps [44,45], and recently the modified method was successfully employed for PS ONs [70].

The preparation of the 2′-*O*-AECM-modified ON–peptide conjugate (CPO-ELL or **ON18**, Table 1) includes the postsynthetic subsequent attachment of an aminolinker H-phosphonate [44], the alkyne group donor PAMBA [45], and the azido-functionalized peptide (ELL-peptide) (Supplementary Scheme S1). The CPG-supported **ON17** (Table 1), with free 5′-OH terminus, was first coupled with the aminolinker H-phosphonate, oxidized using a solution of iodine in pyridine–water, whereupon the amino group was detritylated. After attachment of the PAMBA residue, the ELL-peptide (made by the use of a standard microwave-assisted peptide synthesis protocol) was conjugated by Cu (I)-catalyzed cycloaddition. The conjugation of ELL-peptide to the CPG-supported ON under these conditions was effective resulting in the formation of a conjugate product (**ON18**) and disappearance of **ON17** functionalized with PAMBA residue (Supplementary Fig. S29).

**FIG. 4.**
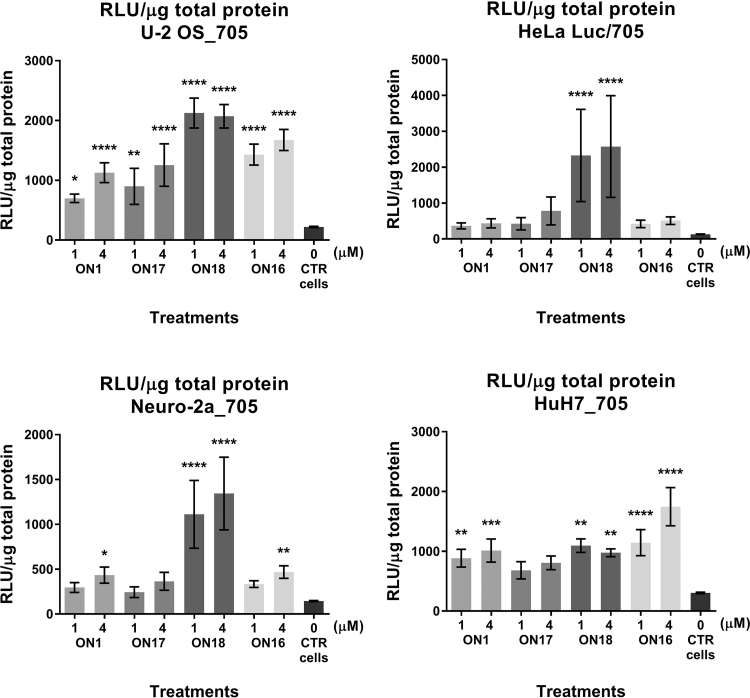
Luciferase production following splice correction with **ON1**, **ONs17-18,** or **ON16** by “naked” delivery in Ca^2+^-enriched media. Graphs represent the RLU normalized by micrograms of total protein from U-2 OS_705, HeLa Luc/705 (*upper panel*), Neuro-2a_705, and HuH7_705 (*lower panel*) reporter cell lines when concentrations of 1 and 4 μM of the 2′-*O*-AECM **ON1**, **ON17**, ELL-peptide conjugate **ON18** or 2′-OMe PS **ON16** were delivered and the effect measured after 72 h. Data represent the mean ± SEM obtained from four independent experiments. *P* values calculated by one-way ANOVA test and comparison between treatment and untreated groups were determined by *post hoc* Fisher's LSD test. *P* < 0.05 was considered statistically significant. *P < 0.05; **P ≤ 0.01; ***P ≤ 0.001; ****P ≤ 0.0001.

As shown in Fig. 4, an increase in the splice-switching activity of the CPO–ELL conjugate (**ON18**) is indeed obtained when compared with the nonpeptide conjugated correspondent ON (**ON17 = ON1** without the 3′-terminal 2′-*O*-AECM modification, which seems to make little difference in activity). This increase is modest in HuH7_705 cells but substantial in the other cell lines, especially in the Neuro-2a_705 and HeLa Luc/705 cells, where the effect is up to threefold. It is clear that in these cell lines the activity is considerably higher for **ON18** than for 2′-OMe PS ONs (**ON16**) even though the CPO–ELL has no PS linkages in the backbone. This, up to threefold increase in activity, indicates enhanced delivery and availability of CPO–ELL to the cellular site of action.

### Splice correction of a mutated *BTK* intron 4 by 2′-O-AECM-modified ONs and cytotoxicity

To further investigate the generality of the new AECM chemistry using another gene target, as well as different quantification methods, several other sequences of 2′-*O*-AECM-modified ONs and CPO–ELL-peptide conjugates without PS modification in the backbone were synthesized. These ONs were tested for splice-correction in a previously described U-2 OS model cell line for splice correction of a mutated *BTK* intron 4 known to cause X-linked agammaglobulinemia (XLA) [47]. In this system, the evaluation of the splice-correcting capacity was performed using RT-PCR. From the data obtained (Supplementary Data pp. S93–S95), it is clear that the AECM-modified phosphodiester-linked ON essentially is at par with the 2′-OMe PS ON despite the absence of PS linkages. Collectively, this shows efficient correction of two reporters with different readouts using ONs devoid of PS modification. To get a further indication of the therapeutic potential of 2′-*O*-AECM ONs, we also evaluated the cytotoxicity of several ONs and a monomer by WST-1 assays (Supplementary Data p. S96) and these seem to be rather safe when used in concentrations in the range of 4–10 μM in the tested cell lines.

## Conclusions

This study enables the preparation of 2′-*O*-AECM-modified ONs containing the most common ribonucleotides. We describe methodology for the preparation of 2′-*O*-AECM-modified nucleosides, the corresponding phosphoroamidite building blocks and the synthesis of 2′-*O*-AECM-modified oligoribonucleotides. In addition, we describe synthesis of a conjugate of 2′-*O*-AECM ONs with an endosomal escape peptide. Confocal microscopy studies in U-2 OS cells reveal that the 2′-*O*-AECM-modified ONs are taken up by cells, in the absence of transfection agents, and this internalization is ON size and modification degree dependent.

In functional assays based on the pLuc/705 splice-switching reporter system the 2′-*O*-AECM-modified ONs displayed high activity as SSOs in several cell lines (U-2 OS_705, HuH7_705, HeLa Luc/705, Neuro-2a_705) from different tissues.

The splice-switching efficiency of 2′-*O*-AECM-modified ONs was also demonstrated using a different gene target, a mutated *BTK* intron 4 in U-2 OS-mBTKi4 cell line. In both systems, 2′-*O*-AECM-containing ONs performed at par with the 2′-OMe PS ONs even without the incorporation of PS linkages, or with partial inclusion of PS modification, which is generally accepted and could be of importance in applications.

Since the 2′-*O*-AECM ONs also display high resistance to nucleases/phosphodiesterases and human serum [32,33], these results indicate that 2′-*O*-AECM modifications may be used to reduce the PS content in therapeutic ONs to balance the PS affinity to proteins [57] and possibly reduce PS ON toxicity. There are several reports indicating that functionality without or with only partial PS may be important since complement activation has been reported to be correlated to the length of PS single-stranded ONs [71]. PS gapmer ONs, in combination with 2′-ribose modifications, have also been reported to cause thrombocytopenia [34–36] and PS may also enhance immune stimulation [37,38].

On the other hand, 2′-*O*-AECM ONs may also benefit from inclusion of PS linkages, which affects retention time in the blood. Another interesting finding was that conjugation of a 2′-*O*-AECM ON with an endosomal escape peptide (the ELL-peptide) gave considerable improvement (up to threefold increase) of splice-switching activity over both unconjugated 2′-*O*-AECM ONs as well as over 2′-OMe PS ONs, especially for the Neuro-2a_705 and HeLa Luc/705 cell lines. Taken together, this signifies the high value of further exploring the potential of 2′-*O*-AECM ONs as therapeutic agents.

Further studies of the 2′-*O*-AECM-based CPOs are now enabled by the reported synthesis methodology and the results in the functional splice-switching assays suggest that it is highly interesting to explore this ON modification further in therapeutics. There is still much to investigate, to explore the potential of this new modification. This would include making 5-methyl pyrimidine building blocks, explore the potential in gapmers, and not least various conjugates such as improved endosomal escape entities.

Conjugates with other added entities, for example, homing peptides, sugars etc. could also impose different properties when attached to 2′-*O*-AECM ONs than to other modified ONs. It is also, for example, not unlikely that 2′-*O*-AECM ONs could be better suited for conjugation with positively charged peptides than fully polyanionic ONs, since known aggregation phenomena due to charge attraction [72] could be less of a problem here. Additional experiments, both *in vitro* and *in vivo*, should be performed and combinations with other nucleic acid modifications could also be explored to provide additional activity or tailored properties.

## Supplementary Material

Supplemental data
