# Supplementary material for: 2′-O-(N-(Aminoethyl)carbamoyl)methyl Modification Allows for Lower Phosphorothioate Content in Splice-Switching Oligonucleotides with Retained Activity
Source: Nucleic Acid Ther. 2022 Jun 1;32(3):221–33. doi: 10.1089/nat.2021.0086 (PMC9221157; doi:10.1089/nat.2021.0086)
Supplement: Supplemental data [file Supp_DataS1.pdf]

Supplementary Data

2′-*O*-Aminoethylcarbamoylmethyl (AECM) modification allows for lower phosphorothioate content in splice-switching oligonucleotides with retained activity.

Dmytro Honcharenko*, Cristina S. J. Rocha, Karin E. Lundin, Jyotirmoy Maity, Stefan Milton, Ulf Tedebark, Merita Murtola, Malgorzata Honcharenko, Andis Slaitas, C. I. Edvard Smith, Rula Zain, and Roger Strömberg*

CONTENTS:

Materials and Methods.……………………………………………………………………………………….………………...S2-S10

Figure S1-S11. ^{13}C APT NMR spectra of compounds **2-4**, **7-10** and **13-16**..……..………..……………………………………..…........S11-S21

Figure S12-S14. ^{19}F NMR spectra of compounds **3**, **9** and **14**..……..………..……………………………………………..…......S22-S24

Figure S15-S17. ^{31}P NMR spectra of compounds **5**, **11** and **17**..……..………..…………………………………………..…........S25-S27

Data S1. Purification details and analytical data for the synthesized ONs.……………………………………………………………..S28-S29

Figure S18-S28. Examples of HPLC profiles of crude 2′-*O*-AECM-modified **ONs**……………………………………………………...S30-S40

Figure S29. RP-HPLC profiles of conjugation steps during preparation of 2′-*O*-AECM-modified conjugate **ON18**……………………………S41

Figure S30-S49. RP-HPLC profiles of purified **ONs** ……………………………………………………………………………………..S42-S61

Figure S50-S69. ESI-TOF MS spectra of **ONs1-10**, **ONs12-15**, **ON17-20**, **ON22** and **ON23**………………………………………...........S62-S81

Figure S70-S76. Melting curve of corresponding duplex formed by **ON1** and **ONs11-16** with complementary RNA……………………..S82-S88

Figure S77. Confocal microscopy images of U-2 OS cells treated with 4 µM fluorescent-labelled **ON2** and **ONs7-10** for 8 h and 24 h............S89

Figure S78-S79. Synthesis procedure, ESI-TOF mass spectrum, and RP-HPLC chromatogram of 2′-*O*-AECM-U monomer……..………S90-S91

Figure S80. Luciferase production following splice-correction with **ON1** or **ONs11-18** in C2C12_705 reporter cell line.…………………….…S92

Splice-correction of a mutated *BTK* intron 4 by 2′-*O*-AECM modified ONs…………………………………………………………S93-S95

Cytotoxicity evaluation of 2′-O-AECM modified oligonucleotides and monomer……………………………………………………...S96

Scheme S1. Schematic representation of the synthesis of the 2′-*O*-AECM modified oligonucleotide–ELL-peptide conjugates………………S97

MATERIALS AND METHODS

General information

All reagents and solvents used for chemical synthesis were of commercial grade. Methanol (MeOH, Analytical Grade) and acetonitrile (MeCN, Analytical Grade) were additionally dried over 3Å molecular sieves and pyridine (Analytical Grade) over 4Å molecular sieves. Tetrahydrofuran (THF, *Puriss p.a.*) was distilled at atmospheric pressure over $LiAlH_4$ prior to use. Thin layer chromatography (TLC) was performed on Merck pre-coated silica gel 60 F254 glass-backed plates and visualized by UV and/or by charring with 8% (v/v) sulfuric acid in methanol or with 6% (m/v) ninhydrin in ethanol. Chromatographic separations were performed on Merck G60 silica gel. Mass analysis was performed with a Micromass LCT ESI-TOF mass spectrometer using leucine enkephalin as an internal mass standard. All NMR spectra were recorded on a Bruker AVANCE DRX 400 instrument (400.1 MHz in ^{1}H, 100.6 MHz in ^{13}C, 162.0 MHz in ^{31}P, and 376.5 MHz in ^{19}F) using either tetramethylsilane (TMS) or the deuterated solvent as an internal standard. Phosphoric acid (85%) for ^{31}P NMR and trifluoroacetic acid for ^{19}F NMR were used as external standards. Chemical shifts (δ scale) are reported in parts per million (ppm). Coupling constants (*J* values) are given in Hertz (Hz). HPLC was carried out on a Jasco HPLC system with UV detection at 254 nm or 280 nm (for the ELL-peptide). Reversed-phase (RP) HPLC was performed on a Supelco Discovery® BIO Wide Pore C18-5 (250 × 4.6 mm) column with 1 mL/min flow rate or on a Supelco Discovery® BIO Wide Pore C18-5 (250 × 10 mm) column with 4 mL/min flow rate with a linear gradient from 0 to 50% of buffer B in buffer A over 45 min at 50 °C or as specified. Buffers for RP-HPLC were as follows: (A) 50 mM triethylammonium acetate (TEAA), pH 6.5; (B) 50 mM TEAA, pH 6.5, in 50% CH_3CN. Purification and analysis of peptide were performed on a Phenomenex Jupiter® 4 µm Proteo 90 Å (250 × 4.6 mm) column with 1 mL/min flow rate and linear gradient elution of 40% to 100% of solvent B in solvent A in 40 min (solvent A = 0.1% TFA in water; solvent B = 0.1% TFA in 90% aqueous acetonitrile) using detection at 280 nm and temperature 25 °C. Ion-exchange (IE) HPLC was performed on a Dionex, NucleoPac™ PA-100 (250 × 4 mm) column with 1.5 mL/min flow rate with a linear gradient from 0 to 40% of buffer B in buffer A over 30 min at 60 °C. Buffers for IE-HPLC were as follows: (A) 20 mM sodium acetate (NaOAc) in 30% CH_3CN; (B) 20 mM NaOAc, 0.4 M lithium perchlorate ($LiClO_4$) in 30% CH_3CN. ONs: native complementary RNA, 5′-UGUAACUGAGGUAAGAGG-3′, and DNA, 5′-d(CCTCTTACCTCAGTTACA)-3′, for comparison of thermal melting points with the target RNA were purchased from Eurogentec S.A., Belgium. **ON11**, 5′-CCUCUUACCUCAGUUACA-3′, was purchased from Rasayan Inc. **ON21**, 5′-C*A*G*A*G*U*U*C*U*C*A*G*G*A*U*G*U*A-3′, was purchased from Sigma-Aldrich. **ON16**, 5′-C*C*U*C*U*U*A*C*C*U*C*A*G*U*U*A*C*A-3′, was obtained from GE Healthcare Bio-Sciences AB (Sweden). **ON24**, 5′-C*C*G*C*U*G*G*U*C*C*U*C*A*G*G*A*A*C-3′, was purchased from DNA Technology A/S (Denmark). After being dissolved in nuclease-free water, the concentration was assessed using a NanoDrop (Thermo Scientific) and stored as frozen aliquots

at −20 °C. ONs were analyzed in negative mode as solutions in acetonitrile/water, 1:1 (v/v) and their molecular weights were obtained from the *m/z* values of the multiply charged ions.

Synthesis of building blocks

2′-*O*-((*N*, *N*-Aminoethyleneamine)carbonylmethylene)guanosine (2). Compound **1** (0.14 g, 0.39 mmol) was suspended in anhydrous methanol (4 mL) at room temperature, ethylenediamine (0.26 mL, 3.9 mmol) was added and the reaction mixture was stirred for 20 h. Volatiles were evaporated *in vacuo* and an added toluene-methanol mixture was evaporated to afford an intermediate which was dissolved in Milli-Q water (6.8 mL, pH 7.5) and further treated with adenosine deaminase [3.0 µL (3 units) of aqueous glycerol solution, 151 units/mg protein, 11 mg/mL] at room temperature for 48 h. The resulting mixture was freeze-dried to give compound **2** (0.15 g, 0.39 mmol) in quantitative yield. R_f = 0.17 (EtOAc/CH_3OH/AcOH/H_2O 10:2:2:1 v/v/v/v). ^{1}H NMR (400.1 MHz, DMSO-d_6): δ = 7.95 (s, 1 H, 8-H), 7.94-7.88 (m, 1 H, N*H*-ethylene), 6.51 (br s, 2 H, NH_2), 5.86 (d, *J* = 5.37 Hz, 1 H, 1′-H), 4.34 (t, *J* = 5.11 Hz, 1 H, 2′-H), 4.28-4.24 (m, 1 H, 3′-H), 4.06-3.91 (m, 3 H, 4′-H, CH_2-carbamoyl), 3.63 (dd, *J* = 11.94, 3.66 Hz, 1 H, 5′-Ha), 3.54 (dd, *J* = 12.01, 3.90 Hz, 1 H, 5′-Hb), 3.18-3.01 (m, 2 H, CH_2-ethylene), 2.58 (t, *J* = 6.37 Hz, 2 H, CH_2-ethylene) ppm. ^{13}C NMR (100.6 MHz, DMSO-d_6): δ = 169.0, 156.6, 153.7, 150.9, 136.6, 116.6, 85.0, 84.6, 82.7, 69.4, 68.8, 60.9, 41.4, 41.0 ppm; ES–MS calcd. for $C_{14}H_{20}N_7O_6$ [M – H]$^-$ 382.15, found 382.25.

***N*^{2}-(Phenoxyacetyl)-2′-*O*-(*N*-(trifluoroacetamidoethyl)carbamoyl)methylguanosine (3).** Ethyl trifluoroacetate (0.43 mL, 3.65 mmol) was added to a suspension of compound **2** (0.14 g, 0.365 mmol) in anhydrous methanol (7.2 mL) and the reaction mixture was stirred at room temperature for 24 h. Volatiles were evaporated *in vacuo* and the residue was redissolved in methanol, which was then evaporated to give a crude intermediate. The resulting product was rendered anhydrous by evaporation of added anhydrous pyridine and then dissolved in 7.2 mL of the same solvent. To the resulting solution chlorotrimethylsilane (0.23 mL, 1.82 mmol) was added dropwise and the reaction mixture was stirred at ambient temperature for 3 h under nitrogen atmosphere. Phenoxyacetic anhydride (0.157 g, 0.547 mmol) was added and the reaction was kept at room temperature for 22 h. The reaction mixture was chilled in an ice-water bath and water (1.3 mL) was added. The resulting mixture was allowed to stir at room temperature overnight. The reaction mixture was partitioned between water and ethyl acetate. The combined organic phases were dried over Na_2SO_4, filtered and concentrated under reduced pressure. The residue was subjected to column chromatography using 0 to 12% methanol in dichloromethane as eluent to give compound **3** (0.15 g, 0.244 mmol, 84%). R_f = 0.61 (CH_2Cl_2/CH_3OH 4:1 v/v). ^{1}H NMR (400.1 MHz, CD_3OD): δ = 8.32 (s, 1 H, 8-H), 7.35-7.28 (m, 2 H, phenoxyacetyl), 7.06-6.98 (m, 3 H, phenoxyacetyl), 6.12 (d, *J* = 3.2 Hz, 1 H, 1′-H), 4.82 (s, 2 H, CH_2-phenoxyacetyl), 4.49-4.45 (m, 1 H, 3′-H), 4.38-4.34 (m, 1 H, 2′-H), 4.27 (ABq, *J* = 15.5 Hz 2 H, CH_2-carbamoyl), 4.15-4.10 (m, 1 H, 4′-H), 3.93 (dd, *J* = 12.4, 2.5 Hz, 1 H, 5′-Ha), 3.79 (dd, *J* = 12.3, 3.2 Hz, 1 H, 5′-Hb), 3.46-3.35 (m, 4 H, 2CH_2-ethylene) ppm. ^{13}C NMR (100.6 MHz, CD_3OD): δ = 172.8, 159.9, 159.6 (q, *J* = 36.9 Hz), 159.1, 149.7, 149.0, 139.6, 130.7, 123.1, 121.9, 117.5 (q, *J* = 286.7 Hz), 115.9, 88.4,

86.3, 85.3, 70.8, 70.1, 68.2, 61.7, 40.3, 39.2 ppm. ^{19}F NMR (376.5 MHz, CD_3OD): δ = -76.1 ppm; ES–MS calcd. for $C_{24}H_{25}F_3N_7O_9$ $[M - H]^-$ 612.17, found 612.42.

5′-*O*-(4-Methoxytrityl)-*N*^{2}-(phenoxyacetyl)-2′-*O*-(*N*-(trifluoroacetamidoethyl)carbamoyl)methyl-guanosine (4). Compound **3** (0.046 g, 0.075 mmol) was dried by evaporation of added anhydrous pyridine (2 times) and dissolved in 1.2 mL of anhydrous *N*,*N*-dimethylformamide(DMF)-dimethyl sulfoxide(DMSO)-pyridine (2:1:1 v/v/v) mixture. To the resulting solution triethylamine (0.031 mL, 0.225 mmol) was added followed by 4-methoxytritylchloride (0.116 g, 0.375 mmol) and the reaction mixture was stirred at ambient temperature for 19 h. The reaction was quenched with saturated aqueous $NaHCO_3$ and the mixture was extracted with ethyl acetate. The organic phase was washed with water, dried over Na_2SO_4, filtered and concentrated under reduced pressure. The residue was subjected to column chromatography using 0 to 10% methanol in dichloromethane as eluent to give compound **4** (0.051 g, 0.057 mmol, 77%). R_f = 0.53 (CH_2Cl_2/CH_3OH 9:1 v/v). ^{1}H NMR (400.1 MHz, CD_3OD): δ = 8.07 (s, 1 H, 8-H), 7.44-7.38 (m, 4 H, trityl), 7.32-7.15 (m, 10 H, trityl, phenoxyacetyl), 7.03-6.96 (m, 3 H trityl, phenoxyacetyl), 6.80 (d, *J* = 8.8 Hz, 2 H, phenoxyacetyl), 6.12 (d, *J* = 2.6 Hz, 1 H, 1′-H), 4.78 (s, 2 H, C*H*$_2$-phenoxyacetyl), 4.59-4.54 (m, 1 H, 3′-H), 4.49 (dd, *J* = 4.9, 2.7 Hz, 1 H, 2′-H), 4.35-4.21 (m, 3 H, C*H*$_2$-carbamoyl, 4′-H), 3.74 (s, 3 H, OC*H*$_3$-trityl), 3.47-3.34 (m, 6 H, 2C*H*$_2$-ethylene, 5′-Ha, 5′-Hb) ppm. ^{13}C NMR (100.6 MHz, CD_3OD): δ = 172.8, 172.7, 160.3, 159.0, 159.4 (q, *J* = 37.0 Hz), 149.6, 148.8, 145.7, 145.6, 136.5, 131.6, 130.7, 129.6, 128.9, 128.1, 123.1, 122.2, 117.5 (q, *J* = 286.7 Hz), 116.0, 114.2, 88.7, 88.1, 84.7, 84.5, 70.9, 70.7, 68.2, 64.3, 55.8, 40.3, 39.2 ppm.; ES–MS calcd. for $C_{44}H_{41}F_3N_7O_{10}$ $[M - H]^-$ 884.29, found 884.93.

3′-*O*-(*N*,*N*-Diisopropylamino-(2-cyanoethoxy)phosphinyl)-5′-*O*-(4-methoxytrityl)-*N*^{2}-(phenoxy-acetyl)-2′-*O*-[(*N*-(trifluoroacetamidoethyl)carbamoyl)methyl]guanosine (5). Compound **4** (0.708 g, 0.8 mmol) was dried by evaporation of added anhydrous THF and dissolved in 8 mL of the same solvent. To the resulting chilled (ice bath) solution, *N*,*N*′-diisopropylethylamine (0.7 mL, 4 mmol) was added under nitrogen atmosphere followed by 2-cyanoethyl *N*,*N*-diisopropylphosphoramidochloridite (0.36 mL, 1.6 mmol). After 30 min, the ice bath was removed and the reaction mixture was stirred for another 3 h. The reaction was quenched by the addition of methanol (0.5 mL), and the solvent was partially removed under reduced pressure. The residue was partitioned between ethyl acetate and a 10% aqueous solution of $NaHCO_3$, and the aqueous phase was re-extracted with ethyl acetate. The combined organic phases were dried over Na_2SO_4, filtered and concentrated under reduced pressure. The crude product was purified by flash column chromatography using 0 to 10% 2-propanol in dichloromethane containing 0.1% of triethylamine as eluent to give **5** (0.69 g, 77%). R_f = 0.50 (CH_2Cl_2/CH_3OH 16:1 v/v). ^{31}P NMR (162.0 MHz, $CDCl_3$): δ = 150.1, 147.8 ppm; HRMS (ESI-TOF): calcd. for $C_{53}H_{58}F_3N_9O_{11}P$ $[M - H]^-$ 1084.3951, found 1084.3955.

2′-*O*-(*O*-Methylcarboxymethyl)-*N*^{3}-pivaloyloxymethyl-3′,5′-*O*-(1,1,3,3-tetraisopropyl-1,3-di-siloxanediyl)uridine (7). *N*^{3}-Pivaloyloxymethyl-3′,5′-*O*-(tetraisopropyldisiloxan-1,3-diyl)uridine (compound **6**) (6.98 g, 11.62 mmol) was dried by evaporation of added anhydrous CH_3CN and dissolved in 120 mL of

the same solvent. Methyl 2-bromoacetate (1.43 mL, 15.11 mmol) was added dropwise to the stirred solution under nitrogen atmosphere followed by the addition of phosphazene base P_1-*tert*-butyl-tris(tetramethylene) (4.97 mL, 16.27 mmol). The reaction mixture was stirred at ambient temperature for 2 h. Volatiles were evaporated to dryness *in vacuo* and toluene was added to the residue and evaporated. The crude product was purified *via* flash chromatography using 0 to 25% ethyl acetate in hexane as eluent to afford compound **7** (7.19 g, 10.68 mmol, 92%). R_f = 0.63 (EtOAc/toluene 3:7 v/v). ^{1}H NMR (400.1 MHz, $CDCl_3$): δ = 7.90 (d, J = 8.2 Hz, 1 H, 6-H), 5.96, 5.92 (ABq, J = 9.4 Hz, 2 H, CH_2OCO^{t}Bu), 5.80 (s, 1 H, 1′-H), 5.73 (d, J = 8.2 Hz, 1 H, 5-H), 4.57, 4.42 (ABq, J = 16.5 Hz, 2 H, CH_2-carbamoyl), 4.29-4.21 (m, 3 H, 2′-H, 3′-H, 5′-Ha), 4.03-3.95 (m, 2 H, 4′-H, 5′-Hb), 3.75 (s, 3 H, CH_2COOCH_3), 1.19 (s, 9 H, ^{t}Bu), 1.13-1.01 (m, 28 H, ^{i}Pr) ppm. ^{13}C NMR (100.6 MHz, $CDCl_3$): δ = 177.4, 170.3, 161.6, 150.0, 138.3, 101.1, 89.0, 82.5, 81.6, 68.3, 67.4, 64.4, 59.3, 51.8, 27.0, 17.5, 17.4, 17.3, 17.2, 16.95, 16.92, 16.7, 13.4, 13.1, 12.9, 12.3 ppm; ES–MS calcd. for $C_{30}H_{52}N_2NaO_{11}Si_2$ [M + Na]$^+$ 695.30, found 695.55.

2′-*O*-(*O*-Methylcarboxymethyl)-*N*^{3}-pivaloyloxymethyluridine (8). To a solution of compound **7** (6.96 g, 10.34 mmol) in 90 mL of anhydrous tetrahydrofuran, triethylamine trihydrofluoride (4.04 mL, 24.82 mmol) was added under nitrogen atmosphere and the reaction mixture was stirred at room temperature for 2 h. Volatiles were evaporated *in vacuo*, methanol and dichloromethane were added and subsequently evaporated. The crude product was purified *via* flash chromatography using 0 to 6% methanol in dichloromethane as eluent to afford compound **8** (4.18 g, 9.71 mmol, 94%). R_f = 0.59 (CH_2Cl_2/CH_3OH 9:1 v/v). ^{1}H NMR (400.1 MHz, $CDCl_3$): δ = 7.84 (d, J = 8.2 Hz, 1 H, 6-H), 5.93 (ABq, J = 9.6 Hz, 2 H, CH_2OCO^{t}Bu), 5.80-5.75 (m, 2 H, 1′-H, 5-H), 4.48, 4.32 (ABq, J = 17.1 Hz, 2 H, CH_2-carbamoyl), 4.27 (t, J = 5.4 Hz, 1 H, 3′-H), 4.18-4.12 (m, 2 H, 2′-H, 3′-OH), 4.06-3.99 (m, 1 H, 5′-Ha), 3.98 (d, J = 5.4 Hz, 1 H, 4′-H), 3.86 (ddd, J = 12.2, 6.3, 1.7 Hz, 1 H, 5′-Hb), 3.78 (s, 3 H, CH_2COOCH_3), 2.93 (dd, J = 5.9, 4.0 Hz, 1 H, 5′-OH), 1.19 (s, 9 H, ^{t}Bu) ppm. ^{13}C NMR (100.6 MHz, $CDCl_3$): δ = 177.5, 171.9, 161.6, 150.5, 140.2, 101.7, 90.7, 84.8, 84.1, 68.4, 68.2, 64.6, 61.1, 52.5, 27.0 ppm; ES–MS calcd. for $C_{18}H_{26}N_2NaO_{10}$ [M + Na]$^+$ 453.15, found 453.60.

2′-*O*-[(*N*-(Trifluoroacetamidoethyl)carbamoyl)methyl]uridine (9). Ethylenediamine (3.17 mL, 47.50 mmol) was added to the solution of compound **8** (4.09 g, 9.50 mmol) in 65 mL of anhydrous methanol at room temperature and the reaction mixture was stirred for 20 h. Volatiles were evaporated *in vacuo* and added toluene-methanol and dichloromethane were evaporated. The crude residue was treated with 25% aqueous ammonia (120 mL) at ambient temperature for 20 h. Water was partially evaporated under reduced pressure and resulting residue was freeze-dried. The fully deprotected intermediate was dissolved in anhydrous methanol (90 mL) and ethyl trifluoroacetate (13.8 mL, 116.20 mmol) was added, and the reaction mixture was stirred for 23 h at room temperature. Volatiles were evaporated *in vacuo* and the crude residue was purified *via* flash chromatography using 0 to 16% methanol in dichloromethane as eluent to afford compound **9** (2.51 g, 5.70 mmol, 60%). R_f = 0.57 (CH_2Cl_2/CH_3OH 4:1 v/v). ^{1}H NMR (400.1 MHz, CD_3OD): δ = 8.10 (d, J = 8.1 Hz, 1 H, 6-H), 5.93 (d, J = 2.4 Hz, 1 H, 1′-H), 5.68 (d, J = 8.1 Hz, 1 H, 5-H), 4.31-4.18 (m, 3 H, 3′-H, CH_2-carbamoyl), 4.07-4.00 (m, 2 H, 2′-H, 4′-H), 3.93 (dd, J = 12.4, 2.1 Hz, 1 H, 5′-Ha), 3.77 (dd, J = 12.4, 2.7 Hz, 1 H, 5′-Hb), 3.44 (s, 4 H, 2CH_2-ethylene) ppm. ^{13}C NMR (100.6 MHz, CD_3OD): δ = 172.8, 166.3, 159.4 (q, J = 36.9 Hz), 152.3,

142.0, 117.5 (q, J = 286.6 Hz), 102.4, 89.7, 85.6, 85.0, 70.8, 69.6, 61.1, 40.4, 39.1 ppm. ^{19}F NMR (376.5 MHz, CD_3OD): δ = -77.1 ppm; ES–MS calcd. for $C_{15}H_{18}F_3N_4O_8$ [M – H]$^-$ 439.11, found 439.35.

5′-*O*-(4-Methoxytrityl)-2′-*O*-[(*N*-(trifluoroacetamidoethyl)carbamoyl)methyl]uridine (10). Compound **9** (2.22 g, 5.04 mmol) was dried by evaporation of added anhydrous pyridine and dissolved in 50 mL of anhydrous DMF-pyridine (3:2 v/v) mixture. To the resulting solution, 4-methoxytritylchloride (1.87 g, 6.05 mmol) was added under nitrogen atmosphere and the reaction mixture was stirred at ambient temperature for 24 h. Solvents were partially removed under reduced pressure, cold saturated aqueous $NaHCO_3$ was added to the residue and the mixture was extracted with ethyl acetate. The organic phase was washed with water, dried over Na_2SO_4, filtered and concentrated under reduced pressure. Traces of pyridine were removed by evaporation of added toluene. The crude product was subjected to column chromatography using 0 to 10% methanol in dichloromethane as eluent to give compound **10** (2.98 g, 4.18 mmol, 83%). R_f = 0.54 (CH_2Cl_2/CH_3OH 9:1 v/v). ^{1}H NMR (400.1 MHz, CD_3OD): δ = 8.05 (d, J = 8.1 Hz, 1 H, 6-H), 7.46-7.41 (m, 4 H, trityl), 7.34-7.23 (m, 8 H, trityl), 6.88 (d, J = 8.9 Hz, 2 H, trityl), 5.88 (d, J = 1.1 Hz, 1 H, 1′-H), 5.20 (d, J = 8.1 Hz, 1 H, 5-H), 4.51 (dd, J = 8.6, 5.1 Hz, 1 H, 3′-H), 4.34, 4.25 (ABq, J = 15.8 Hz, 2 H, CH_2-carbamoyl), 4.17-4.11 (m, 1 H, 4′-H), 4.03 (dd, J = 5.1, 1.1 Hz, 1 H, 2′-H), 3.78 (s, 3 H, OCH_3-trityl), 3.54 (d, J = 2.5 Hz, 2 H, 5′-Ha, 5′-Hb), 3.44 (s, 4 H, 2CH_2-ethylene) ppm. ^{13}C NMR (100.6 MHz, CD_3OD): δ = 172.8, 166.2, 160.5, 159.4 (q, J = 36.8 Hz), 152.0, 145.6, 145.3, 141.8, 136.1, 131.8, 129.7, 129.0, 128.32, 128.3, 117.5 (q, J = 286.7 Hz), 114.3, 102.3, 90.0, 88.5, 85.1, 83.7, 70.9, 69.7, 62.5, 55.8, 40.4, 39.1 ppm; ES–MS calcd. for $C_{35}H_{34}F_3N_4O_9$ [M – H]$^-$ 711.23, found 711.71.

3′-*O*-(*N*,*N*-Diisopropylamino-(2-cyanoethoxy)phosphinyl)-5′-*O*-(4-methoxytrityl)-2′-*O*-[(*N*-(trifluoroacetamidoethyl)carbamoyl)methyl]uridine (11). Compound **10** (1.71 g, 2.4 mmol) was dried by evaporation of added anhydrous THF and dissolved in 24 mL of the same solvent. To the resulting chilled (ice bath) solution, *N*,*N*′-diisopropylethylamine (2.09 mL, 12 mmol) was added under nitrogen atmosphere followed by 2-cyanoethyl *N*,*N*-diisopropylphosphoramidochloridite (1.07 mL, 4.8 mmol). After 30 min, the ice bath was removed and the reaction mixture was stirred for another 2 h. The reaction was quenched by the addition of methanol (1.2 mL) and solvent was partially removed under reduced pressure. The residue was partitioned between ethyl acetate and 10% aqueous solution of $NaHCO_3$ and the aqueous phase was re-extracted with ethyl acetate. The combined organic phases were dried over Na_2SO_4, filtered and concentrated under reduced pressure. The crude product was purified *via* flash column chromatography using 0 to 10% acetonitrile in ethyl acetate containing 0.1% of triethylamine as eluent to give **11** (1.77 g, 81%). R_f = 0.50 (CH_2Cl_2/CH_3OH 15:1 v/v). ^{31}P NMR (162.0 MHz, $CDCl_3$): δ = 152.1, 148.9 ppm; HRMS (ESI-TOF): calcd. for $C_{44}H_{51}F_3N_6O_{10}P$ [M – H]$^-$ 911.3362, found 911.3361.

2′-*O*-(*O*-Methylcarboxymethyl)-3′,5′-*O*-(1,1,3,3-tetraisopropyl-1,3-disiloxanediyl)cytidine (13). 3′,5′-*O*-(1,1,3,3-Tetraisopropyl-1,3-disiloxanediyl)cytidine (compound **12**) (0.5 g, 1.03 mmol) was dissolved in anhydrous DMF (10 mL). The solution was chilled in an ice-water bath, and NaH (60% dispersion in mineral oil, 0.045 g, 1.13 mmol) was added under nitrogen atmosphere. The reaction mixture was stirred for 30 min, methyl 2-bromoacetate (0.107 mL, 1.13

mmol) was added dropwise, and the reaction mixture was stirred for 3 h. An additional portion of NaH (60% dispersion in mineral oil, 0.008 g, 0.2 mmol) and methyl 2-bromoacetate (0.019 mL, 0.2 mmol) were added to the reaction mixture, chilled in an ice-water bath, and stirred for an additional 1 h. Glacial acetic acid (0.1 mL) was added into the reaction mixture and after stirring for 10 min the volatiles were evaporated *in vacuo*. The mixture was dissolved in ethyl acetate and the organic phase was washed with water (2 times), dried over Na_2SO_4, filtered and the solvents were evaporated. The residue was subjected to flash column chromatography using 0 to 6% methanol in dichloromethane as eluent to give compound **13** (0.32 g, 0.57 mmol, 56%). R_f = 0.63 (CH_2Cl_2/CH_3OH 9:1 v/v) and recovered starting material **12** (0.082 g). ^1H NMR (400.1 MHz, DMSO-d_6): δ =7.69 (d, J = 7.4 Hz, 1 H, 6-H), 7.20 (br s, 2 H, NH_2-base), 5.69 (d, J = 7.4 Hz, 1 H, 5-H), 5.65 (s, 1 H, 1′-H), 4.56, 4.43 (ABq, J = 16.5 Hz, 2 H, CH_2-carbamoyl), 4.22-4.13 (m, 2 H, 3′-H, 5′-Ha), 4.07-3.98 (m, 2 H, 2′-H, 4′-H), 3.91 (dd, J = 13.5, 2.2 Hz, 1 H, 5′-Hb), 3.64 (s, 3 H, CH_2COOCH_3), 1.11-0.91 (m, 28 H, ^{i}Pr) ppm. ^{13}C NMR (100.6 MHz, DMSO-d_6): δ = 170.1, 165.7, 154.6, 139.6, 93.3, 88.2, 81.4, 80.4, 68.1, 66.3, 59.5, 51.3, 17.2, 17.13, 17.1, 17.0, 16.7, 16.67, 16.56, 12.6, 12.3, 12.2, 11.7 ppm; ES–MS calcd. for $C_{24}H_{42}N_3O_8Si_2$ $[M - H]^-$ 556.25, found 556.40.

2′-*O*-(*N*-(Trifluoroacetamidoethyl)carbamoyl)methyl-3′,5′-*O*-(1,1,3,3-tetraisopropyl-1,3-di-siloxanediyl)cytidine (14). Compound **13** (0.3 g, 0.54 mmol) was dissolved in anhydrous methanol (5 mL), ethylenediamine (0.18 mL, 2.69 mmol) was added at room temperature and the reaction mixture was stirred for 19 h. Volatiles were evaporated *in vacuo*, toluene and dichloromethane were added and then evaporated. The crude residue was dissolved in anhydrous methanol (5 mL), ethyl trifluoroacetate (0.3 mL, 2.51 mmol) was added and the reaction mixture was stirred at room temperature for 20 h. Volatiles were evaporated *in vacuo* and the residue was purified *via* flash chromatography using 0 to 10% methanol in dichloromethane as eluent to afford compound **14** (0.250 g, 0.34 mmol, 68%). R_f = 0.55 (CH_2Cl_2/CH_3OH 9:1 v/v). ^1H NMR (400.1 MHz, DMSO-d_6): δ = 9.50 (t, J = 5.0 Hz, 1 H, NH-ethylene), 7.89 (t, J = 5.8 Hz, 1 H, NH-ethylene), 7.65 (d, J = 7.4 Hz, 1 H, 6-H), 7.25 (br s, 2 H, NH_2-base), 5.73 (d, J = 7.4 Hz, 1 H, 5-H), 5.70 (s, 1 H, 1′-H), 4.27-4.13 (m, 4 H, 3′-H, 5′-Ha, CH_2-carbamoyl), 4.12-4.06 (m, 1 H, 4′-H), 4.01 (d, J = 4.3 Hz, 1 H, 2′-H), 3.93 (dd, J = 13.5, 2.0 Hz, 1 H, 5′-Hb), 3.45-3.26 (m, 3 H, CH_2-ethylene, CH_2a-ethylene), 3.24-3.14 (m, 1 H, CH_2b-ethylene), 1.10-0.91 (m, 28 H, ^{i}Pr) ppm. ^{13}C NMR (100.6 MHz, DMSO-d_6): δ = 169.4, 165.8, 156.4 (q, J = 35.7 Hz), 154.9, 139.1, 115.8 (q, J = 289.0 Hz), 93.6, 88.9, 82.0, 80.3, 69.5, 68.7, 59.3, 39.0, 37.2, 17.2, 17.1, 17.05, 17.0, 16.8, 16.74, 16.7, 16.6, 12.6, 12.24, 12.21, 12.0 ppm. ^{19}F NMR (376.5 MHz, DMSO-d_6): δ = -76.0 ppm; ES–MS calcd. for $C_{27}H_{45}F_3N_5O_8Si_2$ $[M - H]^-$ 680.28, found 680.51.

***N*^{4}-Acetyl-2′-*O*-[(*N*-(trifluoroacetamidoethyl)carbamoyl)methyl]cytidine (15).** Acetic anhydride (0.145 mL, 1.54 mmol) was added to a solution of **14** (0.21 g, 0.31 mmol) in anhydrous pyridine (4 mL) and the reaction mixture was stirred at room temperature for 36 h. The reaction was quenched by 10% aqueous $NaHCO_3$ and the mixture was extracted with dichloromethane. The organic phase was dried over Na_2SO_4, filtered and concentrated under reduced pressure. The crude residue (0.195 g) was dissolved in anhydrous tetrahydrofuran-acetonitrile (6 mL, 1:1), triethylamine trihydrofluoride (0.105 mL, 0.65 mmol) was added and the reaction mixture was stirred at room temperature for 4 h. Volatiles were evaporated *in vacuo*, and added toluene and dichloromethane were

evaporated. The crude product was purified *via* flash chromatography using 0 to 15% methanol in dichloromethane as eluent to afford compound **15** (0.122 g, 0.25 mmol, 95%). R_f = 0.21 (CH_2Cl_2/CH_3OH 9:1 v/v). ^1H NMR (400.1 MHz, DMSO-d_6): δ = 10.92 (s, 1 H, N*H*-Ac), 9.47 (br t, 1 H, N*H*-ethylene), 8.47 (d, *J* = 7.5 Hz, 1 H, 6-H), 8.14 (br t, 1 H, N*H*-ethylene), 7.20 (d, *J* = 7.5 Hz, 1 H, 5-H), 5.81 (s, 1 H, 1′-H), 5.42 (d, *J* = 7.5 Hz, 1 H, 3′-OH), 5.23 (t, *J* = 5.0 Hz, 1 H, 5′-OH), 4.26, 4.11 (ABq, *J* = 15.6 Hz, 2 H, CH_2-carbamoyl), 4.10-4.02 (m, 1 H, 3′-H), 3.99-3.93 (m, 1 H, 4′-H), 3.90 (d, *J* = 5.0 Hz, 1 H, 2′-H), 3.84 (ddd, *J* = 12.2, 4.9, 2.0 Hz, 1 H, 5′-Ha), 3.65 (ddd, *J* = 12.5, 4.8, 2.8 Hz, 1 H, 5′-Hb), 3.37-3.22 (m, 2 H, 2CH_2-ethylene plus H_2O), 2.10 (s, 3 H, CH_3-Ac) ppm. ^{13}C NMR (100.6 MHz, DMSO-d_6): δ = 170.9, 169.5, 162.4, 156.4 (q, *J* = 36.0 Hz), 154.4, 144.6, 115.8 (q, *J* = 289.4 Hz), 95.1, 88.5, 83.5, 83.0, 69.2, 67.1, 58.8, 38.9, 37.0, 24.3 ppm; ES–MS calcd. for $C_{17}H_{21}F_3N_5O_8$ $[M - H]^-$ 480.13, found 480.35.

***N*⁴-Acetyl-5′-*O*-(4-methoxytrityl)-2′-*O*-[(*N*-(trifluoroacetamidoethyl)carbamoyl)methyl]cytidine (16).** Compound **15** (0.031 g, 0.064 mmol) was dried by evaporation of added anhydrous pyridine and dissolved in 1.2 mL of anhydrous DMF-DMSO-pyridine (2:1:1 v/v/v) mixture. To the resulting solution, triethylamine (0.013 mL, 0.096 mmol) was added followed by 4-methoxytritylchloride (0.039 g, 0.128 mmol) and reaction mixture was stirred at ambient temperature for 28 h. The reaction was quenched with 10% aqueous $NaHCO_3$ and mixture was extracted with ethyl acetate. The organic phase was washed with water, dried over Na_2SO_4, filtered and concentrated under reduced pressure. The residue was subjected to flash column chromatography using 0 to 8% methanol in dichloromethane as eluent to give compound **16** (0.04 g, 0.053 mmol, 83%). R_f = 0.48 (CH_2Cl_2/CH_3OH 9:1 v/v). ^1H NMR (400.1 MHz, $CDCl_3$): δ = 9.03 (s, 1 H, N*H*-Ac), 8.52 (br t, 1 H, N*H*-ethylene), 8.48 (d, *J* = 7.5 Hz, 1 H, 6-H), 8.15 (br t, 1 H, N*H*-ethylene), 7.46-7.40 (m, 4 H, trityl), 7.35-7.20 (m, 9 H, 5-H, trityl), 6.86 (d, *J* = 8.8 Hz, 2 H, trityl), 5.85 (s, 1 H, 1′-H), 5.05 (br d, 1 H, 3′-OH), 4.54-4.39 (m, 3 H, 3′-H, CH_2-carbamoyl), 4.24-4.18 (m, 1 H, 4′-H), 3.94 (d, *J* = 4.8 Hz, 1 H, 2′-H), 3.80 (s, 3 H, OCH_3-trityl), 3.64-3.47 (m, 6 H, 5′-Ha, 5′-Hb, 2CH_2-ethylene), 2.17 (s, 3 H, CH_3-Ac) ppm. ^{13}C NMR (100.6 MHz, $CDCl_3$): δ = 172.0, 170.4, 162.7, 158.8, 158.3 (q, *J* = 37.2 Hz), 156.1, 144.4, 144.0, 143.7, 134.9, 130.4, 128.4, 128.1, 127.31, 127.27, 115.8 (q, *J* = 287.5 Hz), 113.3, 97.2, 90.4, 87.3, 85.2, 82.9, 69.8, 67.8, 60.9, 55.2, 40.0, 38.0, 24.7 ppm; ES–MS calcd. for $C_{37}H_{37}F_3N_5O_9$ $[M - H]^-$ 752.25, found 752.55.

***N*⁴-Acetyl-3′-*O*-(*N*,*N*-diisopropylamino-(2-cyanoethoxy)phosphinyl)-5′-*O*-(4-methoxytrityl)-2′-*O*-[(*N*-(trifluoroacetamidoethyl)carbamoyl)methyl]cytidine (17).** To a chilled (ice bath) solution of compound **16** (2.11 g, 2.8 mmol) in 40 mL of dichloromethane-acetonitrile (5:3 v/v) mixture, *N*,*N*′-diisopropylethylamine (2.4 mL, 14 mmol) was added under nitrogen atmosphere followed by 2-cyanoethyl *N*,*N*-diisopropylphosphoramidochloridite (1.25 mL, 5.6 mmol). The reaction mixture was stirred for 30 min, allowed to warm to ambient temperature and stirred for another 2.5 h. Methanol (1.4 mL) was added and solvents were partially removed *in vacuo*. The residue was partitioned between ethyl acetate and 10% aqueous solution of $NaHCO_3$ and the aqueous phase was re-extracted with ethyl acetate. The combined organic phases were pooled and then dried over Na_2SO_4, filtered and concentrated under reduced pressure. The crude product was purified by flash column chromatography using 0 to 6% methanol in ethyl

acetate containing 0.1% of triethylamine as eluent to give **17** (2.45 g, 92%). R_f = 0.57 (CH_2Cl_2/CH_3OH 9:1 v/v). ^{31}P NMR (162.0 MHz, $CDCl_3$): δ = 152.5, 149.2 ppm; HRMS (ESI-TOF): calcd. for $C_{46}H_{54}F_3N_7O_{10}P$ $[M - H]^-$ 952.3627, found 952.3629.

Synthesis of azido functionalized peptide (ELL-peptide)

The peptide was synthesized on a Biotage® Initiator+ Alstra™ microwave peptide synthesizer using manufacturer's protocol. The sequence was assembled on Rink Amide ChemMatrix® resin (Biotage) using Fmoc chemistry under inert gas (N_2). The resin (106 mg, 50 µmol, 0.47 mmol/g) was placed in a 5 mL reactor vial and swollen for 20 min at 70 °C. The Fmoc deprotection was performed at room temperature in two stages by the treatment of the resin with 20% piperidine in NMP for 3 min followed by the second treatment for 10 min. Peptide couplings were performed using 5 equiv. of amino acid monomers (Iris Biotech GmbH), 5 equiv. oxyma and 5 equiv. DIC in NMP with coupling time of 6 min at 75 °C (microwave). Prior to coupling of 2-(2-azidoethoxy)ethoxyacetic acid (Iris Biotech GmbH) its cyclohexylamine salt was transformed into the acid form by washing through DOWEX 50WX8-200 ion-exchange resin (Sigma-Aldrich). The resin was washed with NMP followed by a capping step using NMP-lutidine-acetic anhydride (89:6:5 v/v/v) for 2 min. After completion of the synthesis the resin was extensively washed with NMP and DCM and thoroughly dried. Deprotection of the product and cleavage from solid support was achieved by the treatment with TFA-H_2O-TIS (95:2.5:2.5 v/v/v, 10 mL/g of polymer) cocktail for 3 h at room temperature. The mixture was concentrated and the crude product was precipitated by addition of cold diethyl ether, centrifuged and washed with an additional portion of diethyl ether and dried in air. The residue was re-dissolved in water and lyophilized. Crude product was purified by RP-HPLC on a Phenomenex Jupiter® 4 µm Proteo 90 Å (250 × 4.6 mm) column with 1 mL/min flow rate and linear gradient elution of 40% to 100% of solvent B in solvent A in 40 min (solvent A = 0.1% TFA in water; solvent B = 0.1% TFA in 90% aqueous acetonitrile) using detection at 280 nm and temperature 25 °C. t_R = 24.0 min. ES-MS, calc *m/z* $[M+H]^+$ 1712.89, found 1712.78.

Thermal melting analysis

Thermal melting experiments were performed on a Varian Cary 300 UV–vis dual beam spectrophotometer (Varian) equipped with a temperature controller and Varian Cary WinUV software (version 3). Melting temperature (T_m) values of the duplexes were determined from absorbance vs. temperature profiles measured at 260 nm. Samples were prepared by mixing **ON1** or **ON11-16** (as well as the native DNA with corresponding sequence, for comparison) with the complementary RNA-ON using a 1:1 molar ratio, each at a concentration of 4 µM in a 10 mM phosphate buffer containing 100 mM NaCl and 0.1 mM EDTA at pH 7.0. The samples were rapidly heated to 90 °C, left for 5 min and then allowed to cool to 20 °C. After equilibration for 10 min at the starting temperature, the dissociation was recorded by heating to 90 °C at a rate of 0.2 °C/min. Melting temperatures were determined from the maxima of the first derivative plots of absorbance versus temperature. All T_m values given are the averages of three independent sets of experiments.

Analysing splice correction by RT-PCR

HeLa705, U2OS-705 and U-2 OS-mBTKi4 cells were treated with ONs at indicated concentrations as described above. After 72 h cells were harvested, by adding 350 µL RLT-buffer from the RNeasy mini kit (Qiagen) and frozen at -80°. After thawing the lysate RNA was extracted according to the manufacturer's protocol. cDNA was prepared from 500 ng RNA using the high capacity cDNA kit from Applied Biosystems. In the 705 system the splice pattern was analyzed by PCR using primers Fw705: 5′-TTGATATGTGGATTTCGAGTCGTC-3′ and Rev705: 5′-TGTCAATCAGAGTGCTTTTGGCG-3′ (Cyber- Gene); with the PCR program 5 min at 95°C; (30 s 95°C, 30 s 55°C, 30 s 72°C) for 29 cycles and 10 min at 72°C for final extension. To analyze the splice correction for the BTK ONs a multiplex PCR was performed using primers binding in the *EGFPLuc* gene Fwd: 5′-CTGGTGCCAACCCTATTCTCCTTC, and Rev: 5′-CCAGATCCACAACCTTCGCTTCAA, together with primers for the *HPRT* gene Fwd: 5′-GACTTTGCTTTCCTTGGTCAG and Rev: 5′-GGCTTATATCCAACACTTCGTGGG as a quality control for the RNA, and a program with 27 cycles with the annealing temperature set to 57 °C. The PCRs were conducted using the HotStarTaq plus master mix (Qiagen). The PCR products were run on 2% and 1.2% agarose gels respectively, in 0.5xTBE and with SYBR-safe (Thermo Fisher Scientific) to visualize the DNA fragments. Results were detected in a VersaDoc imaging system (Bio-Rad) and analyzed using the QuantityOne software (Bio-Rad). The ratio of correctly spliced RNA fragment as percentage of the total amount of fragments from the reporter gene was then calculated for each sample.

Cytotoxic analysis with WST-1 reagent

Cells were seeded at a density of $8x10^3$ (U-2 OS_705 and Neuro-2a_705) cells per well in a 96-well plate and treated with 4 or 10 µM of the respective ON as described for the splice-switching assay. After 72 h, medium was removed and new medium supplemented with WST-1 reagent (final dilution 1:10) was added to the wells. The cells were incubated for 2 h at 37 °C, 5% CO_2 in 95% humidity, followed by absorbance measurements at 450 and 650 nm, for formazan product absorbance and reference wavelength, respectively.

Data analysis

Data sets were expressed as means ± SEM. Statistical significance was determined by one-way ANOVA followed by comparison of each treatment with the group control by Fisher's Least Significance Difference (LSD) test (Graph Pad Prism 6 Software, Graph Pad Software, Inc.). In all cases $P<0.05$ was considered significant.

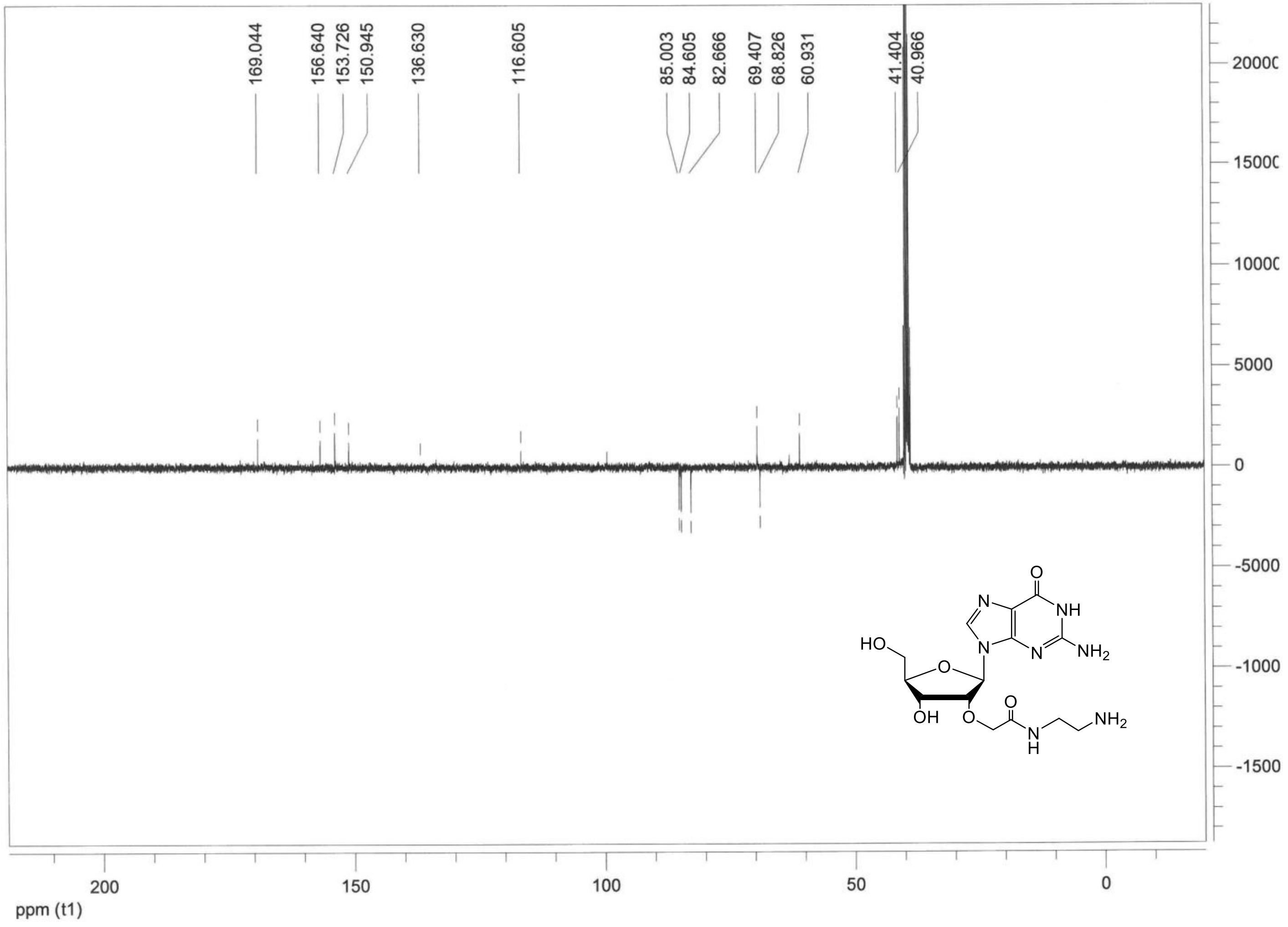

Figure S1. ^{13}C APT NMR spectrum of compound **2** (DMSO-d_6, 100.6 MHz).

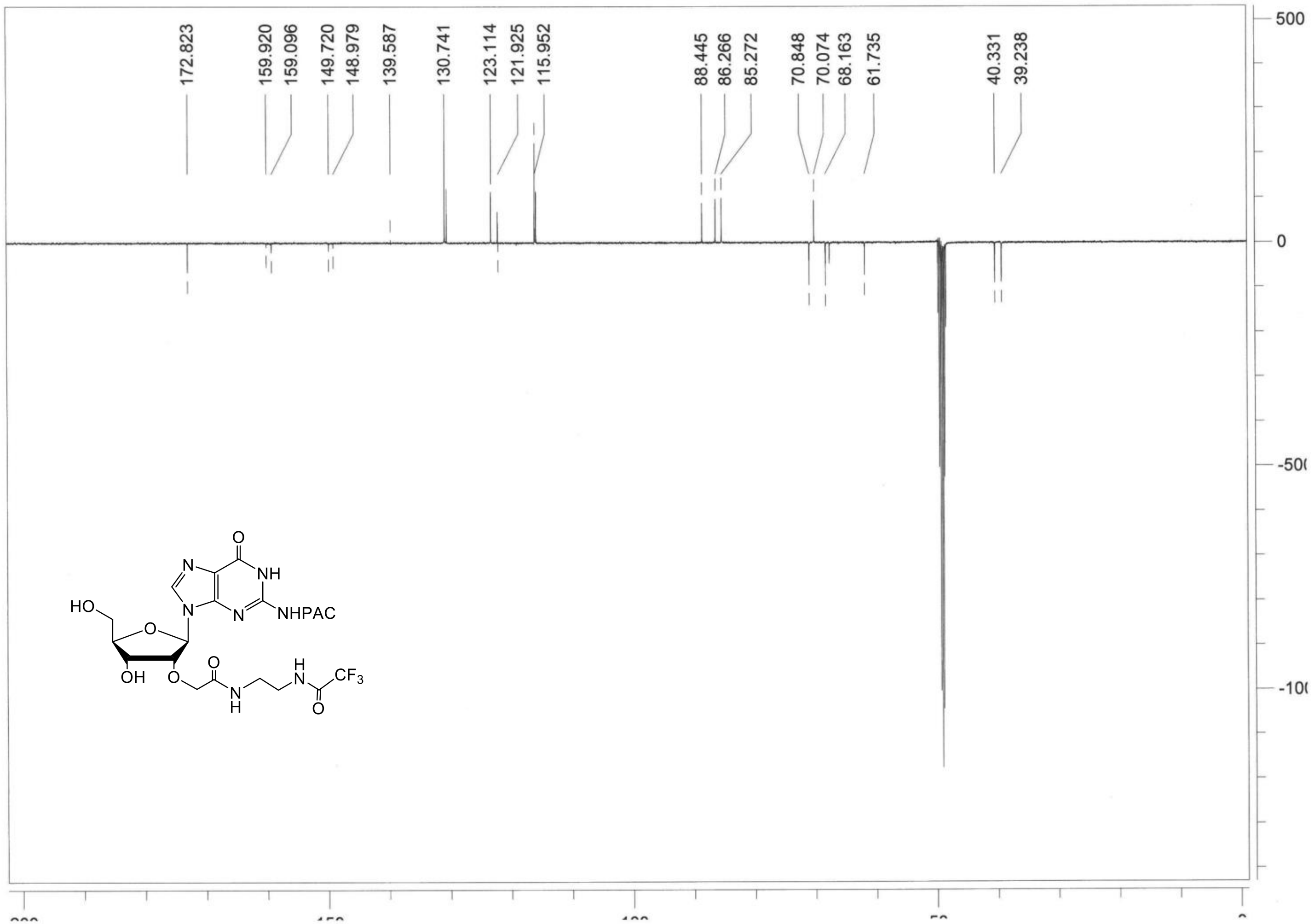

Figure S2. ^{13}C APT NMR spectrum of compound **3** (CD_3OD, 100.6 MHz).

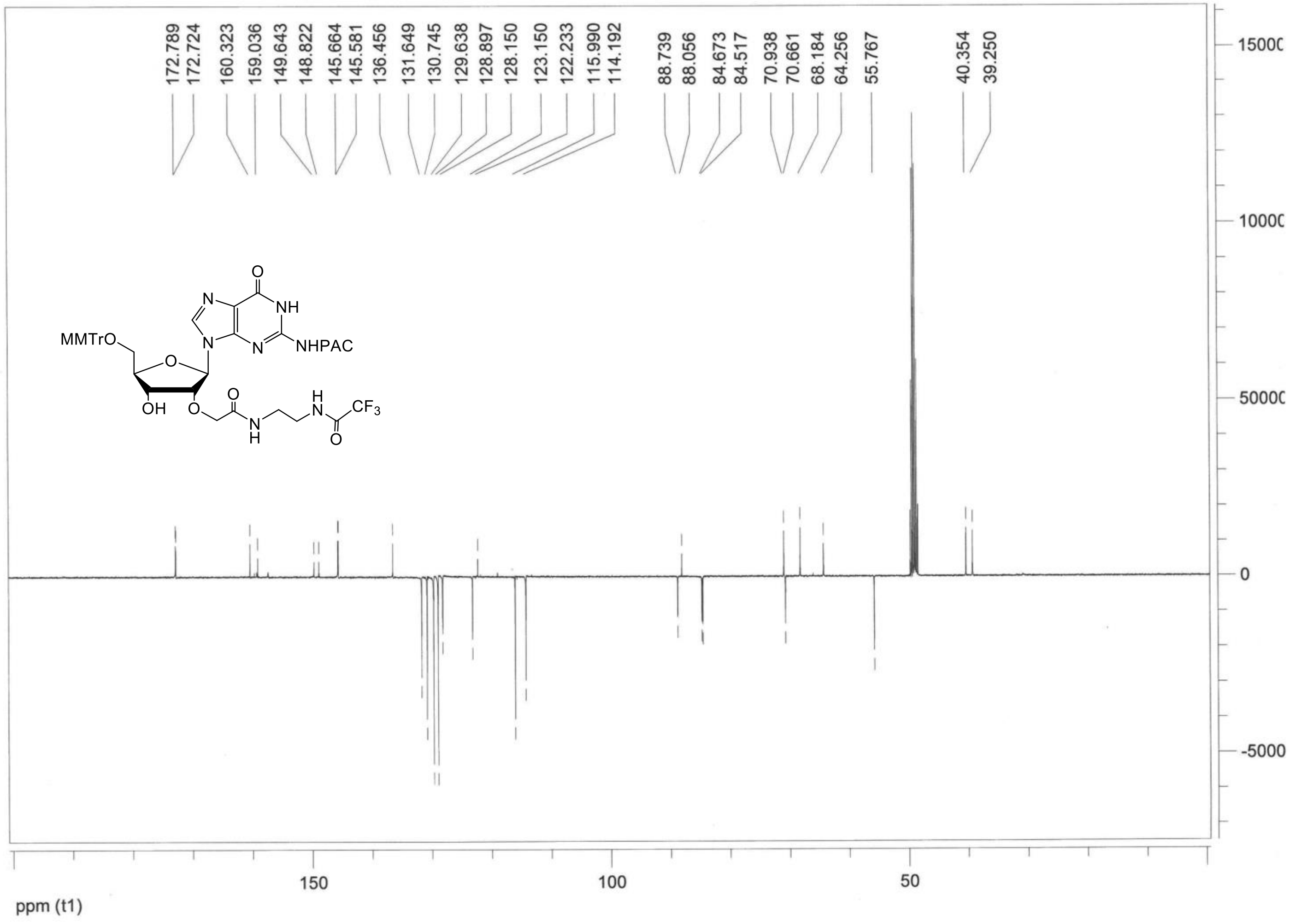

Figure S3. ^{13}C APT NMR spectrum of compound **4** (CD_3OD, 100.6 MHz).

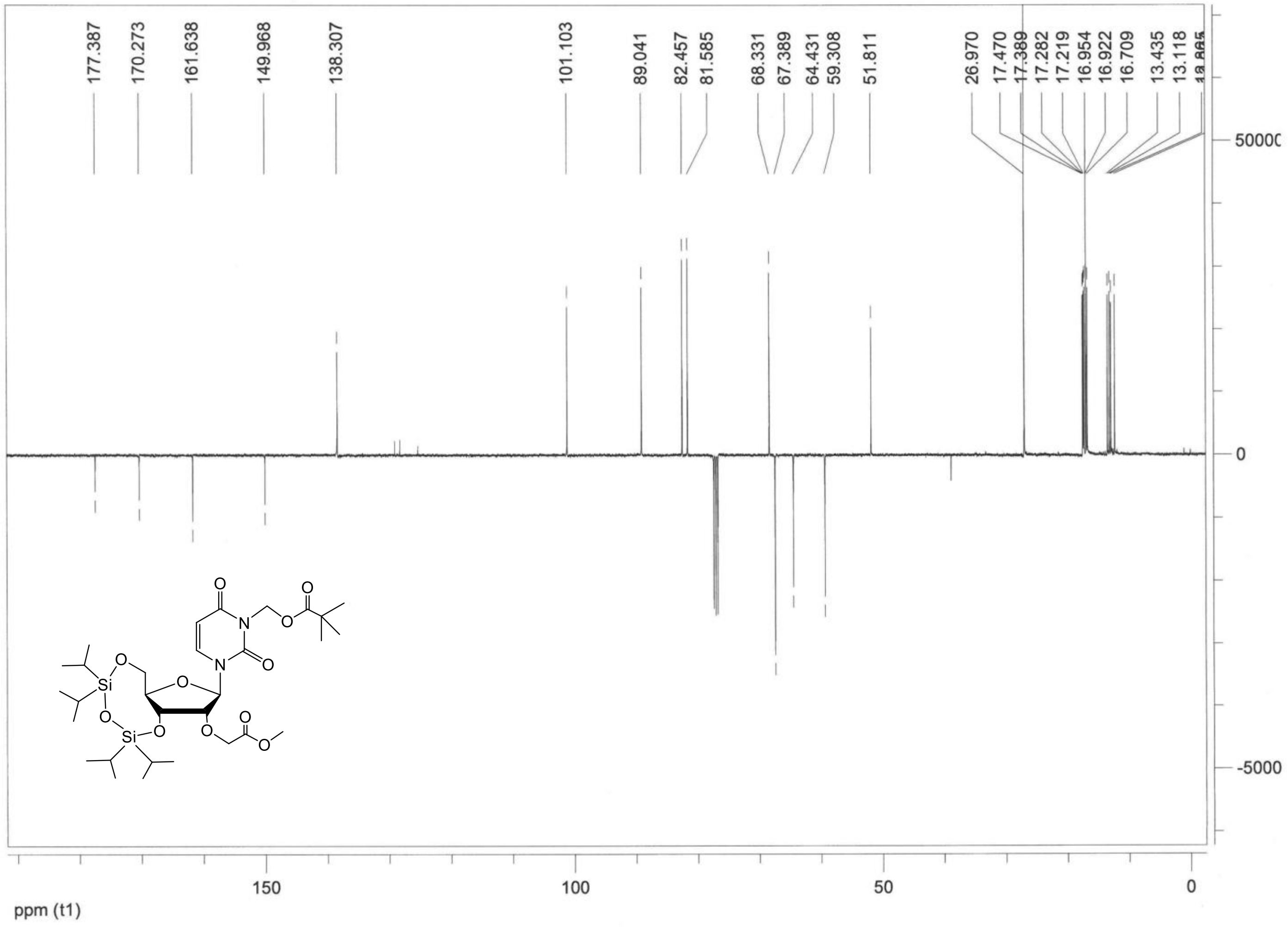

Figure S4. ^{13}C APT NMR spectrum of compound **7** ($CDCl_3$, 100.6 MHz).

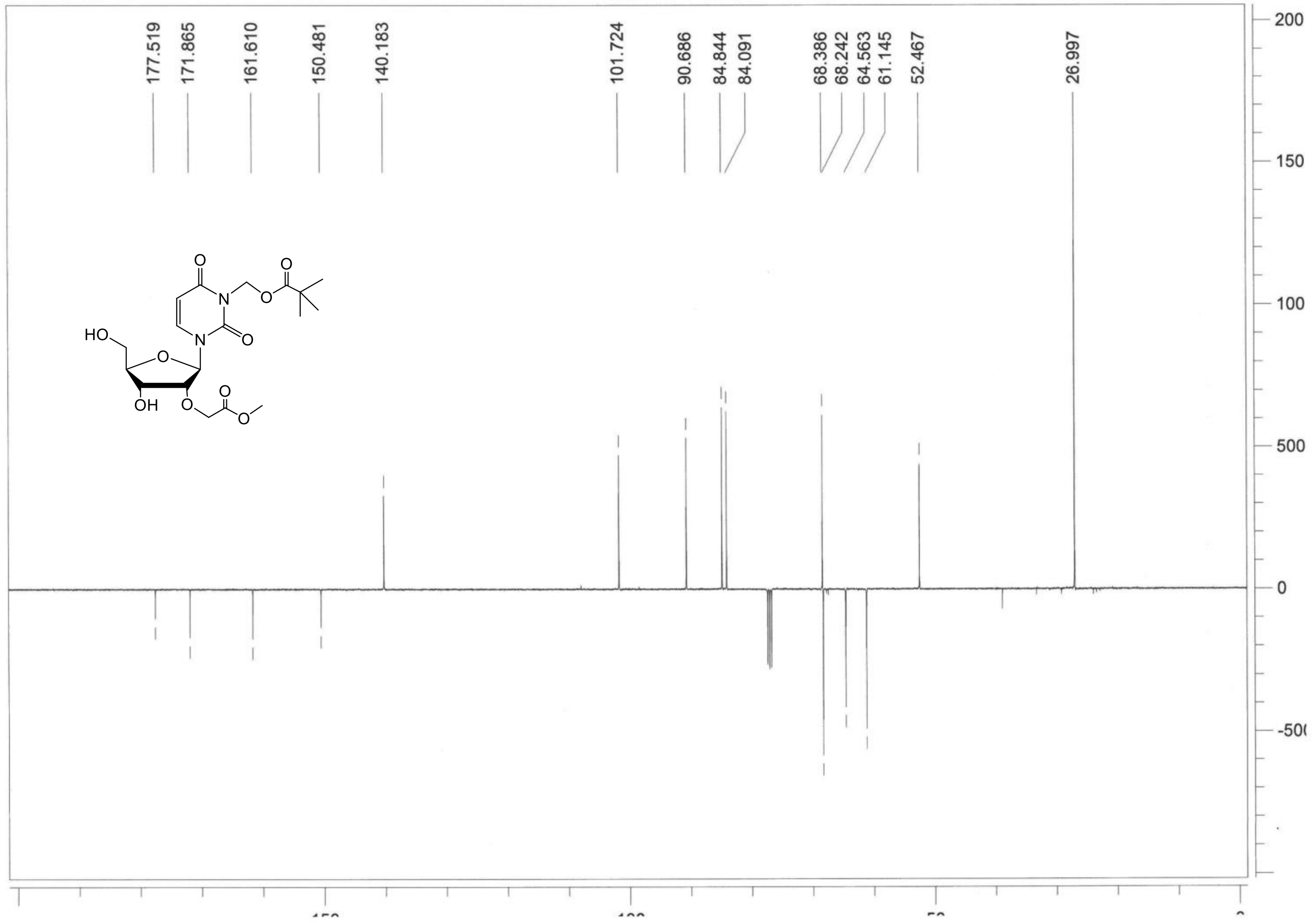

Figure S5. ^{13}C APT NMR spectrum of compound **8** ($CDCl_3$, 100.6 MHz).

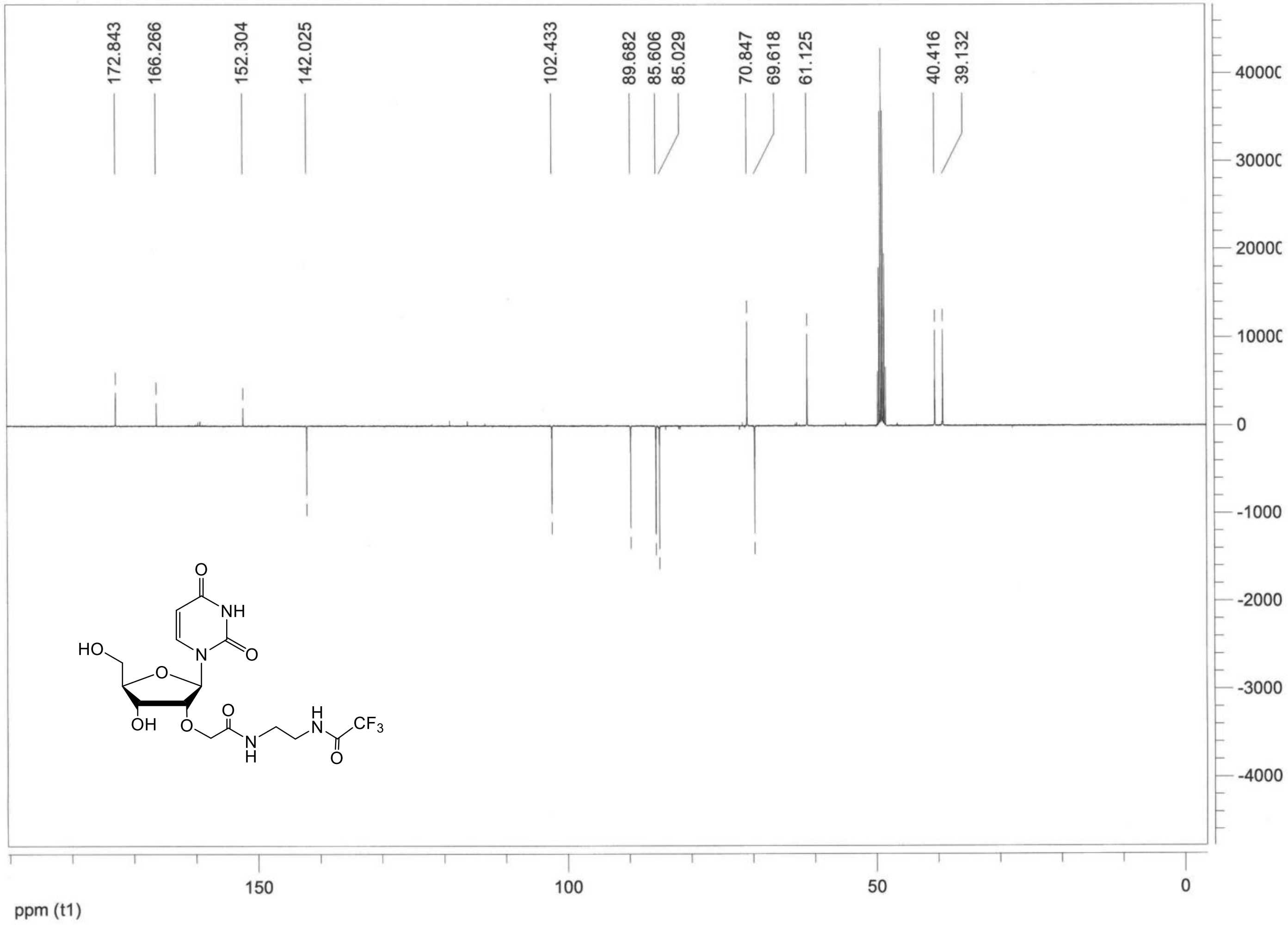

Figure S6. ^{13}C APT NMR spectrum of compound **9** (CD_3OD, 100.6 MHz).

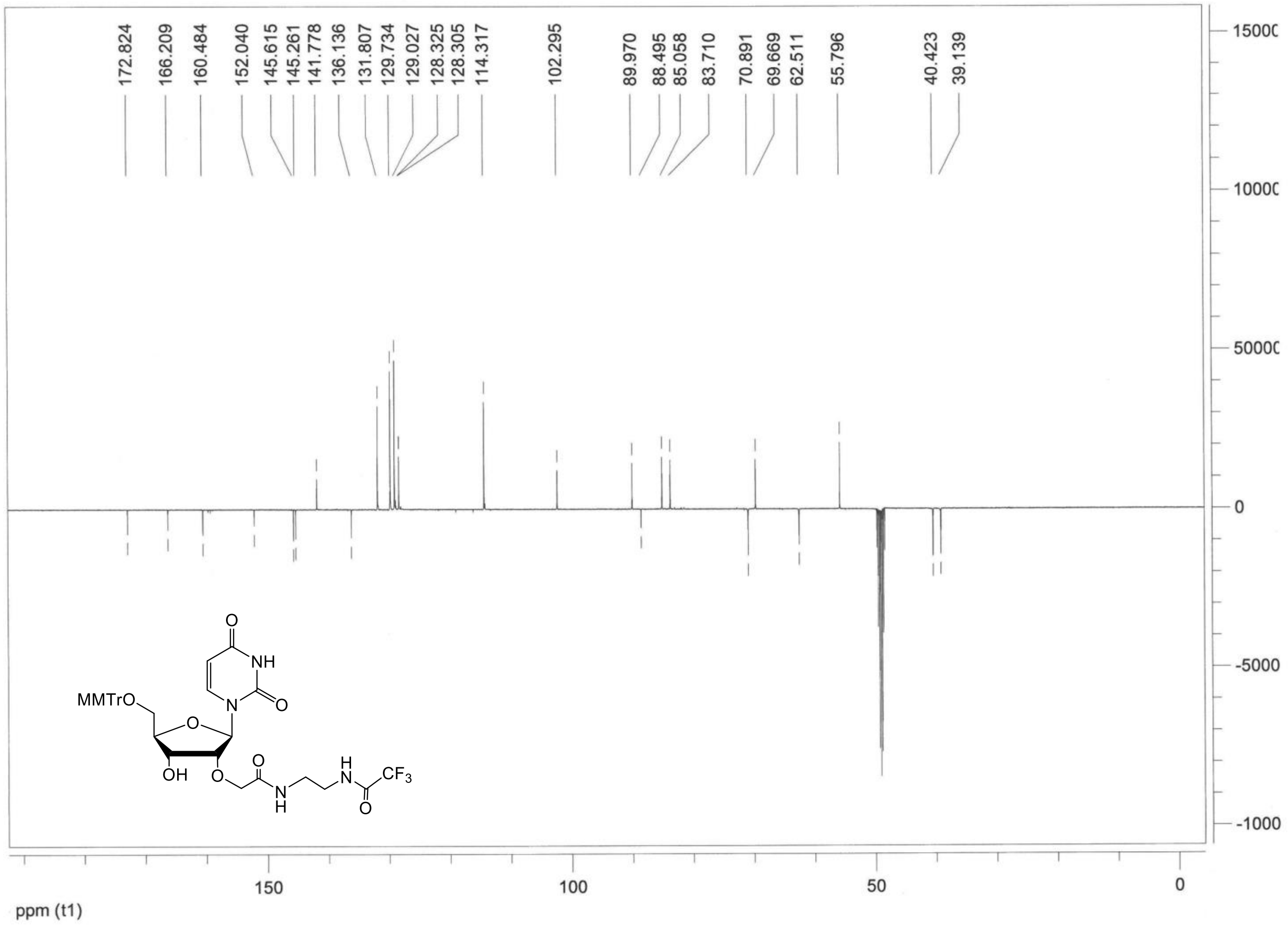

Figure S7. ^{13}C APT NMR spectrum of compound **10** (CD_3OD, 100.6 MHz).

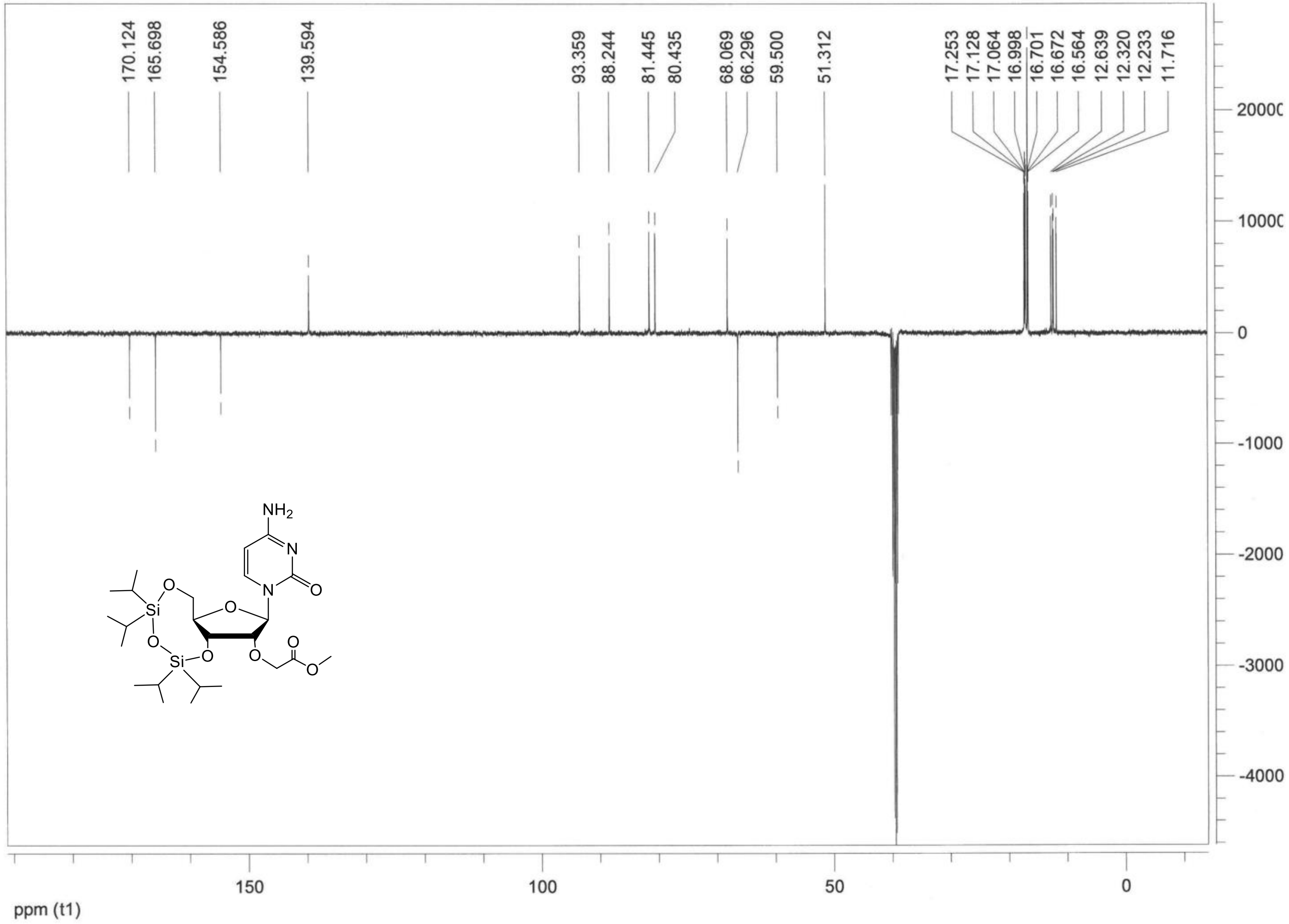

Figure S8. ^{13}C APT NMR spectrum of compound **13** (DMSO-d_6, 100.6 MHz).

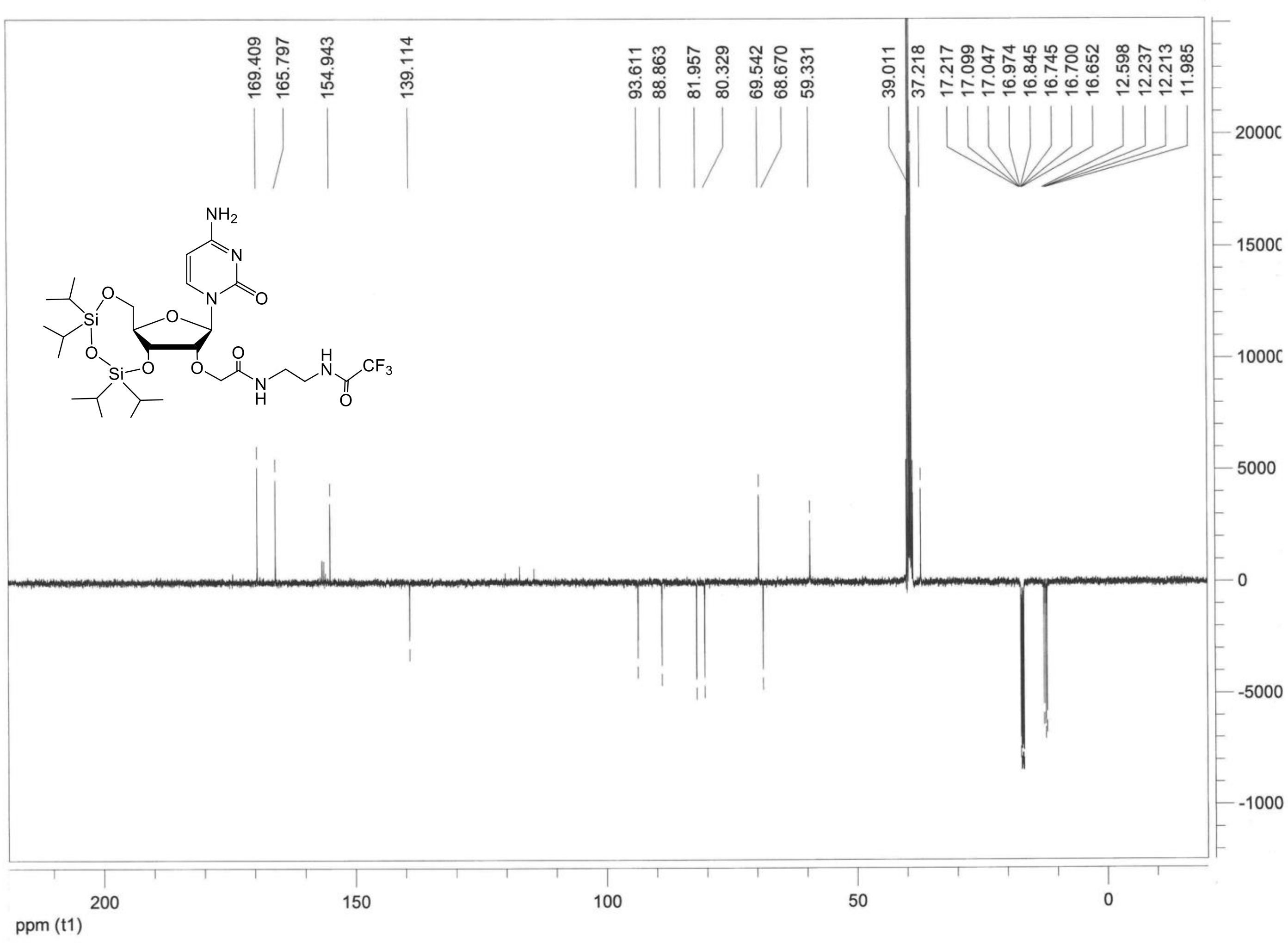

Figure S9. ^{13}C APT NMR spectrum of compound **14** (DMSO-d_6, 100.6 MHz).

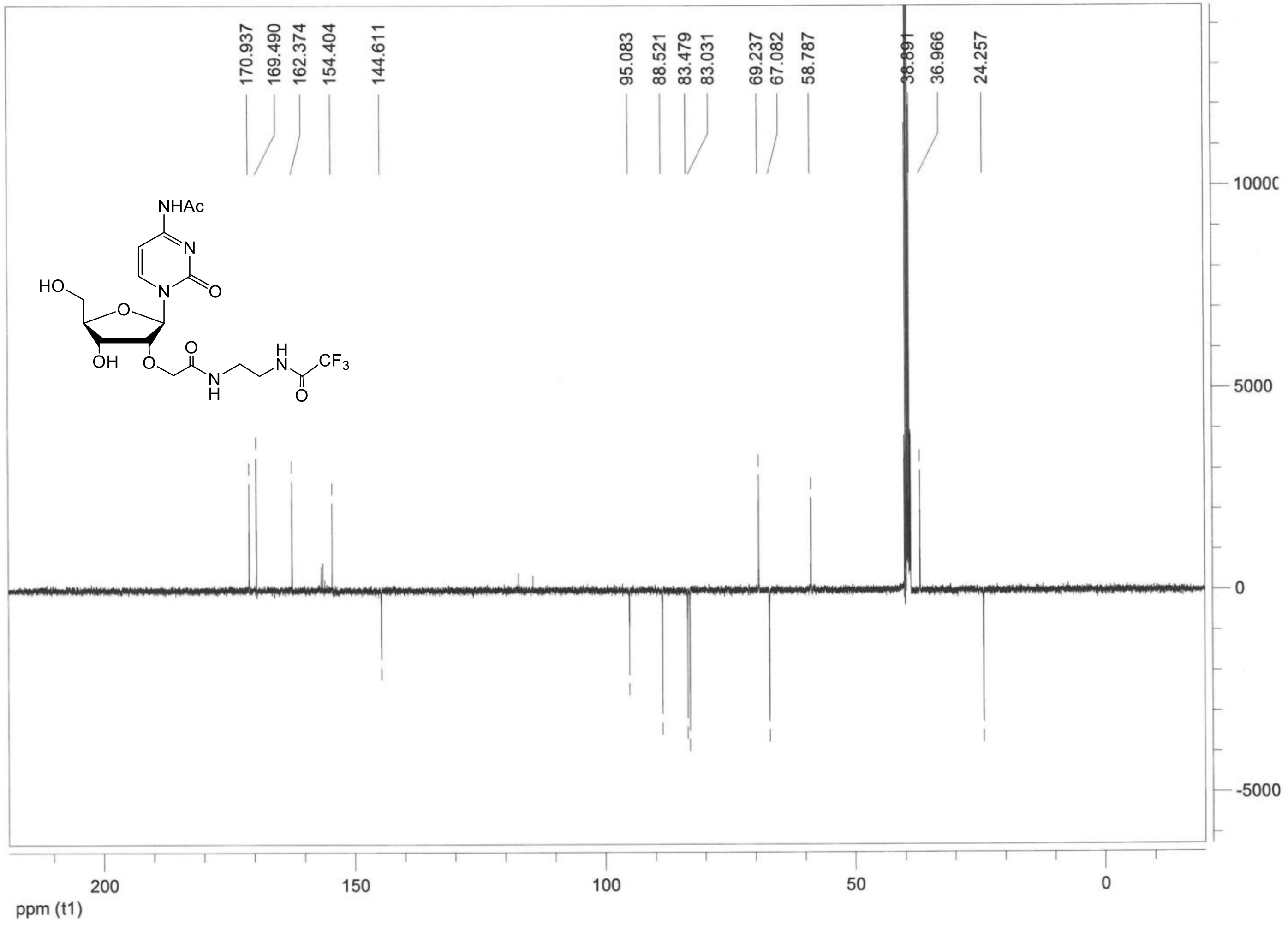

Figure S10. ^{13}C APT NMR spectrum of compound **15** (DMSO-d_6, 100.6 MHz).

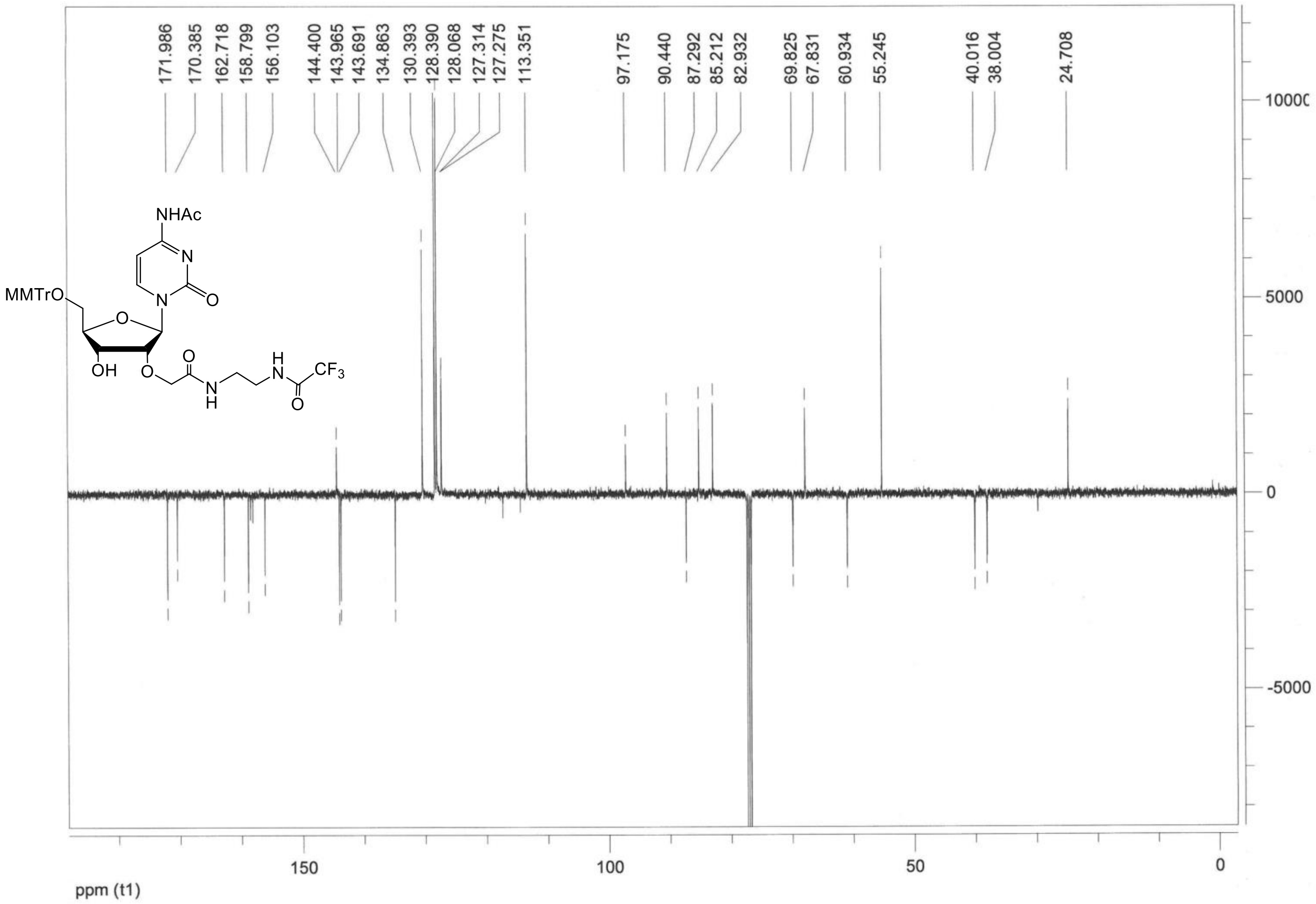

Figure S11. ^{13}C APT NMR spectrum of compound **16** ($CDCl_3$, 100.6 MHz).

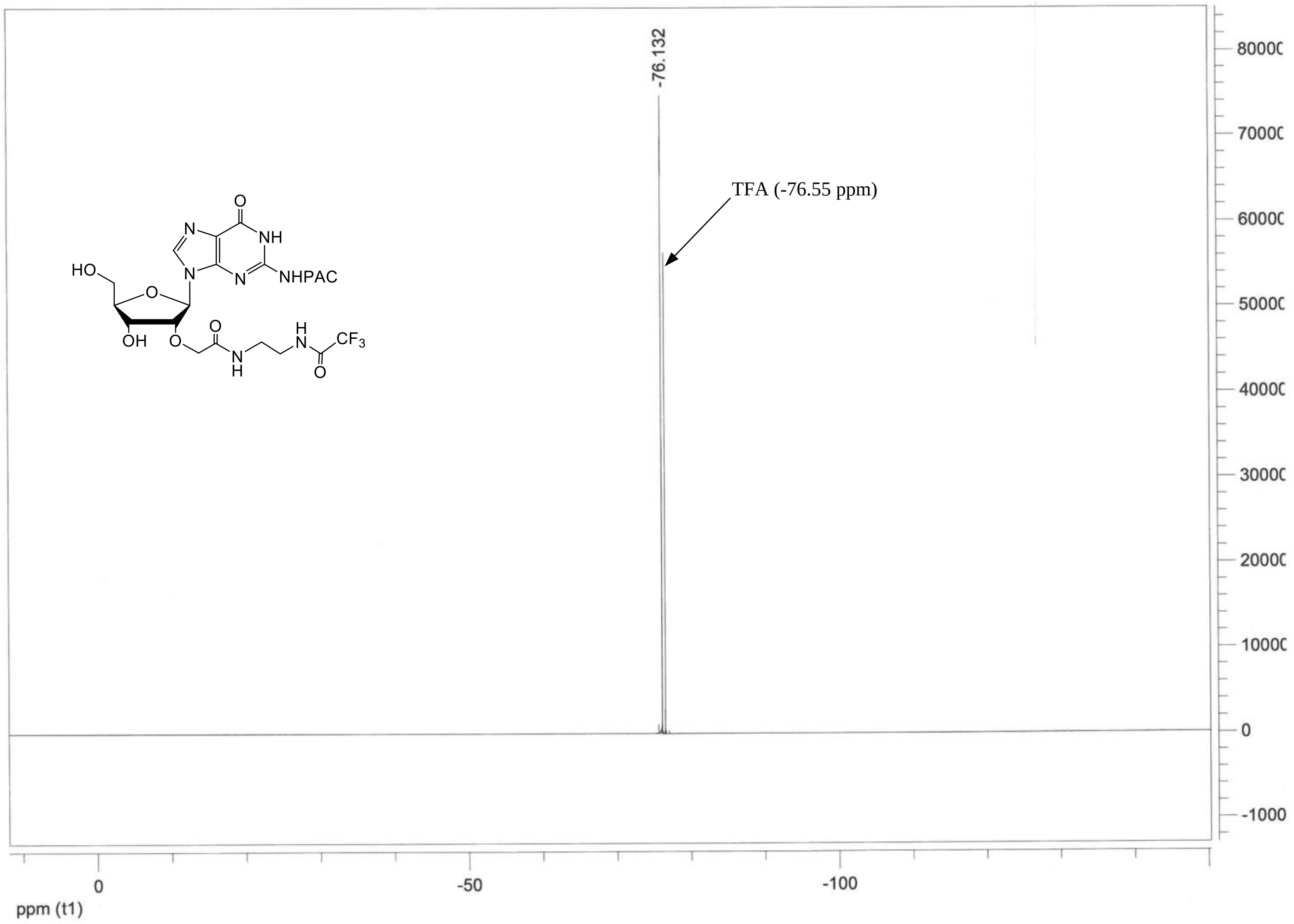

Figure S12. ^{19}F NMR spectrum of compound **3** (CD_3OD, 376.5 MHz).

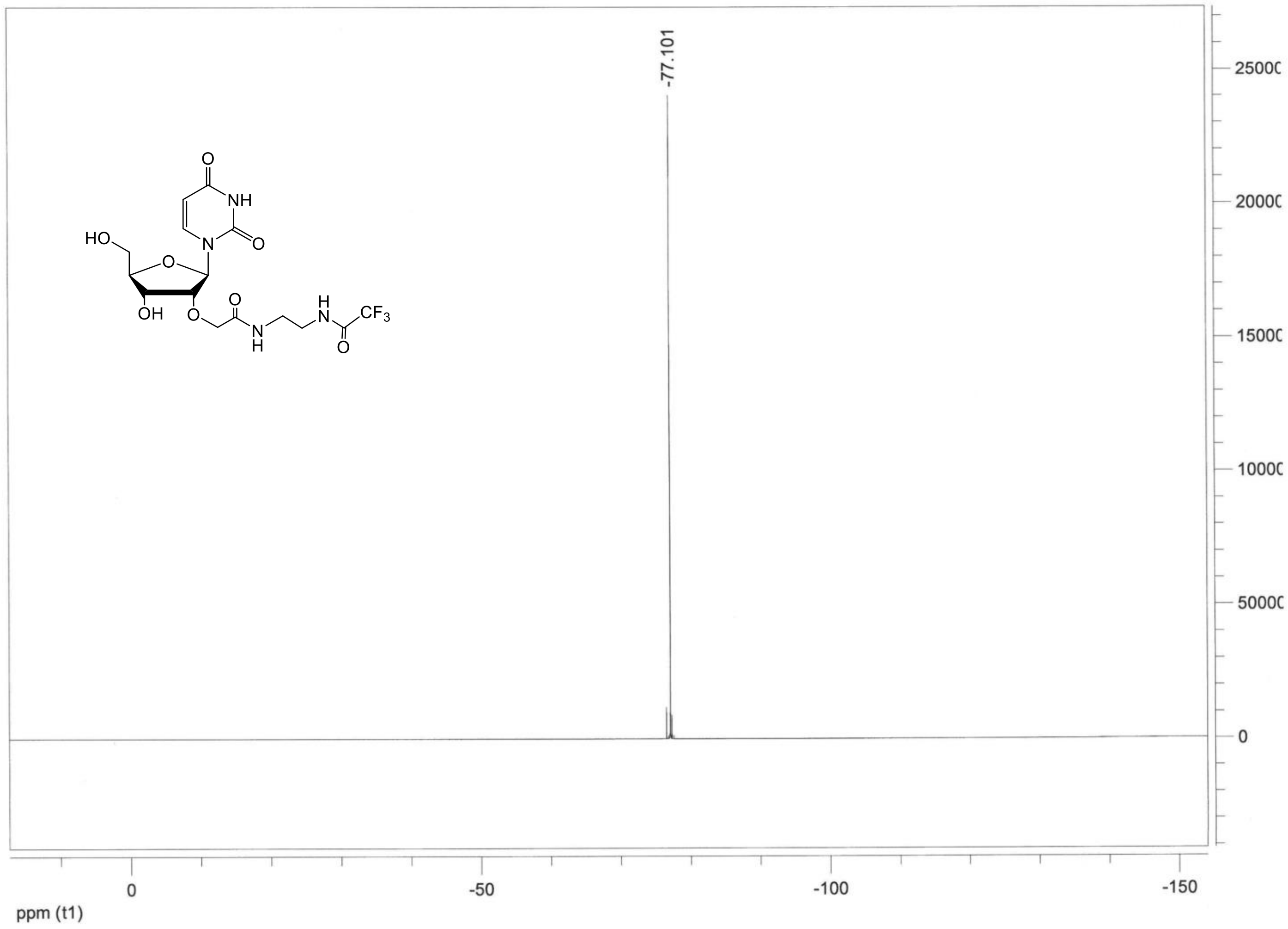

Figure S13. ^{19}F NMR spectrum of compound **9** (CD_3OD, 376.5 MHz).

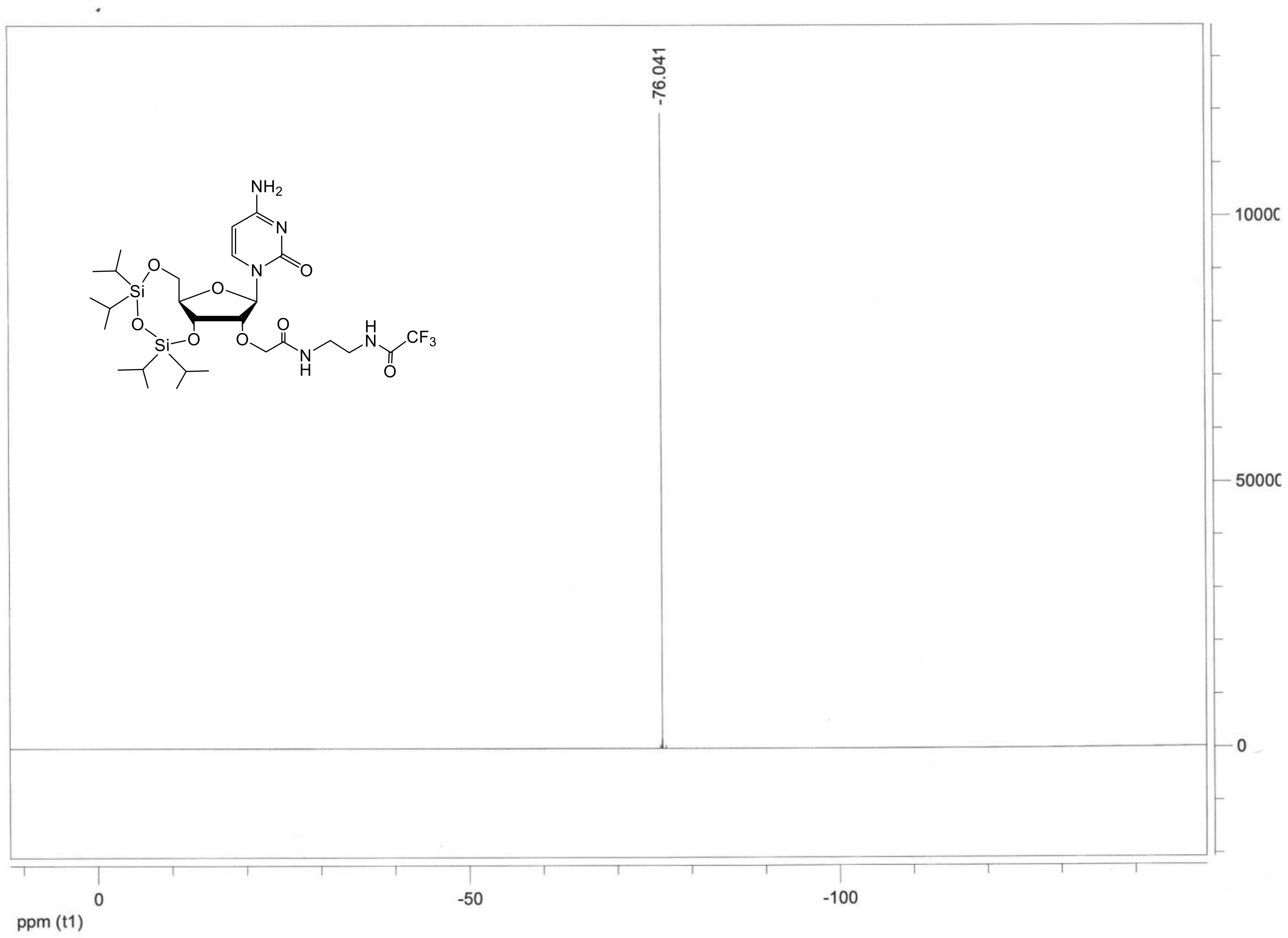

Figure S14. ^{19}F NMR spectrum of compound **14** (DMSO-d_6, 376.5 MHz).

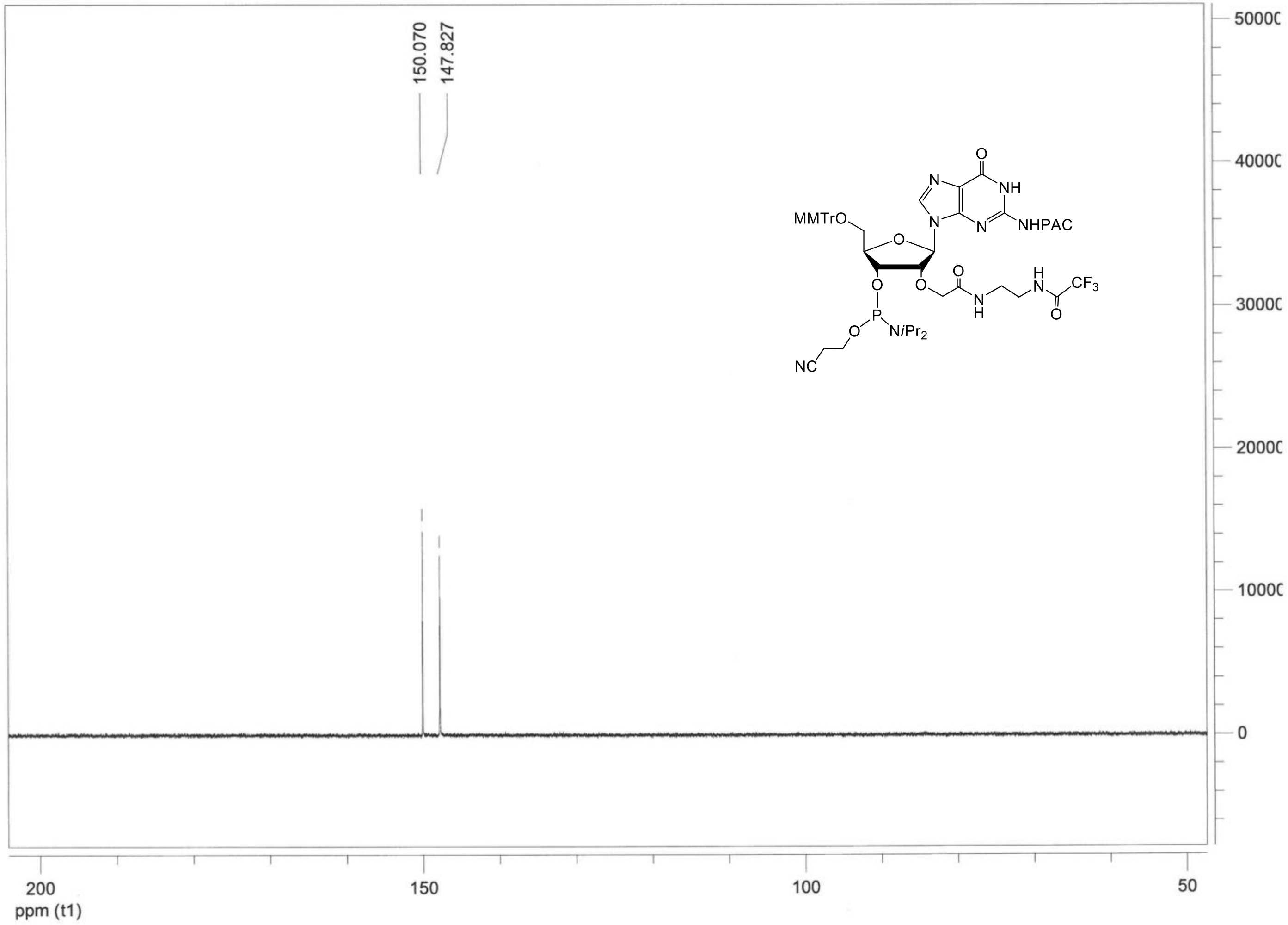

Figure S15. ^{31}P NMR spectrum of compound **5** ($CDCl_3$, 162 MHz).

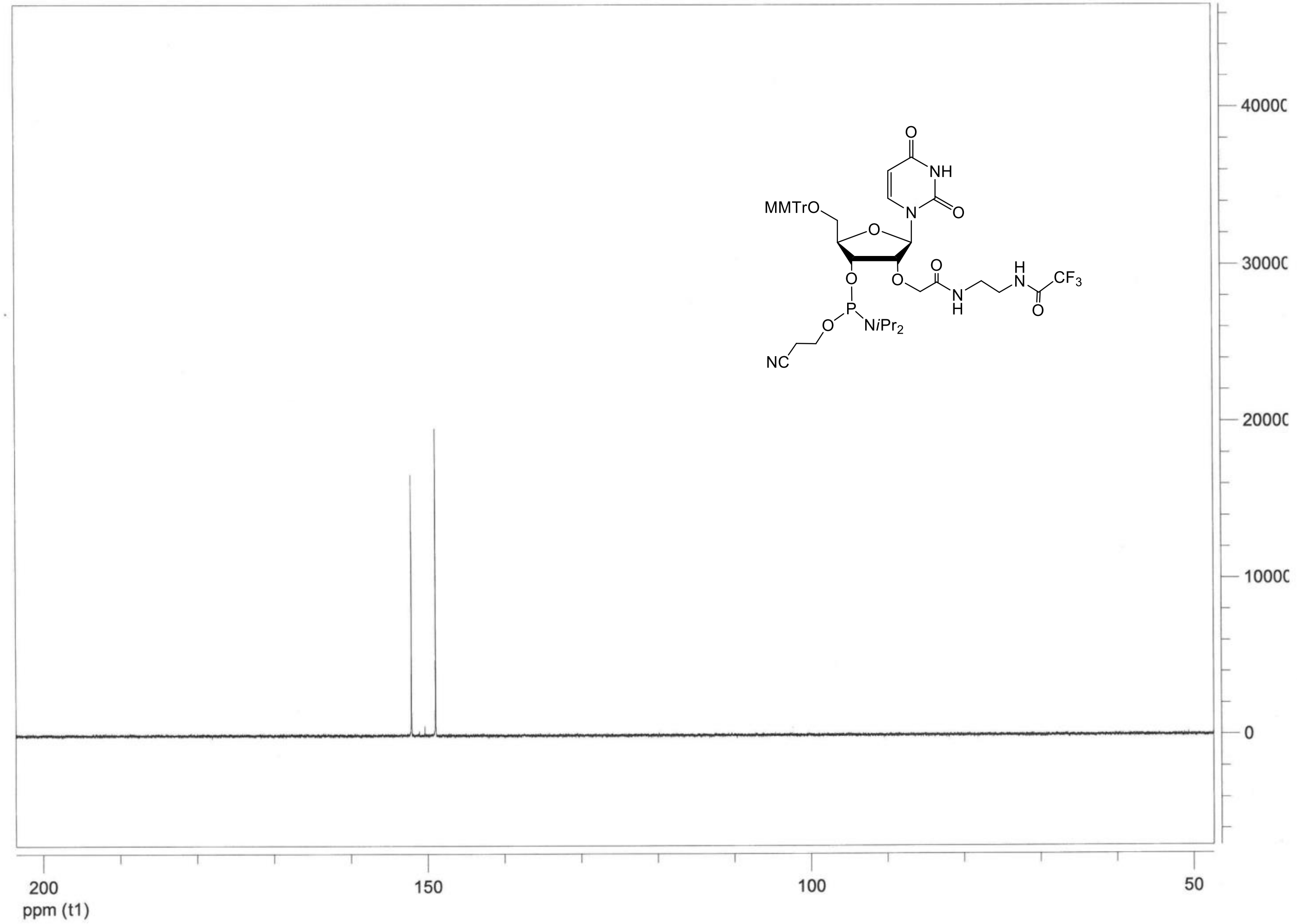

Figure S16. ^{31}P NMR spectrum of compound **11** ($CDCl_3$, 162 MHz).

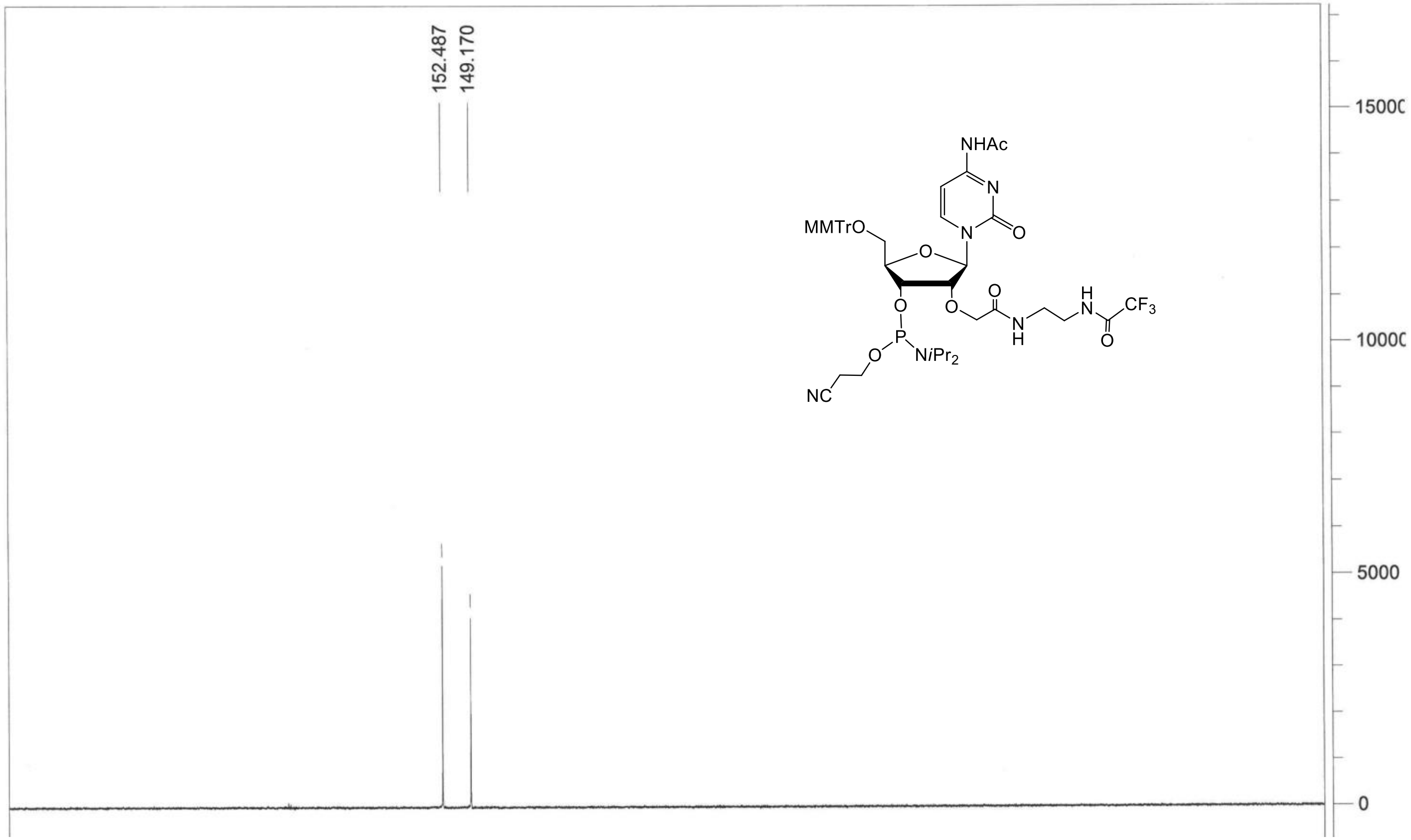

Figure S17. ^{31}P NMR spectrum of compound **17** ($CDCl_3$, 162 MHz).

Data S1. Purification details and analytical data for the synthesized ONs.

The crude deprotected **ONs1-6**, **ON12**, **ON17**, **ON19** and **ON22** were purified using RP HPLC on a Supelco Discovery® BIO Wide Pore C18-5 (250 × 10 mm) column with 4 mL/min flow rate with a linear gradient from 0 to 50% of buffer B in buffer A over 45 min at 50 °C. The oligonucleotide–ELL-peptide conjugates (**ON18**, **ON20** and **ON23**) were purified using RP HPLC on a Supelco Discovery® BIO Wide Pore C18-5 (250 × 10 mm) column with 4 mL/min flow rate with a linear gradient from 50 to 100% of buffer B in buffer A over 45 min at 25 °C. Buffer (A): 50 mM triethylammonium acetate (TEAA), pH 6.5; (buffer B): 50 mM TEAA, pH 6.5 in H_2O-CH_3CN (1:1 v/v).

The crude deprotected PS **ONs7-10** and **ONs13-15** were first purified using IE-HPLC on a Dionex, NucleoPac™ PA-100 (250 × 4 mm) column with 1.5 mL/min flow rate with a linear gradient from 0 to 40% of buffer B in buffer A over 30 min at 60 °C and then desalted by RP-HPLC using above described conditions. Buffers for IE-HPLC were as follows: (A) 20 mM sodium acetate (NaOAc) in 30% CH_3CN; (B) 20 mM NaOAc, 0.4 M lithium perchlorate ($LiClO_4$) in 30% CH_3CN.

The analytical profiles for the purified **ONs** are obtained using RP HPLC on a Supelco Discovery® BIO Wide Pore C18-5 (250 × 4.6 mm) column with 1 mL/min flow rate with a linear gradient from 0 to 100% of buffer B in buffer A over 30 min at 25 °C (a linear gradient from 0 to 50% of buffer B in buffer A over 45 min at 25 °C was used for **ON19** and **ON22**) or with a linear gradient from 50 to 100% of buffer B in buffer A over 45 min at 25 °C (for the oligonucleotide–ELL-peptide conjugates **ON18**, **ON20** and **ON23**). Buffer (A): 50 mM triethylammonium acetate (TEAA), pH 6.5; (buffer B): 50 mM TEAA, pH 6.5 in H_2O-CH_3CN (1:1 v/v).

The overall yields of 1 μmol ON syntheses based on detritylation monitoring data are approximately from 50 to 80%. The amounts and isolated yields of full length 18-mer ONs are given below (calculations are based on theoretical yield of 1 μmol ON synthesis):
ON1 (5′**-CCUCUUACCUCAGUUACA**, 18.3 OD ~ 105 nmol, 11%)

ON12 (5′-**CCUCUU**ACCUCA**GUUACA**, 28.2 OD ~ 162 nmol, 16%)

ON13 (5′-**C*C*U*C*U*U***A*C*C*U*C*A***G*U*U*A*C*A**, 15.8 OD ~ 91 nmol, 9%)

ON14 (5′-**CCUCUU**A*C*C*U*C*A***GUUACA**, 20.5 OD ~ 118 nmol, 12%)

ON15 (5′-**C*C*U*C*U*U*A*C*C*U*C*A*G*U*U*A*C*A**, 10.4 OD ~ 60 nmol, 6%)

ON17 (5′-**CCUCUUACCUCAGUUAC**A, 10.4 OD ~ 60 nmol, 6%)

ON18 (ELL-peptide-5′-**CCUCUUACCUCAGUUAC**A, 14.2 OD ~ 80 nmol, 8%)

ON19 (5′-**CAGAGUUCUCAGGAUGU**A, 50.8 OD ~ 274 nmol, 27.5%)

ON20 (ELL-peptide-5′-**CAGAGUUCUCAGGAUGU**A, 13.6 OD ~ 72 nmol, 7%)

ON22 (5′-**CCGCUGGUCCUCAGGAA**C, 27.0 OD ~ 165 nmol, 16.5%)

ON23 (ELL-peptide-5′-**CCGCUGGUCCUCAGGAA**C, 11.2 OD ~ 67 nmol, 6.7%)

The yields of Fam-containing 18-mer ONs (calculations are based on theoretical yield of 1 μmol ON synthesis):

ON2 (5′-**CCUCUUACCUCAGUUACA**-6Fam, 10.9 OD ~ 56 nmol, 6%)

ON7 (5′-C*C*U*C*U*U***ACCUCAGUUACA**-6Fam, 14.6 OD ~ 75 nmol, 7.5%)

ON8 (5′-**C*C*U*C*U*U***A*C*C*U*C*A***G*U*U*A*C*A***-6Fam, 14.1 OD ~ 72 nmol, 7%)

ON9 (5′-**C*C*U*C*U*U*A*C*C*U*C*A*G*U*U*A*C*A***-6Fam, 8.6 OD ~ 44 nmol, 4.5%)

ON10 (5′-C*C*U*C*U*U*A*C*C*U*C*A*G*U*U*A*C*A*-6Fam, 21.1 OD ~ 108 nmol, 11%)

In all sequences **A** = 2′-*O*-AECM-adenosine, **G** = 2′-*O*-AECM-guanosine, **C** = 2′-*O*-AECM-cytidine, **U** = 2′-*O*-AECM-uridine, A = 2′-OMe-adenosine, G = 2′-OMe-guanosine, C = 2′-OMe-cytidine, U = 2′-OMe-uridine, 6Fam = 6-fluorescein, * = PS linkages.

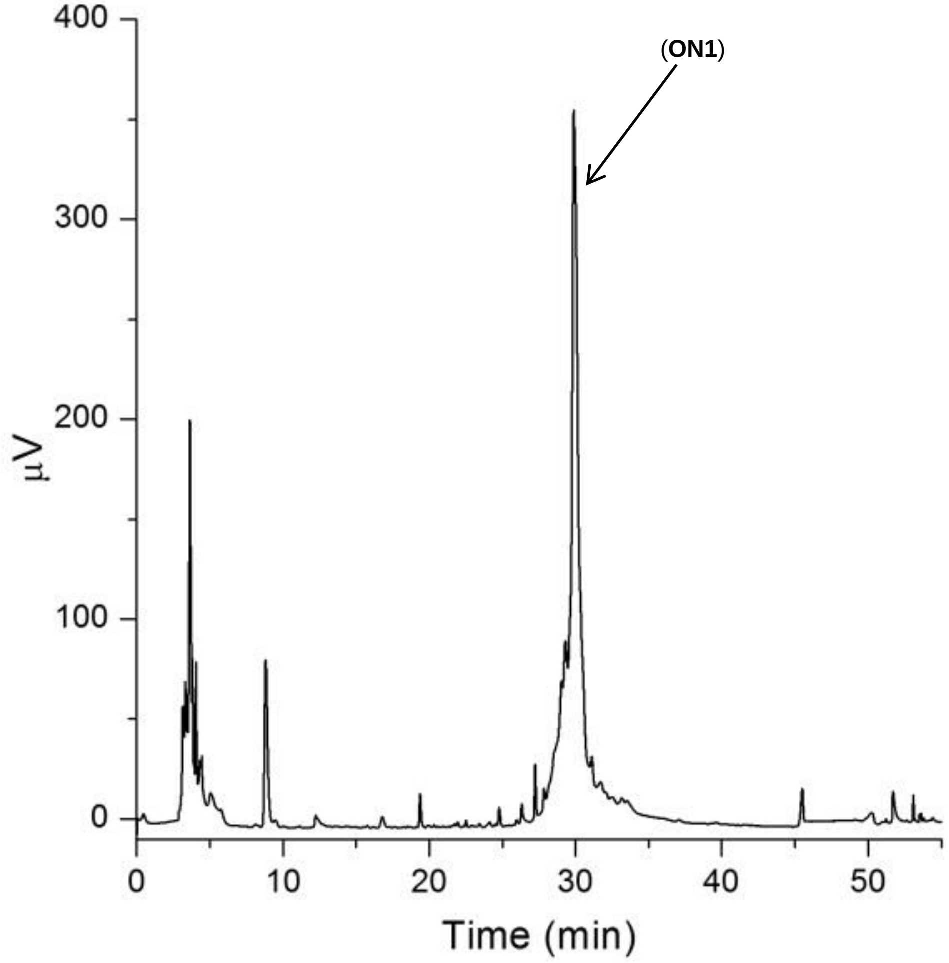

Figure S18. RP-HPLC profile of crude 18-mer 2′-*O*-AECM-modified **ON1** on a Supelco Discovery® BIO Wide Pore C18-5 column with a linear gradient from 0 to 50% of buffer B in buffer A over 45 min at 50 °C. t_R = 29.8 min. Buffer (A): 50 mM triethylammonium acetate (TEAA), pH 6.5; (buffer B): 50 mM TEAA, pH 6.5 in H_2O-CH_3CN (1:1 v/v).

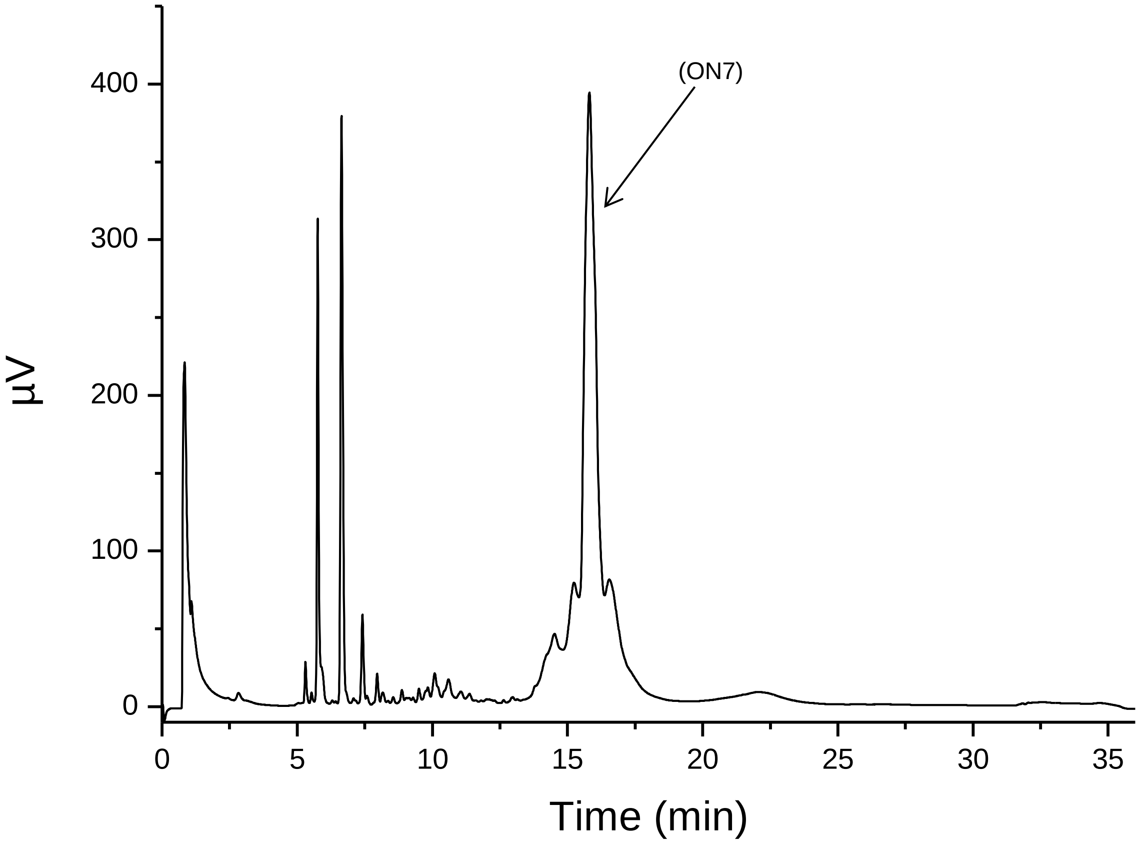

Figure S19. IE-HPLC profile of crude **ON7** on a Dionex, NucleoPac™ PA-100 (250 × 4 mm) column with 1.5 mL/min flow rate with a linear gradient from 0 to 40% of buffer B in buffer A over 30 min at 60 °C. t_R = 15.8 min. Buffer (A): 20 mM sodium acetate (NaOAc) in 30% CH_3CN; buffer (B): 20 mM NaOAc, 0.4 M lithium perchlorate ($LiClO_4$) in 30% CH_3CN.

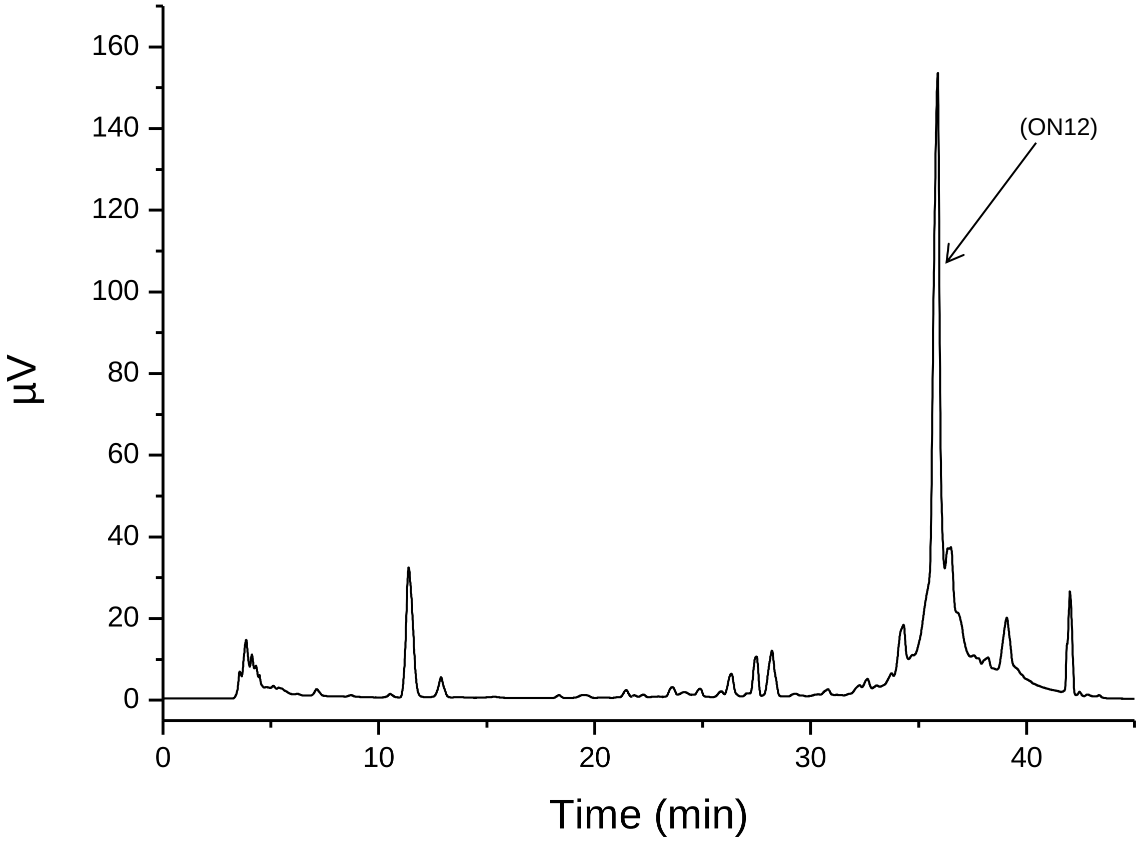

Figure S20. RP-HPLC profile of crude **ON12** on a Supelco Discovery® BIO Wide Pore C18-5 column with a linear gradient from 0 to 50% of buffer B in buffer A over 45 min at 50 °C. t_R = 35.9 min. Buffer (A): 50 mM triethylammonium acetate (TEAA), pH 6.5; (buffer B): 50 mM TEAA, pH 6.5 in H_2O-CH_3CN (1:1 v/v).

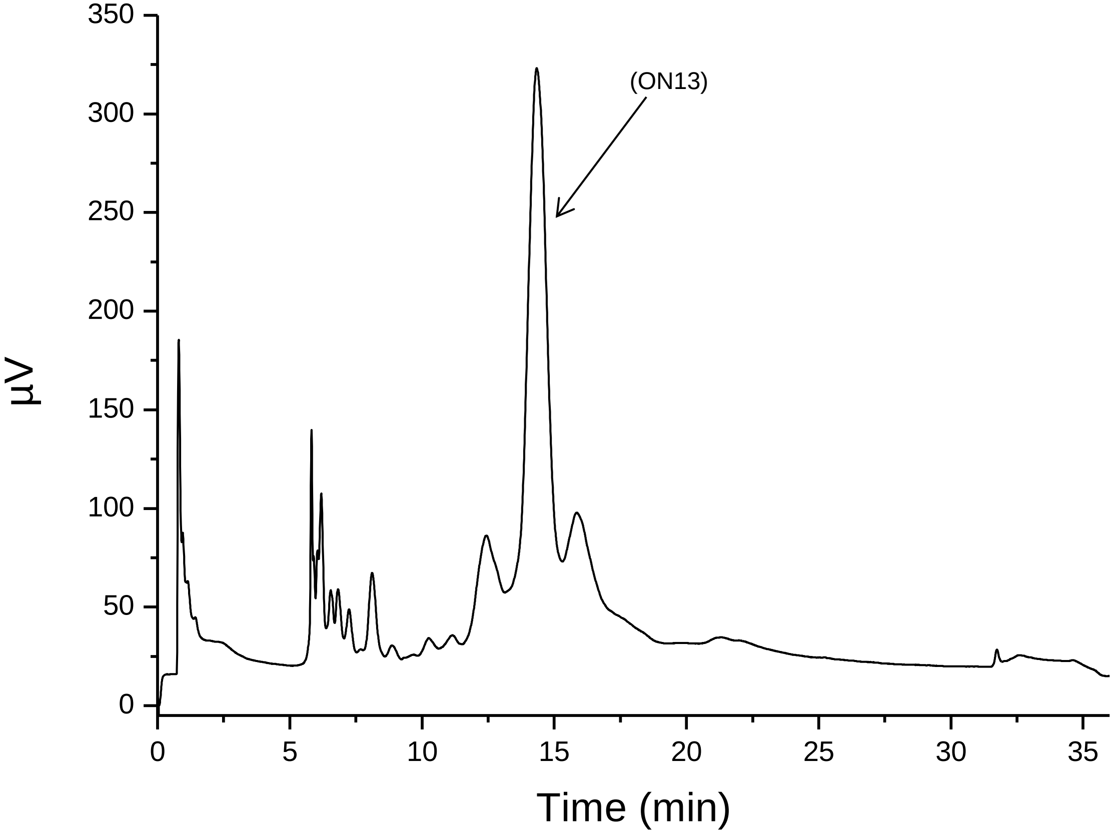

Figure S21. IE-HPLC profile of crude **ON13** on a Dionex, NucleoPac^{TM} PA-100 (250 × 4 mm) column with 1.5 mL/min flow rate with a linear gradient from 0 to 40% of buffer B in buffer A over 30 min at 60 °C. t_R = 14.3 min. Buffer (A): 20 mM sodium acetate (NaOAc) in 30% CH_3CN; buffer (B): 20 mM NaOAc, 0.4 M lithium perchlorate ($LiClO_4$) in 30% CH_3CN.

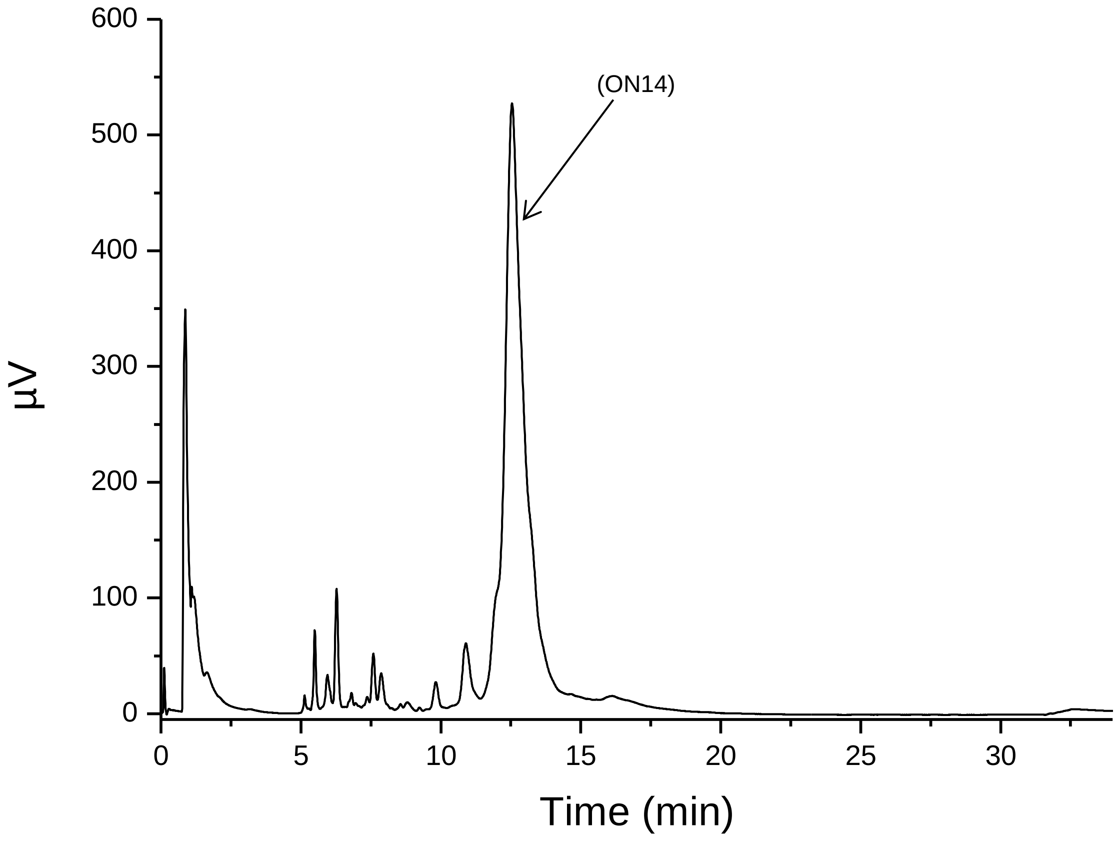

Figure S22. IE-HPLC profile of crude **ON14** on a Dionex, NucleoPac™ PA-100 (250 × 4 mm) column with 1.5 mL/min flow rate with a linear gradient from 0 to 40% of buffer B in buffer A over 30 min at 60 °C. t_R = 12.5 min. Buffer (A): 20 mM sodium acetate (NaOAc) in 30% CH_3CN; buffer (B): 20 mM NaOAc, 0.4 M lithium perchlorate ($LiClO_4$) in 30% CH_3CN.

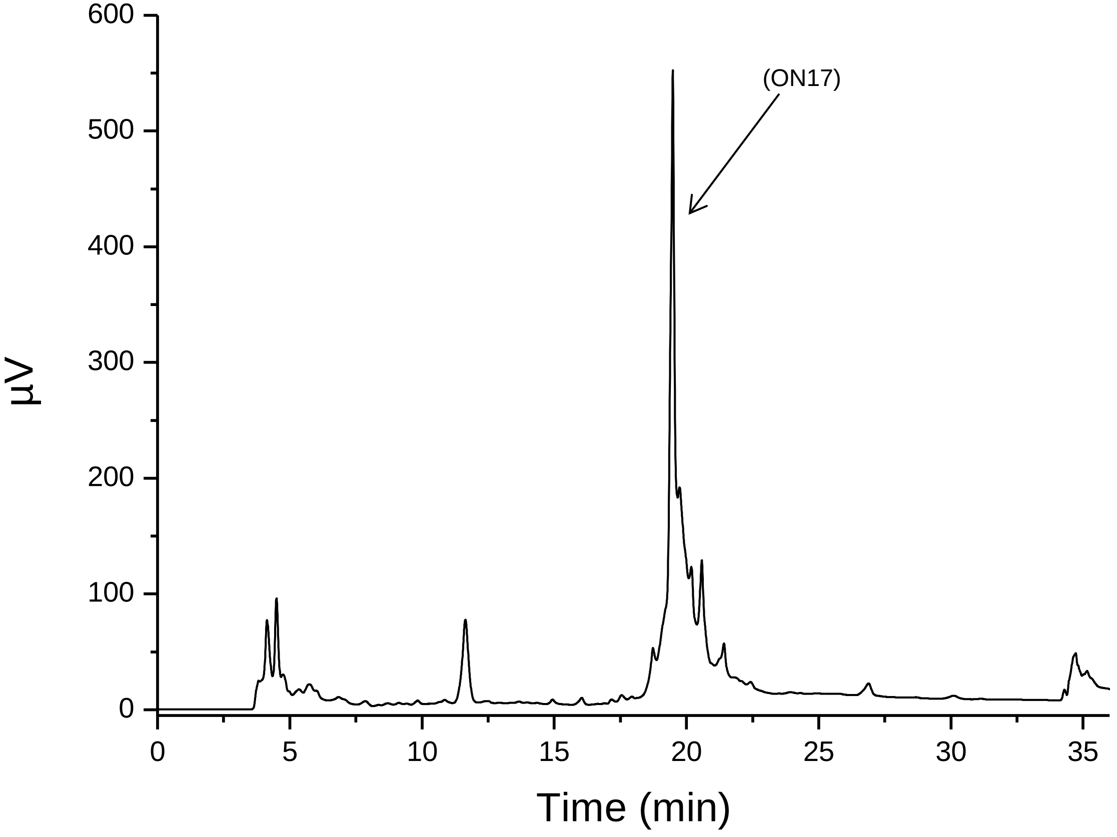

Figure S23. RP-HPLC profile of crude **ON17** on a Supelco Discovery® BIO Wide Pore C18-5 column with a linear gradient from 0 to 50% of buffer B in buffer A over 45 min at 50 °C. t_R = 19.5 min. Buffer (A): 50 mM triethylammonium acetate (TEAA), pH 6.5; (buffer B): 50 mM TEAA, pH 6.5 in H_2O-CH_3CN (1:1 v/v).

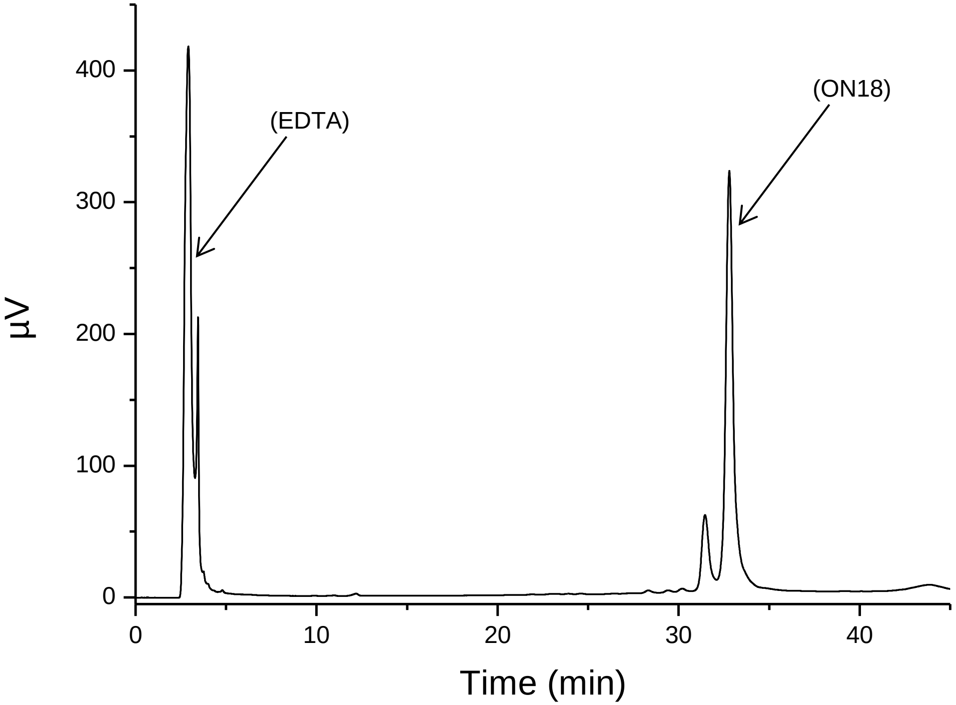

Figure S24. RP-HPLC profile from purification of crude **ON18** on a Supelco Discovery® BIO Wide Pore C18-5 (250 × 10 mm) column with a linear gradient from 50 to 100% of buffer B in buffer A over 45 min at 25 °C. t_R = 32.8 min. Buffer (A): 50 mM triethylammonium acetate (TEAA), pH 6.5; (buffer B): 50 mM TEAA, pH 6.5 in H_2O-CH_3CN (1:1 v/v).

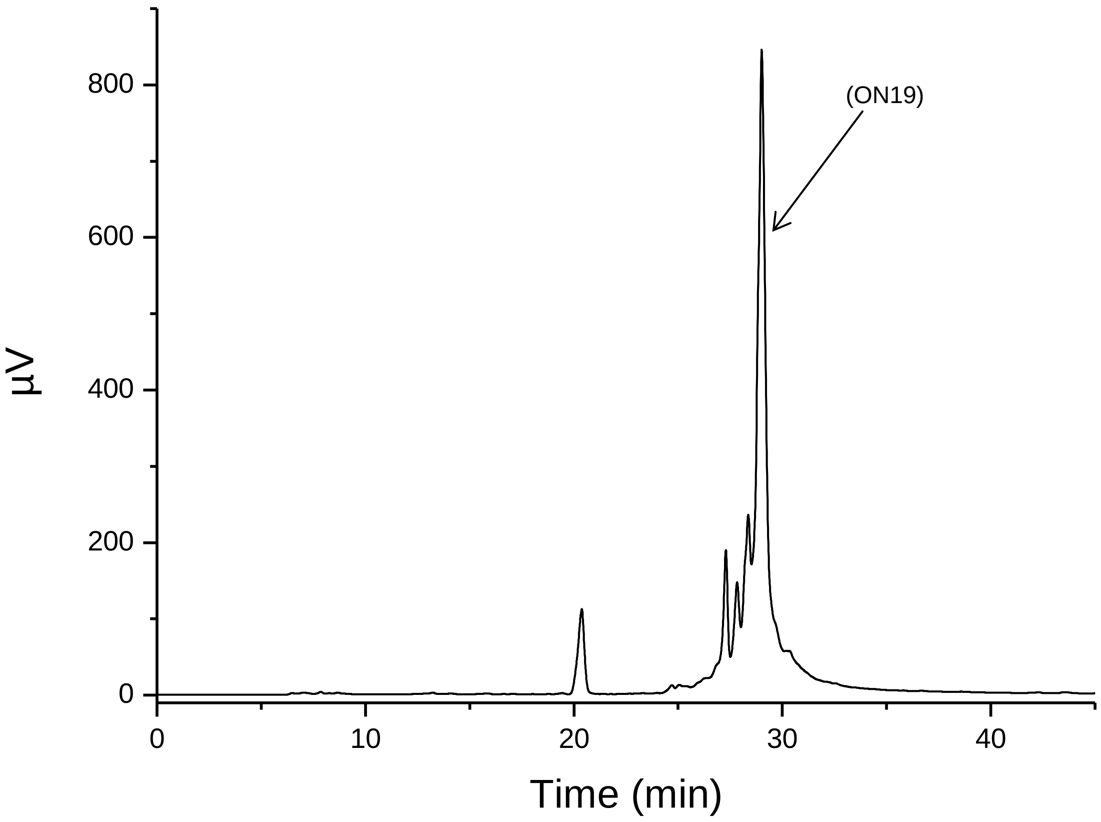

Figure S25. RP-HPLC profile of crude **ON19** on a Supelco Discovery® BIO Wide Pore C18-5 (250 × 10 mm) column with a linear gradient from 0 to 50% of buffer B in buffer A over 45 min at 25 °C. t_R = 29.0 min. Buffer (A): 50 mM triethylammonium acetate (TEAA), pH 6.5; (buffer B): 50 mM TEAA, pH 6.5 in H_2O-CH_3CN (1:1 v/v).

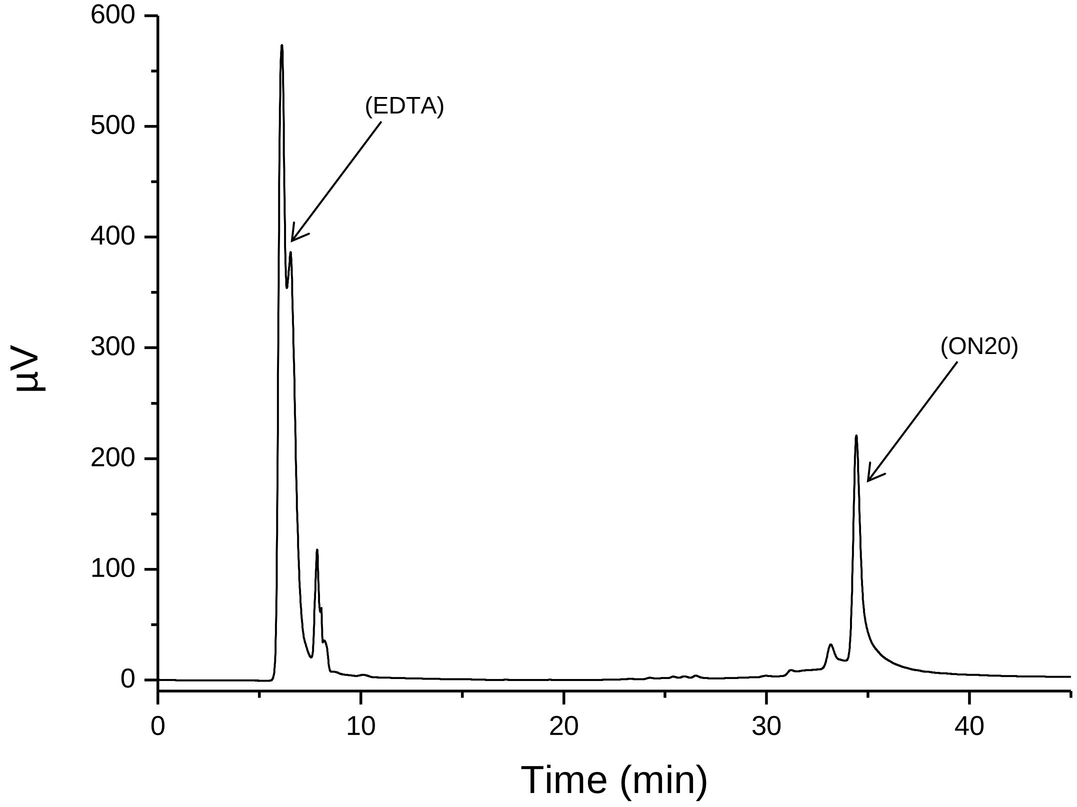

Figure S26. RP-HPLC profile of crude **ON20** on a Supelco Discovery® BIO Wide Pore C18-5 (250 × 10 mm) column with a linear gradient from 50 to 100% of buffer B in buffer A over 45 min at 25 °C. t_R = 34.4 min. Buffer (A): 50 mM triethylammonium acetate (TEAA), pH 6.5; (buffer B): 50 mM TEAA, pH 6.5 in H_2O-CH_3CN (1:1 v/v).

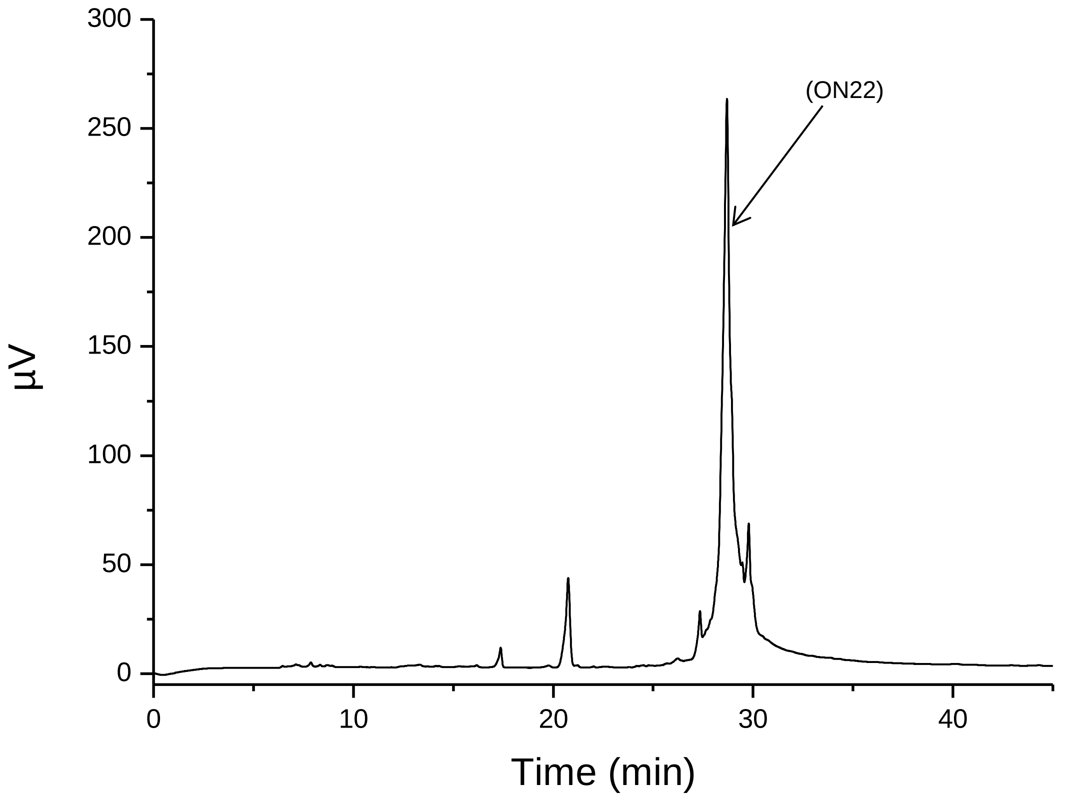

Figure S27. RP-HPLC profile of crude **ON22** on a Supelco Discovery® BIO Wide Pore C18-5 (250 × 10 mm) column with a linear gradient from 0 to 50% of buffer B in buffer A over 45 min at 25 °C. t_R = 28.7 min. Buffer (A): 50 mM triethylammonium acetate (TEAA), pH 6.5; (buffer B): 50 mM TEAA, pH 6.5 in H_2O-CH_3CN (1:1 v/v).

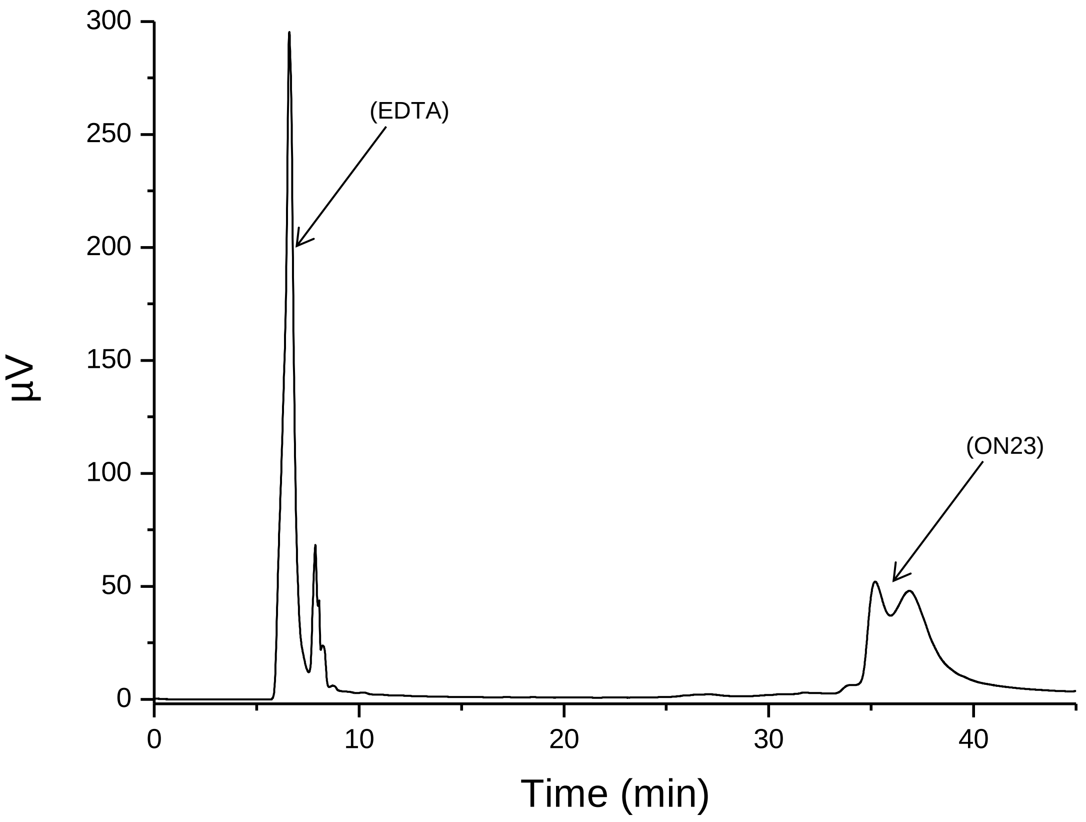

Figure S28. RP-HPLC profile of crude **ON23** on a Supelco Discovery® BIO Wide Pore C18-5 (250 × 10 mm) column with a linear gradient from 50 to 100% of buffer B in buffer A over 45 min at 25 °C. t_R = 36.0 min. Buffer (A): 50 mM triethylammonium acetate (TEAA), pH 6.5; (buffer B): 50 mM TEAA, pH 6.5 in H_2O-CH_3CN (1:1 v/v).

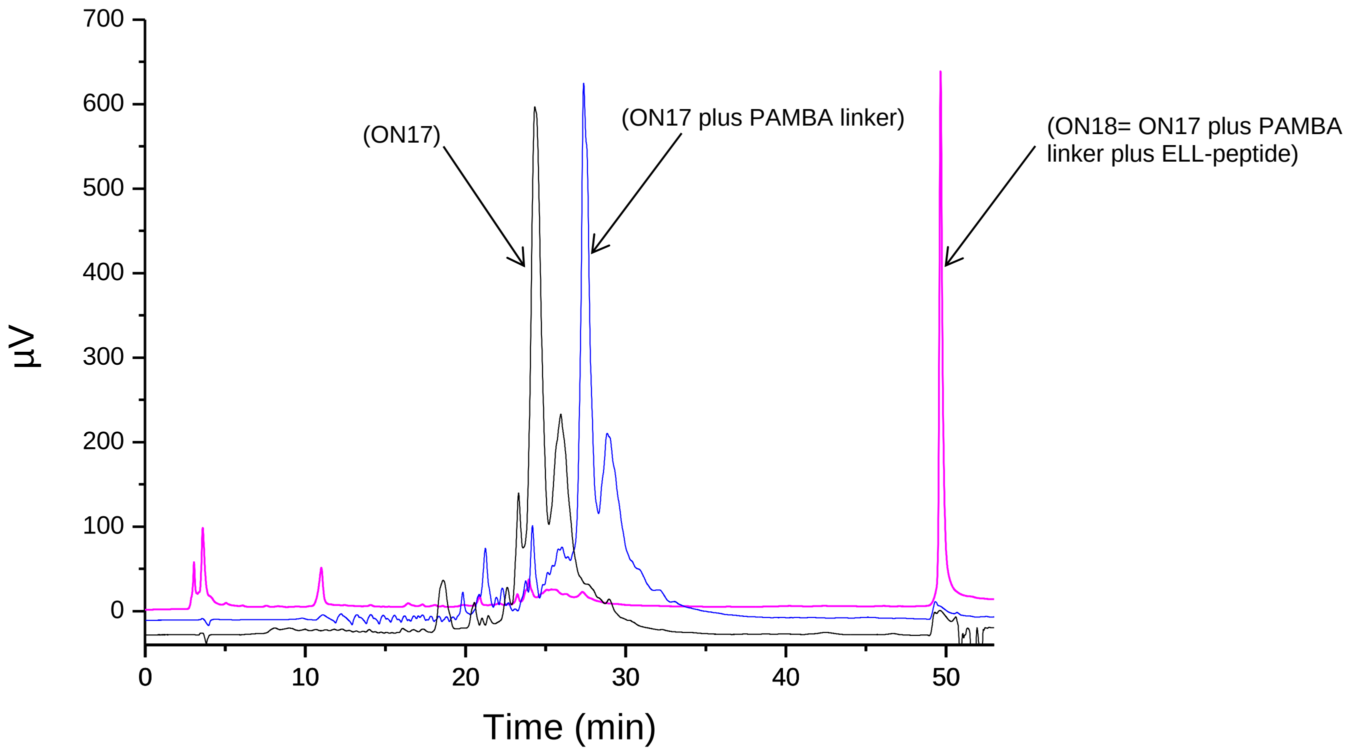

Figure S29. RP-HPLC profiles of conjugation steps during preparation of 2′-*O*-AECM-modified oligonucleotide–ELL-peptide conjugate (**ON18**). RP-HPLC profiles of crude **ON17** (t_R = 24.3 min), crude **ON17** plus PAMBA linker (t_R = 27.4 min) and crude ON–ELL-peptide conjugate **ON18** (t_R = 49.7 min, washing step) on a Supelco Discovery® BIO Wide Pore C18-5 (250 × 4.6 mm) column with a linear gradient from 0 to 50% of buffer B in buffer A over 45 min at 25 °C. Buffer (A): 50 mM triethylammonium acetate (TEAA), pH 6.5; (buffer B): 50 mM TEAA, pH 6.5 in H_2O-CH_3CN (1:1 v/v).

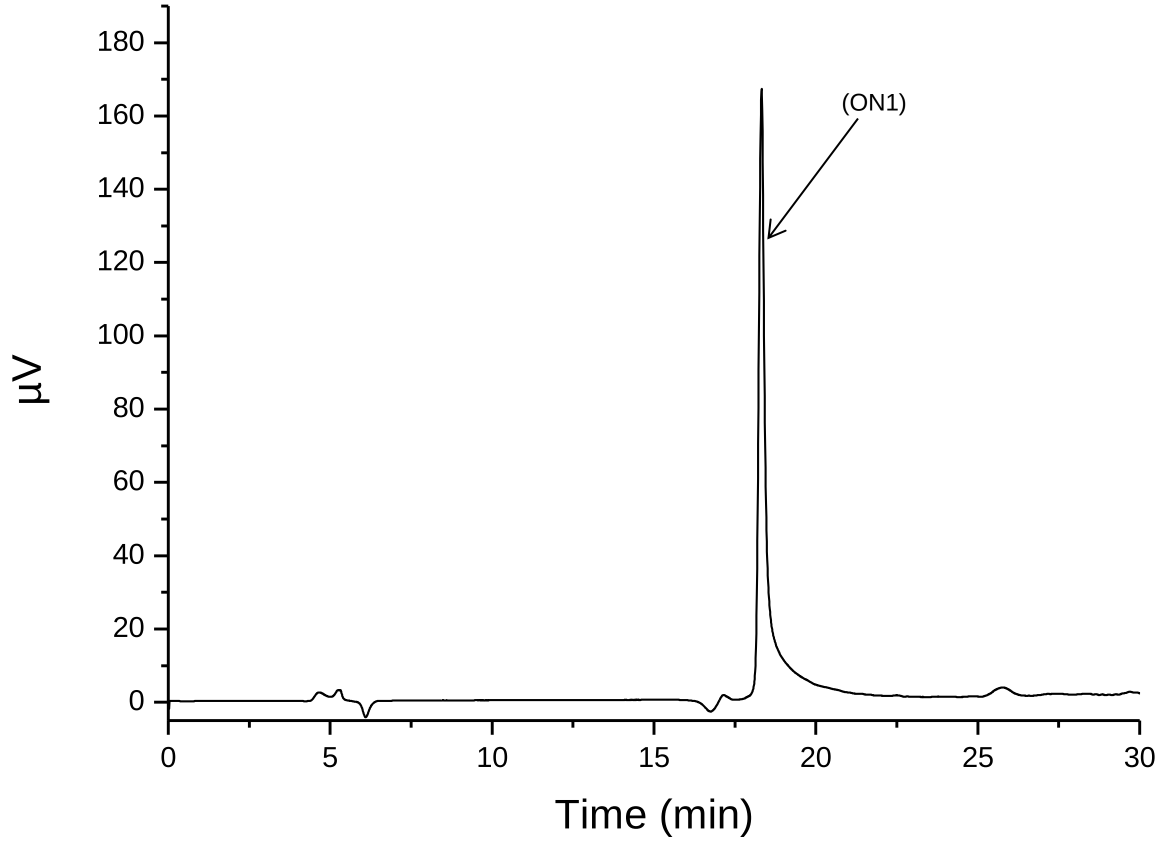

Figure S30. RP-HPLC profile of purified **ON1** on a Supelco Discovery® BIO Wide Pore C18-5 (250 × 4.6 mm) column with a linear gradient from 0 to 100% of buffer B in buffer A over 30 min at 25 °C. t_R = 18.3 min. Buffer (A): 50 mM triethylammonium acetate (TEAA), pH 6.5; (buffer B): 50 mM TEAA, pH 6.5 in H_2O-CH_3CN (1:1 v/v).

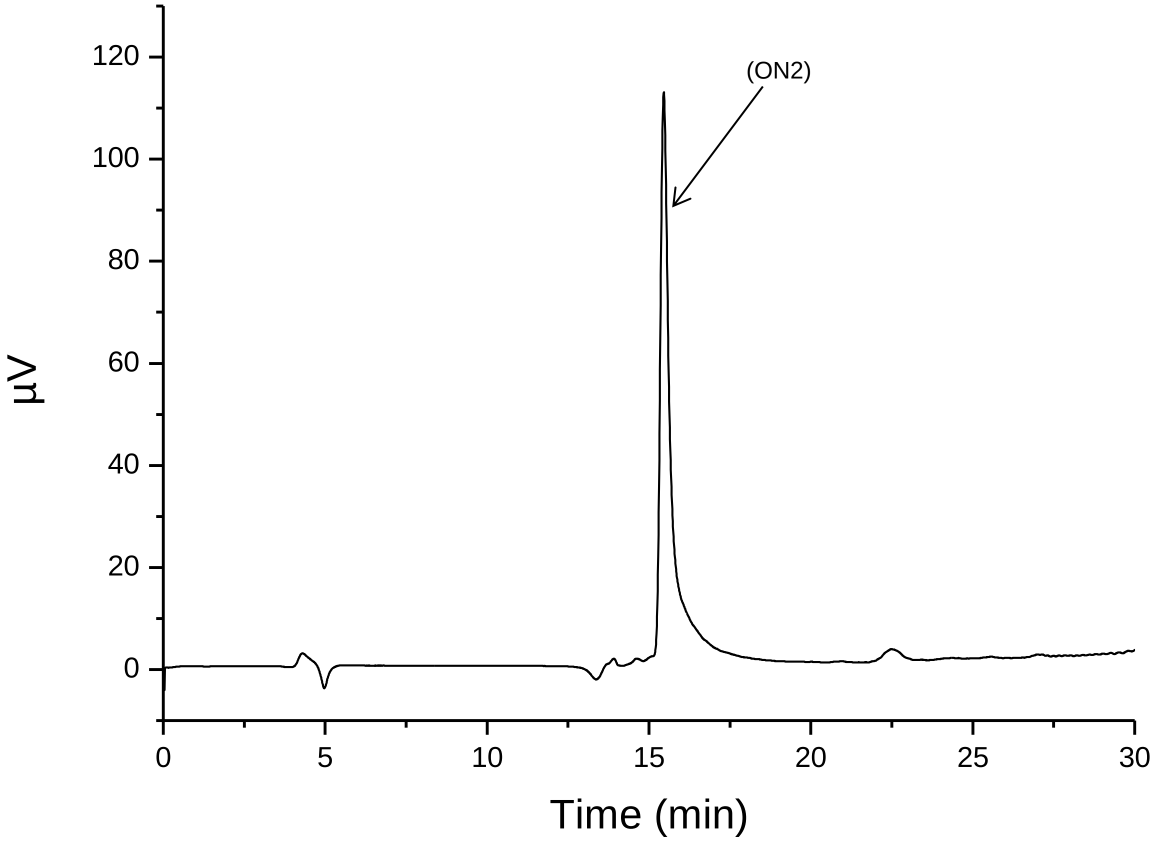

Figure S31. RP-HPLC profile of purified **ON2** on a Supelco Discovery® BIO Wide Pore C18-5 (250 × 4.6 mm) column with a linear gradient from 0 to 100% of buffer B in buffer A over 30 min at 25 °C. t_R = 15.5 min. Buffer (A): 50 mM triethylammonium acetate (TEAA), pH 6.5; (buffer B): 50 mM TEAA, pH 6.5 in H_2O-CH_3CN (1:1 v/v).

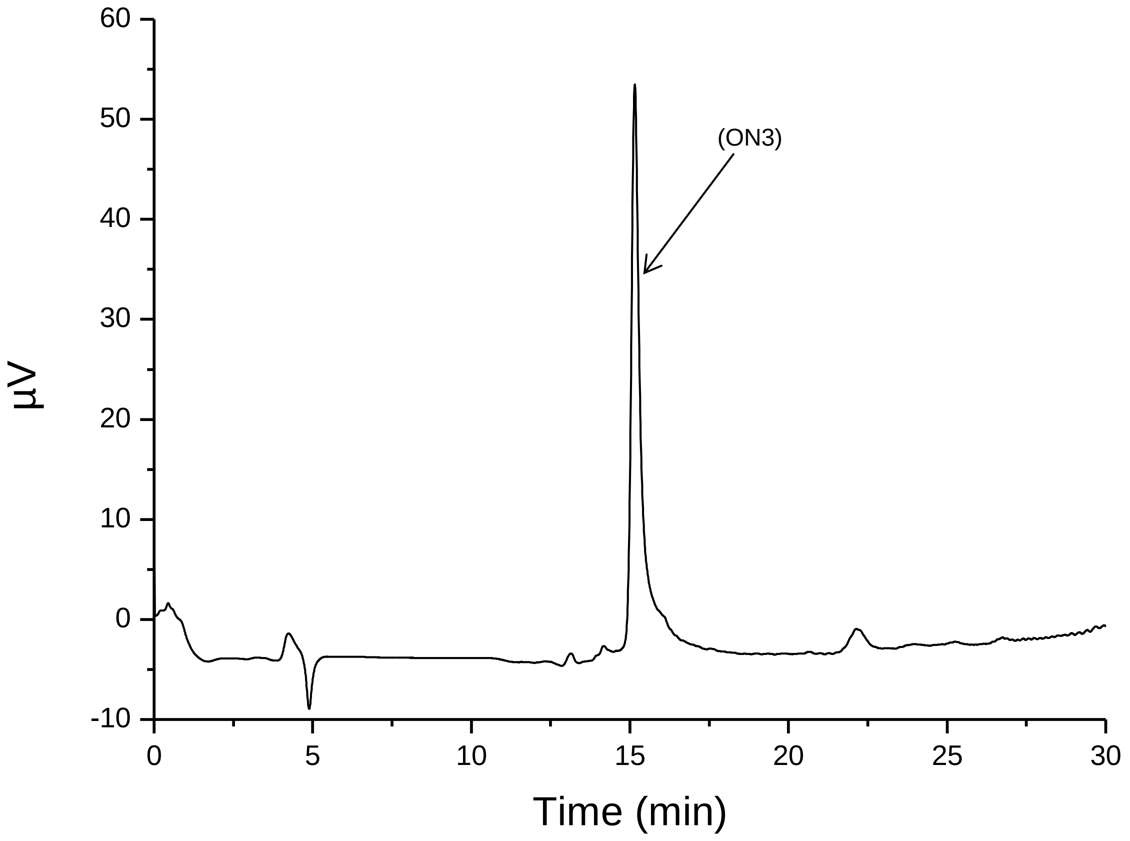

Figure S32. RP-HPLC profile of purified **ON3** on a Supelco Discovery® BIO Wide Pore C18-5 (250 × 4.6 mm) column with a linear gradient from 0 to 100% of buffer B in buffer A over 30 min at 25 °C. t_R = 15.2 min. Buffer (A): 50 mM triethylammonium acetate (TEAA), pH 6.5; (buffer B): 50 mM TEAA, pH 6.5 in H_2O-CH_3CN (1:1 v/v).

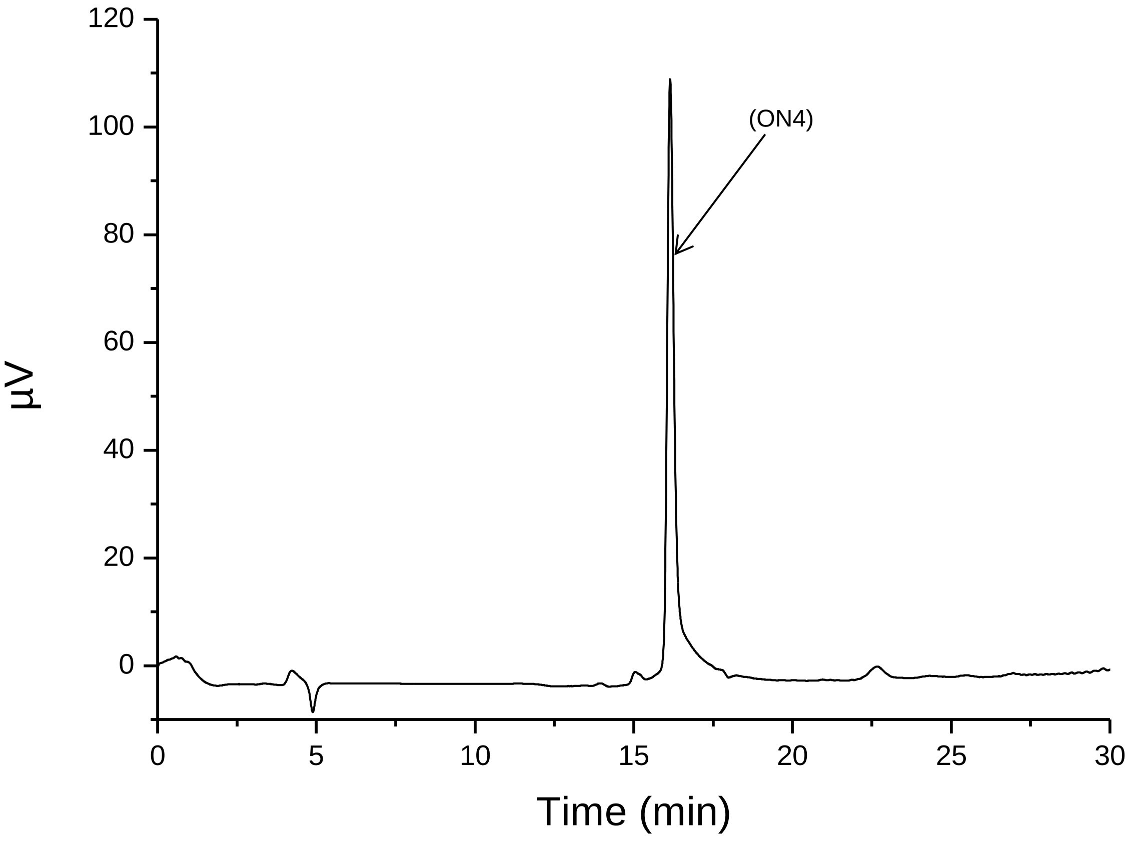

Figure S33. RP-HPLC profile of purified **ON4** on a Supelco Discovery® BIO Wide Pore C18-5 (250 × 4.6 mm) column with a linear gradient from 0 to 100% of buffer B in buffer A over 30 min at 25 °C. t_R = 16.1 min. Buffer (A): 50 mM triethylammonium acetate (TEAA), pH 6.5; (buffer B): 50 mM TEAA, pH 6.5 in H_2O-CH_3CN (1:1 v/v).

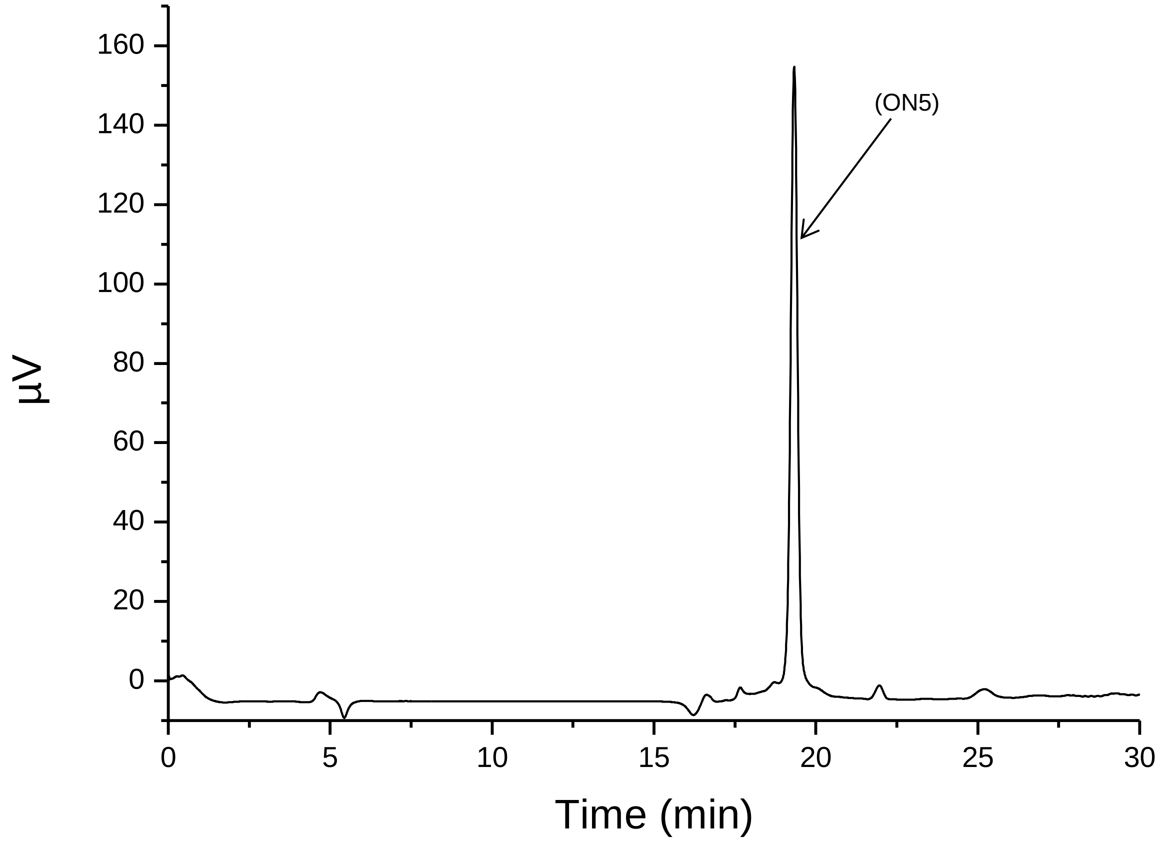

Figure S34. RP-HPLC profile of purified **ON5** on a Supelco Discovery® BIO Wide Pore C18-5 (250 × 4.6 mm) column with a linear gradient from 0 to 100% of buffer B in buffer A over 30 min at 25 °C. t_R = 19.3 min. Buffer (A): 50 mM triethylammonium acetate (TEAA), pH 6.5; (buffer B): 50 mM TEAA, pH 6.5 in H_2O-CH_3CN (1:1 v/v).

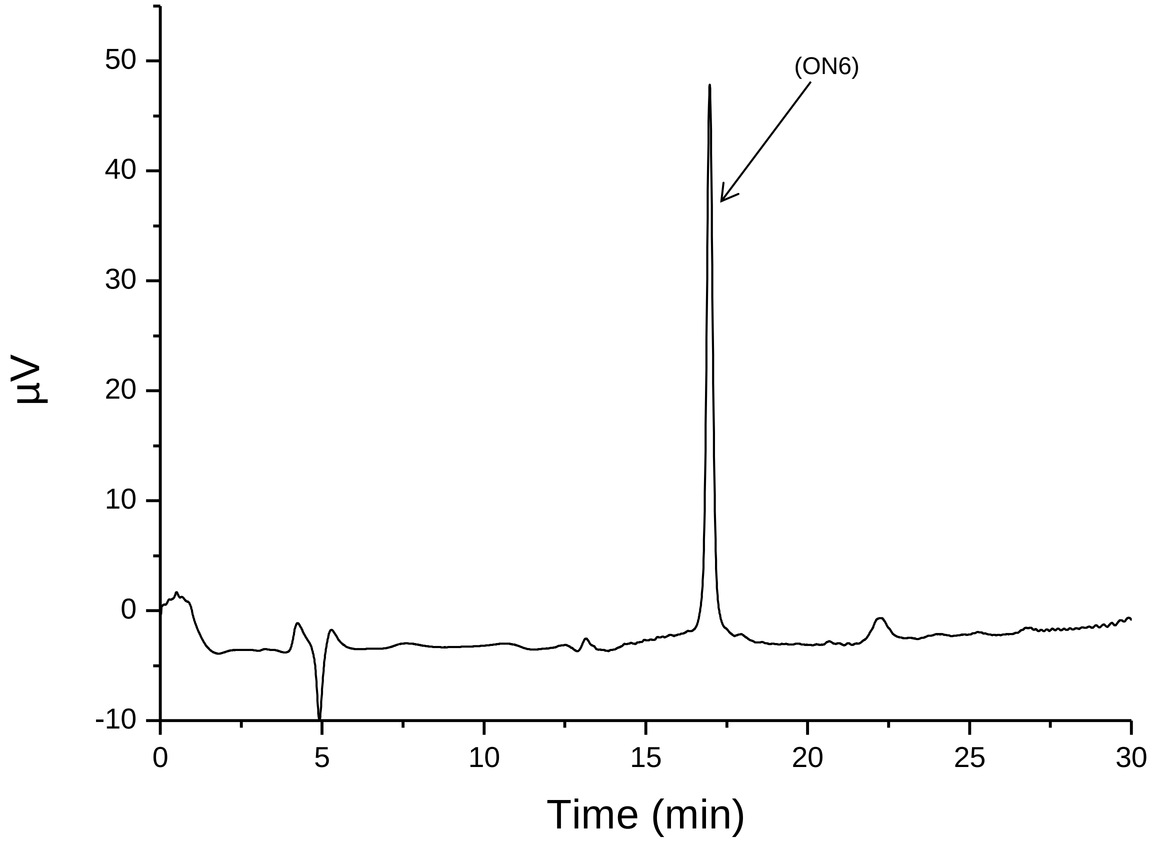

Figure S35. RP-HPLC profile of purified **ON6** on a Supelco Discovery® BIO Wide Pore C18-5 (250 × 4.6 mm) column with a linear gradient from 0 to 100% of buffer B in buffer A over 30 min at 25 °C. t_R = 17.0 min. Buffer (A): 50 mM triethylammonium acetate (TEAA), pH 6.5; (buffer B): 50 mM TEAA, pH 6.5 in H_2O-CH_3CN (1:1 v/v).

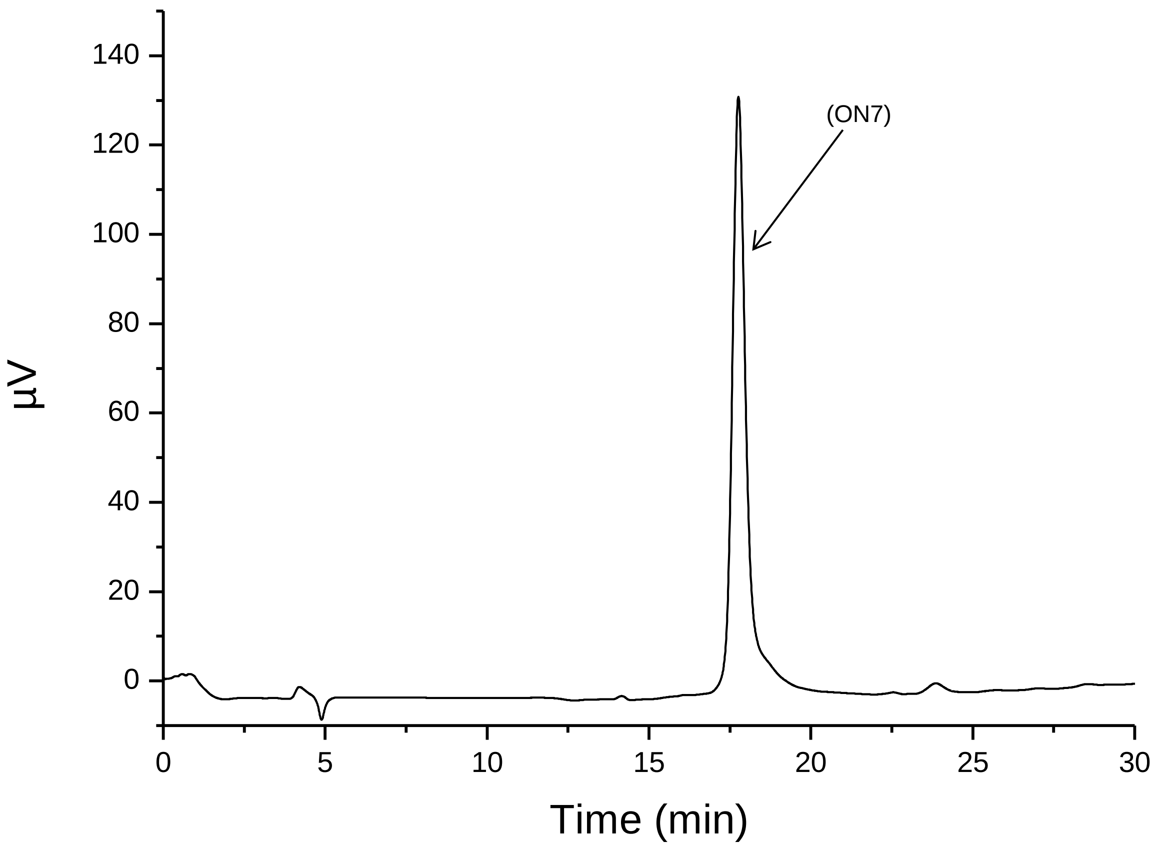

Figure S36. RP-HPLC profile of purified **ON7** on a Supelco Discovery® BIO Wide Pore C18-5 (250 × 4.6 mm) column with a linear gradient from 0 to 100% of buffer B in buffer A over 30 min at 25 °C. t_R = 17.7 min. Buffer (A): 50 mM triethylammonium acetate (TEAA), pH 6.5; (buffer B): 50 mM TEAA, pH 6.5 in H_2O-CH_3CN (1:1 v/v).

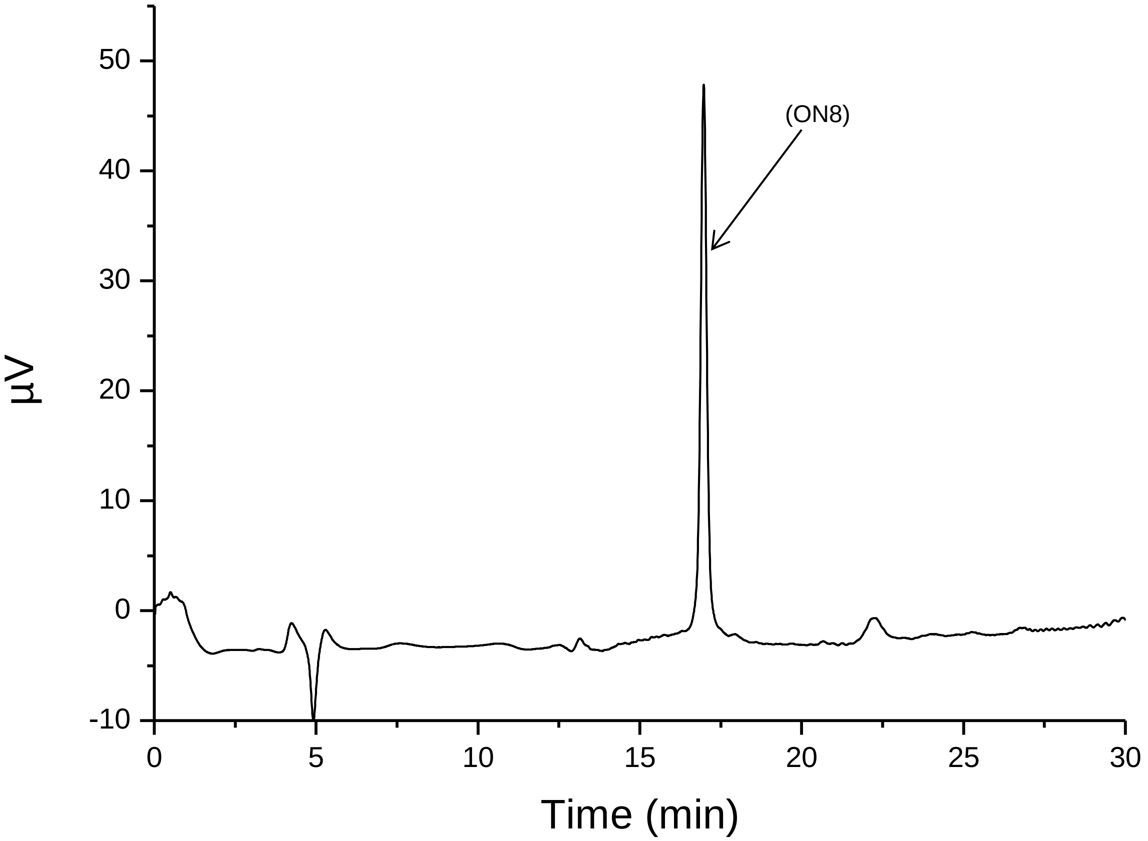

Figure S37. RP-HPLC profile of purified **ON8** on a Supelco Discovery® BIO Wide Pore C18-5 (250 × 4.6 mm) column with a linear gradient from 0 to 100% of buffer B in buffer A over 30 min at 25 °C. t_R = 17.7 min. Buffer (A): 50 mM triethylammonium acetate (TEAA), pH 6.5; (buffer B): 50 mM TEAA, pH 6.5 in H_2O-CH_3CN (1:1 v/v).

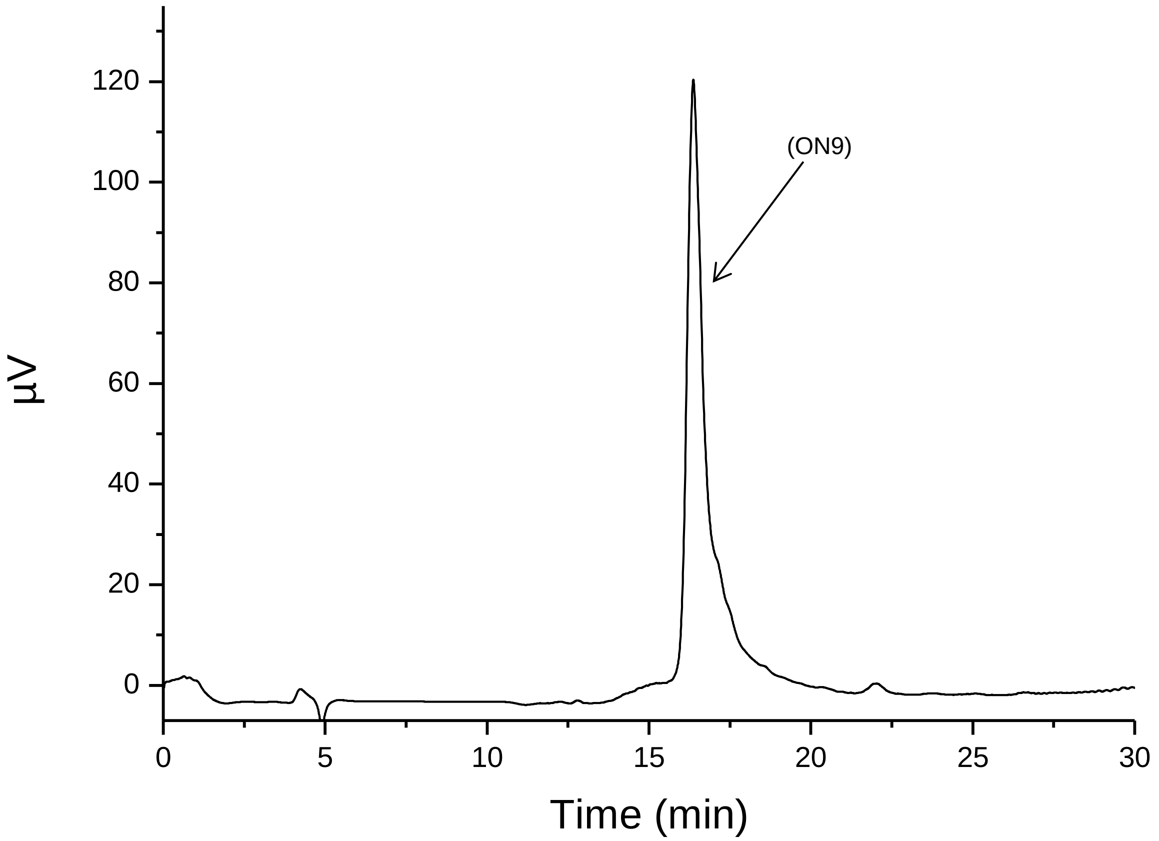

Figure S38. RP-HPLC profile of purified **ON9** on a Supelco Discovery® BIO Wide Pore C18-5 (250 × 4.6 mm) column with a linear gradient from 0 to 100% of buffer B in buffer A over 30 min at 25 °C. t_R = 16.4 min. Buffer (A): 50 mM triethylammonium acetate (TEAA), pH 6.5; (buffer B): 50 mM TEAA, pH 6.5 in H_2O-CH_3CN (1:1 v/v).

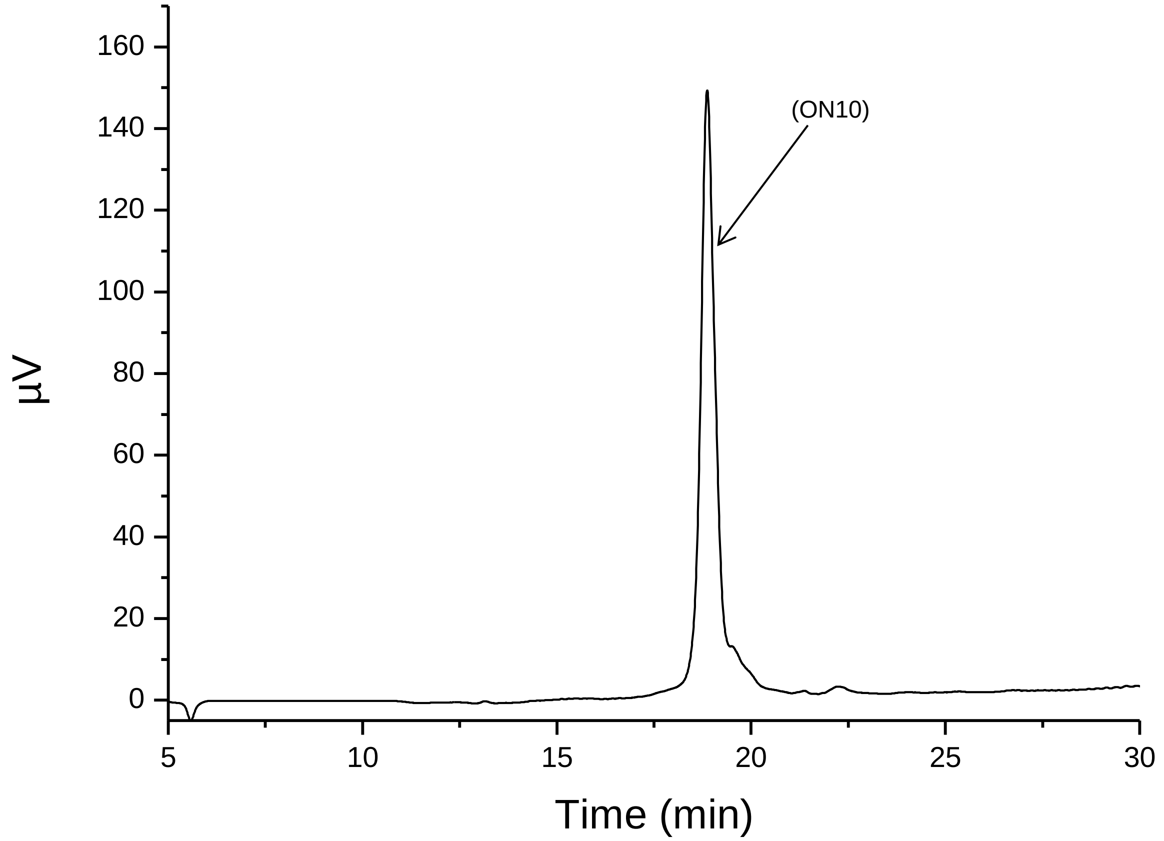

Figure S39. RP-HPLC profile of purified **ON10** on a Supelco Discovery® BIO Wide Pore C18-5 (250 × 4.6 mm) column with a linear gradient from 0 to 100% of buffer B in buffer A over 30 min at 25 °C. t_R = 18.9 min. Buffer (A): 50 mM triethylammonium acetate (TEAA), pH 6.5; (buffer B): 50 mM TEAA, pH 6.5 in H_2O-CH_3CN (1:1 v/v).

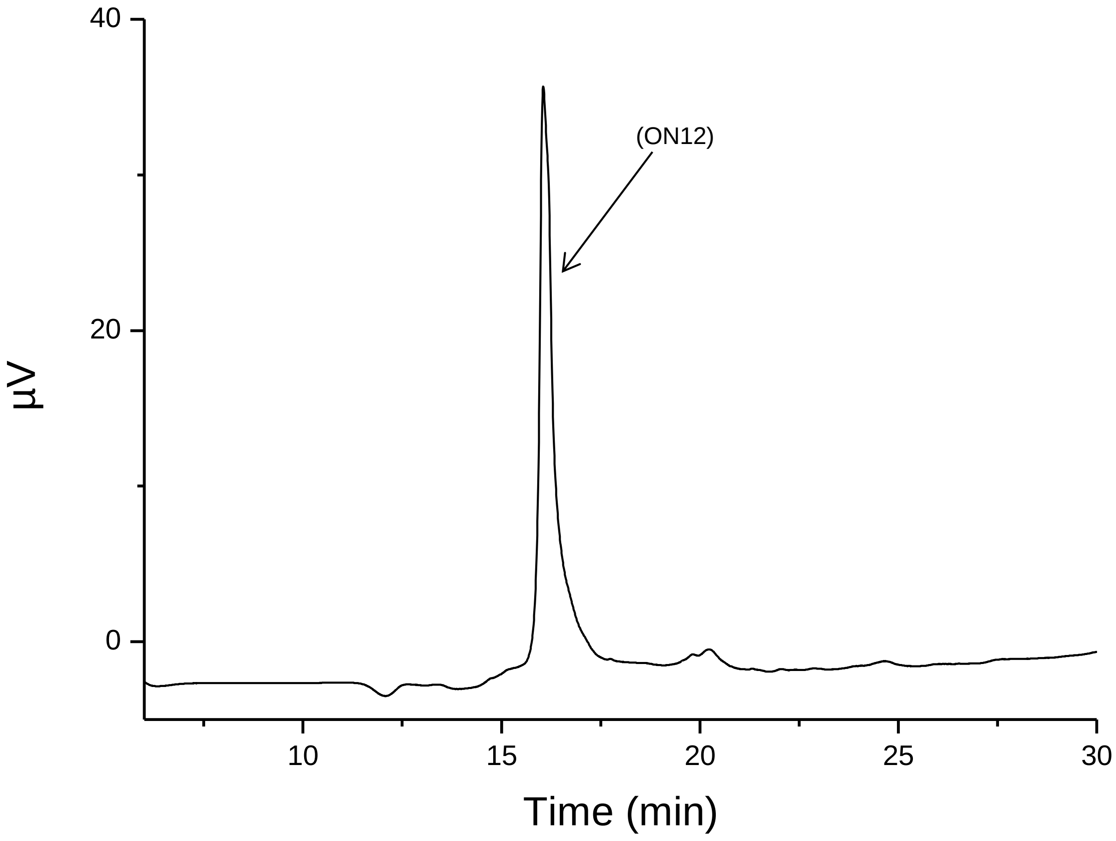

Figure S40. RP-HPLC profile of purified **ON12** on a Supelco Discovery® BIO Wide Pore C18-5 (250 × 4.6 mm) column with a linear gradient from 0 to 100% of buffer B in buffer A over 30 min at 25 °C. t_R = 16.1 min. Buffer (A): 50 mM triethylammonium acetate (TEAA), pH 6.5; (buffer B): 50 mM TEAA, pH 6.5 in H_2O-CH_3CN (1:1 v/v).

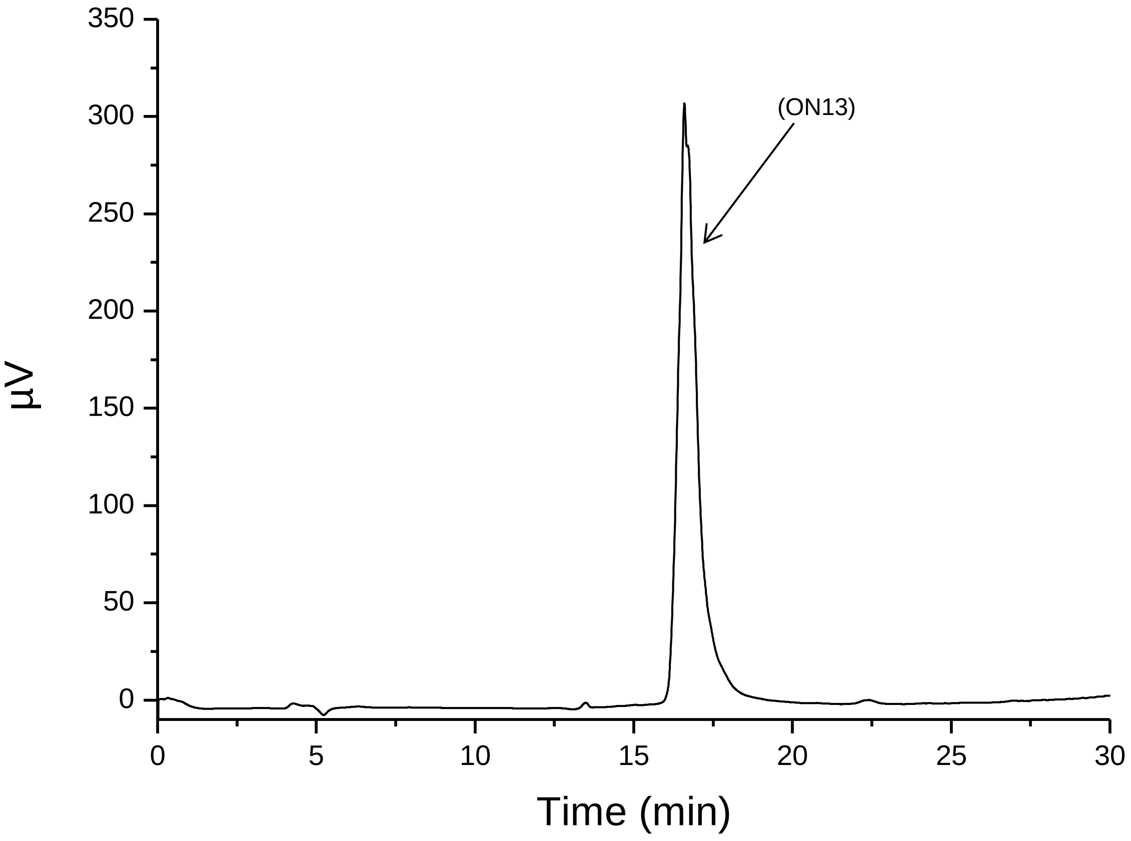

Figure S41. RP-HPLC profile of purified **ON13** on a Supelco Discovery® BIO Wide Pore C18-5 (250 × 4.6 mm) column with a linear gradient from 0 to 100% of buffer B in buffer A over 30 min at 25 °C. t_R = 16.2 min. Buffer (A): 50 mM triethylammonium acetate (TEAA), pH 6.5; (buffer B): 50 mM TEAA, pH 6.5 in H_2O-CH_3CN (1:1 v/v).

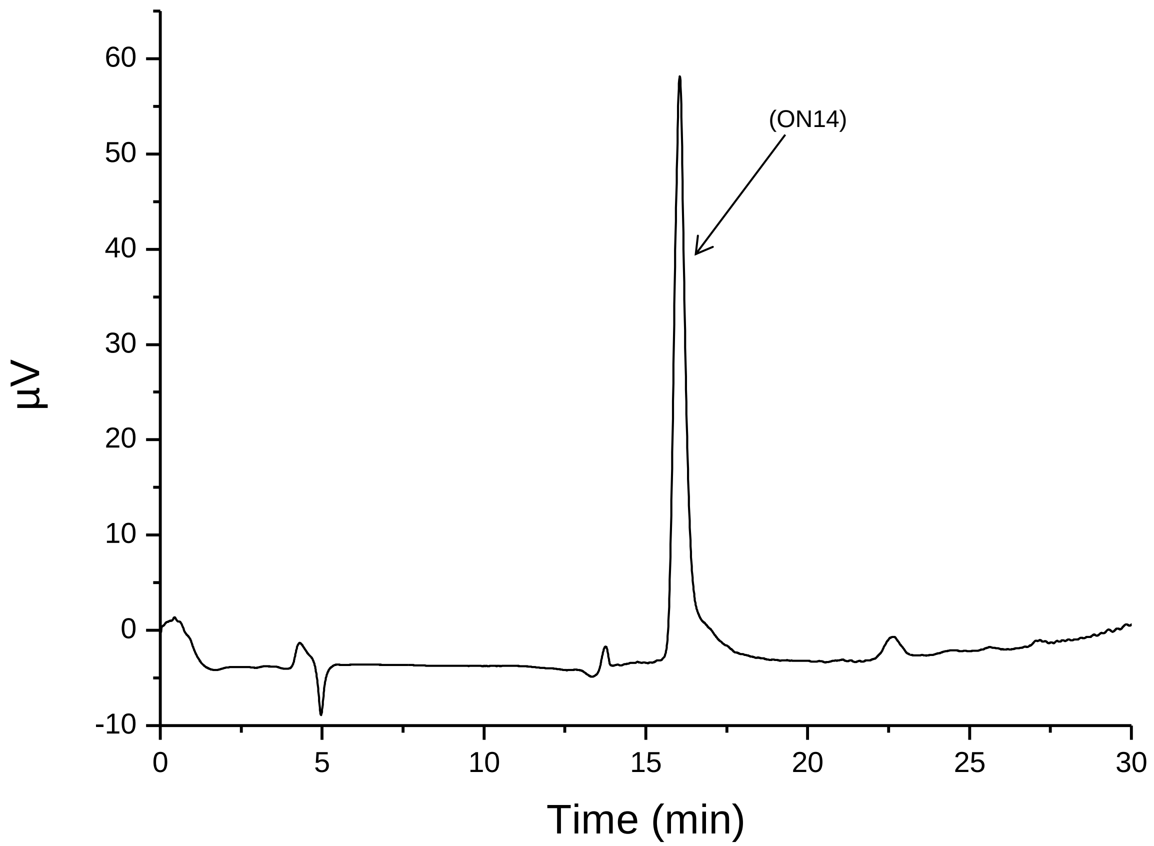

Figure S42. RP-HPLC profile of purified **ON14** on a Supelco Discovery® BIO Wide Pore C18-5 (250 × 4.6 mm) column with a linear gradient from 0 to 100% of buffer B in buffer A over 30 min at 25 °C. t_R = 16.05 min. Buffer (A): 50 mM triethylammonium acetate (TEAA), pH 6.5; (buffer B): 50 mM TEAA, pH 6.5 in H_2O-CH_3CN (1:1 v/v).

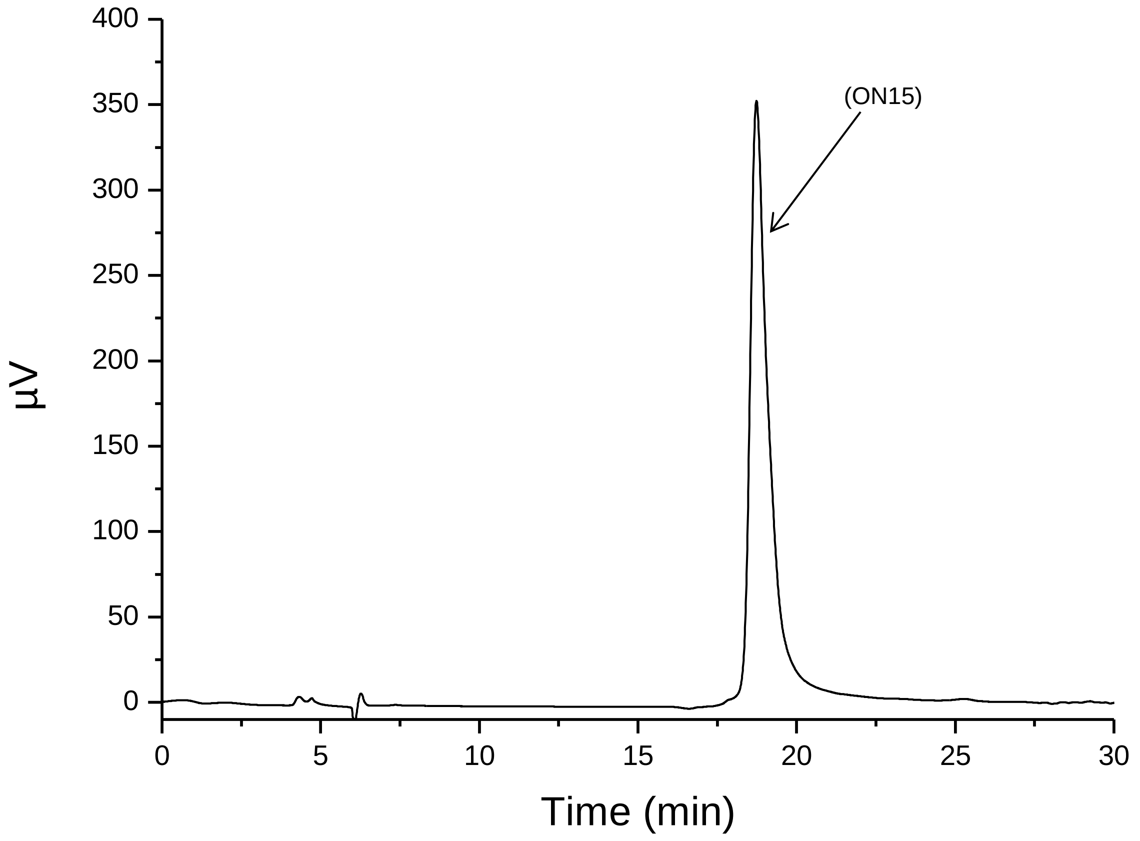

Figure S43. RP-HPLC profile of purified **ON15** on a Supelco Discovery® BIO Wide Pore C18-5 (250 × 4.6 mm) column with a linear gradient from 0 to 100% of buffer B in buffer A over 30 min at 25 °C. t_R = 18.7 min. Buffer (A): 50 mM triethylammonium acetate (TEAA), pH 6.5; (buffer B): 50 mM TEAA, pH 6.5 in H_2O-CH_3CN (1:1 v/v).

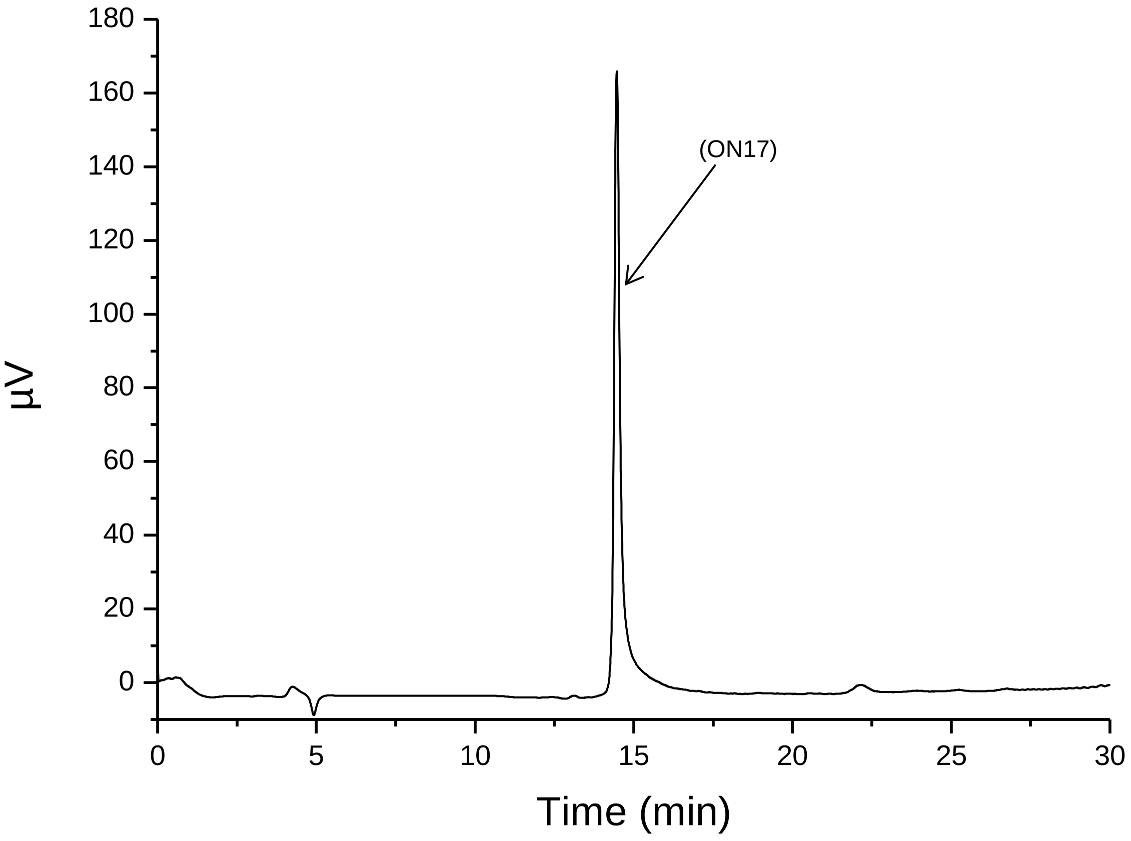

Figure S44. RP-HPLC profile of purified **ON17** on a Supelco Discovery® BIO Wide Pore C18-5 (250 × 4.6 mm) column with a linear gradient from 0 to 100% of buffer B in buffer A over 30 min at 25 °C. t_R = 14.5 min. Buffer (A): 50 mM triethylammonium acetate (TEAA), pH 6.5; (buffer B): 50 mM TEAA, pH 6.5 in H_2O-CH_3CN (1:1 v/v).

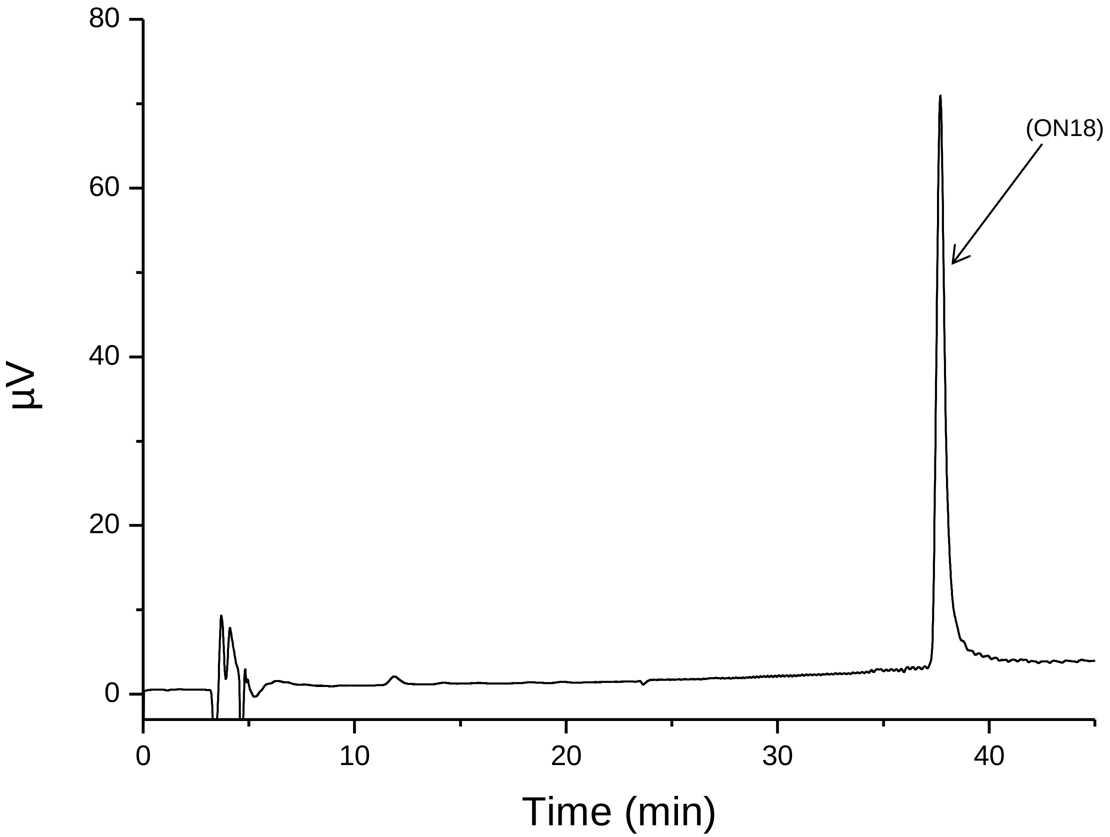

Figure S45. RP-HPLC profile of purified **ON18** on a Supelco Discovery® BIO Wide Pore C18-5 (250 × 4.6 mm) column with a linear gradient from 50 to 100% of buffer B in buffer A over 45 min at 25 °C. t_R = 37.7 min. Buffer (A): 50 mM triethylammonium acetate (TEAA), pH 6.5; (buffer B): 50 mM TEAA, pH 6.5 in H_2O-CH_3CN (1:1 v/v).

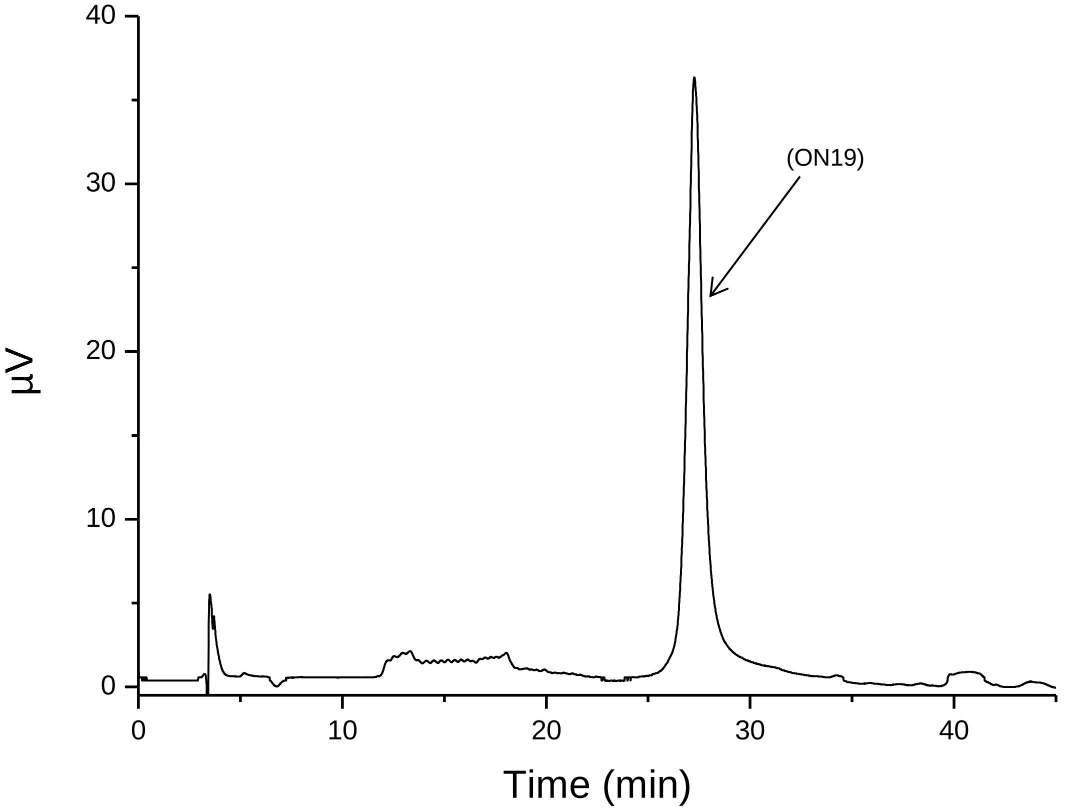

Figure S46. RP-HPLC profile of purified **ON19** on a Supelco Discovery® BIO Wide Pore C18-5 (250 × 4.6 mm) column with a linear gradient from 0 to 50% of buffer B in buffer A over 45 min at 25 °C. t_R = 27.3 min. Buffer (A): 50 mM triethylammonium acetate (TEAA), pH 6.5; (buffer B): 50 mM TEAA, pH 6.5 in H_2O-CH_3CN (1:1 v/v).

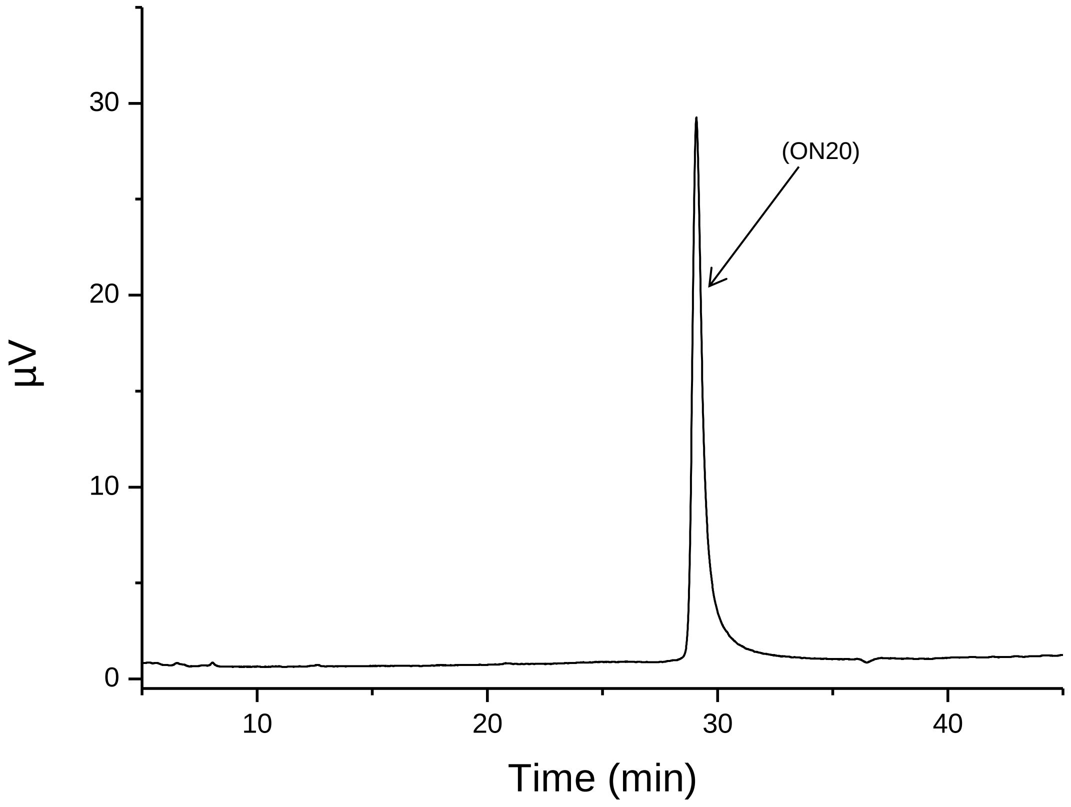

Figure S47. RP-HPLC profile of purified **ON20** on a Supelco Discovery® BIO Wide Pore C18-5 (250 × 4.6 mm) column with a linear gradient from 50 to 100% of buffer B in buffer A over 45 min at 25 °C. t_R = 29.0 min. Buffer (A): 50 mM triethylammonium acetate (TEAA), pH 6.5; (buffer B): 50 mM TEAA, pH 6.5 in H_2O-CH_3CN (1:1 v/v).

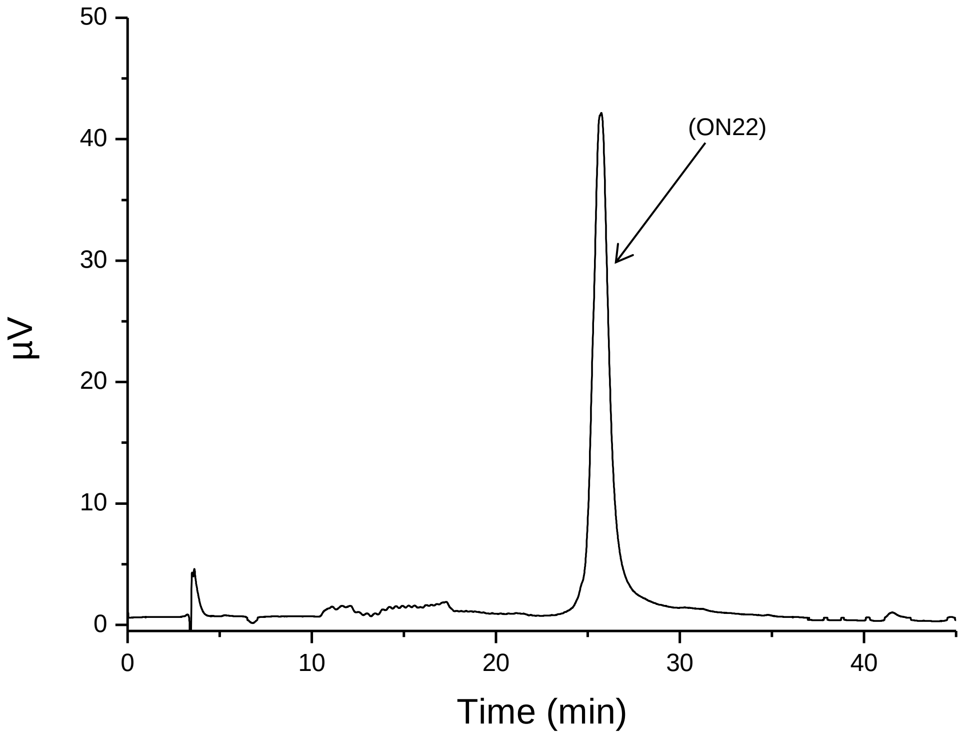

Figure S48. RP-HPLC profile of purified **ON22** on a Supelco Discovery® BIO Wide Pore C18-5 (250 × 4.6 mm) column with a linear gradient from 0 to 50% of buffer B in buffer A over 45 min at 25 °C. t_R = 25.7 min. Buffer (A): 50 mM triethylammonium acetate (TEAA), pH 6.5; (buffer B): 50 mM TEAA, pH 6.5 in H_2O-CH_3CN (1:1 v/v).

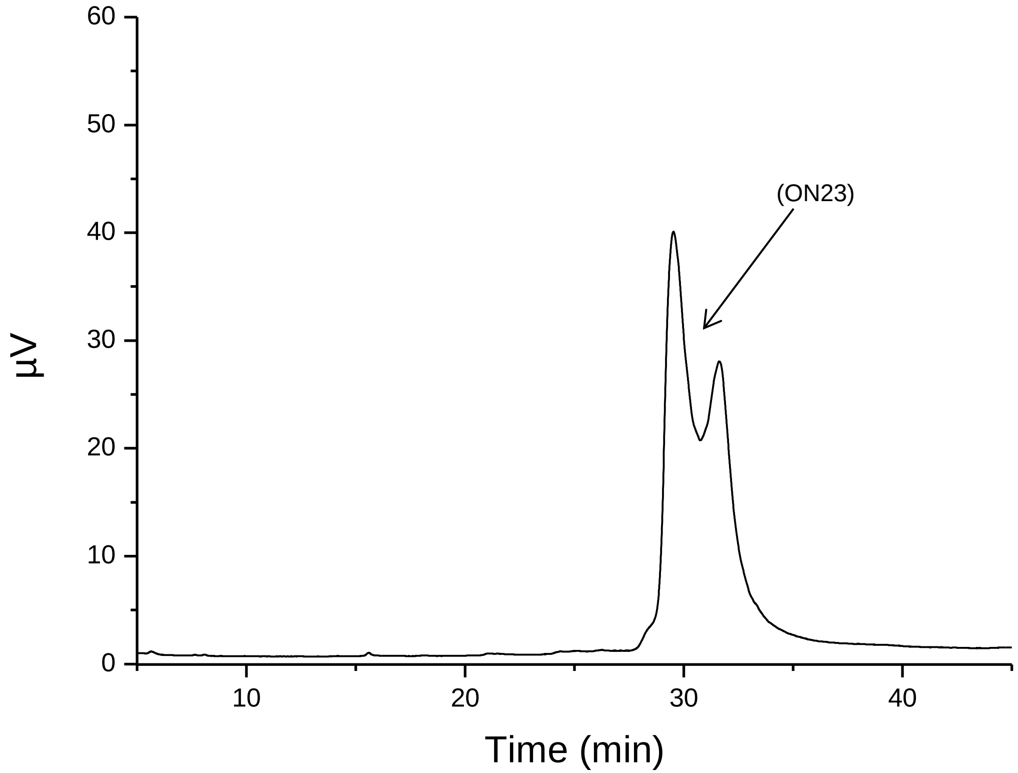

Figure S49. RP-HPLC profile of purified **ON23** on a Supelco Discovery® BIO Wide Pore C18-5 (250 × 4.6 mm) column with a linear gradient from 50 to 100% of buffer B in buffer A over 45 min at 25 °C. t_R = 30.7 min. Buffer (A): 50 mM triethylammonium acetate (TEAA), pH 6.5; (buffer B): 50 mM TEAA, pH 6.5 in H_2O-CH_3CN (1:1 v/v).

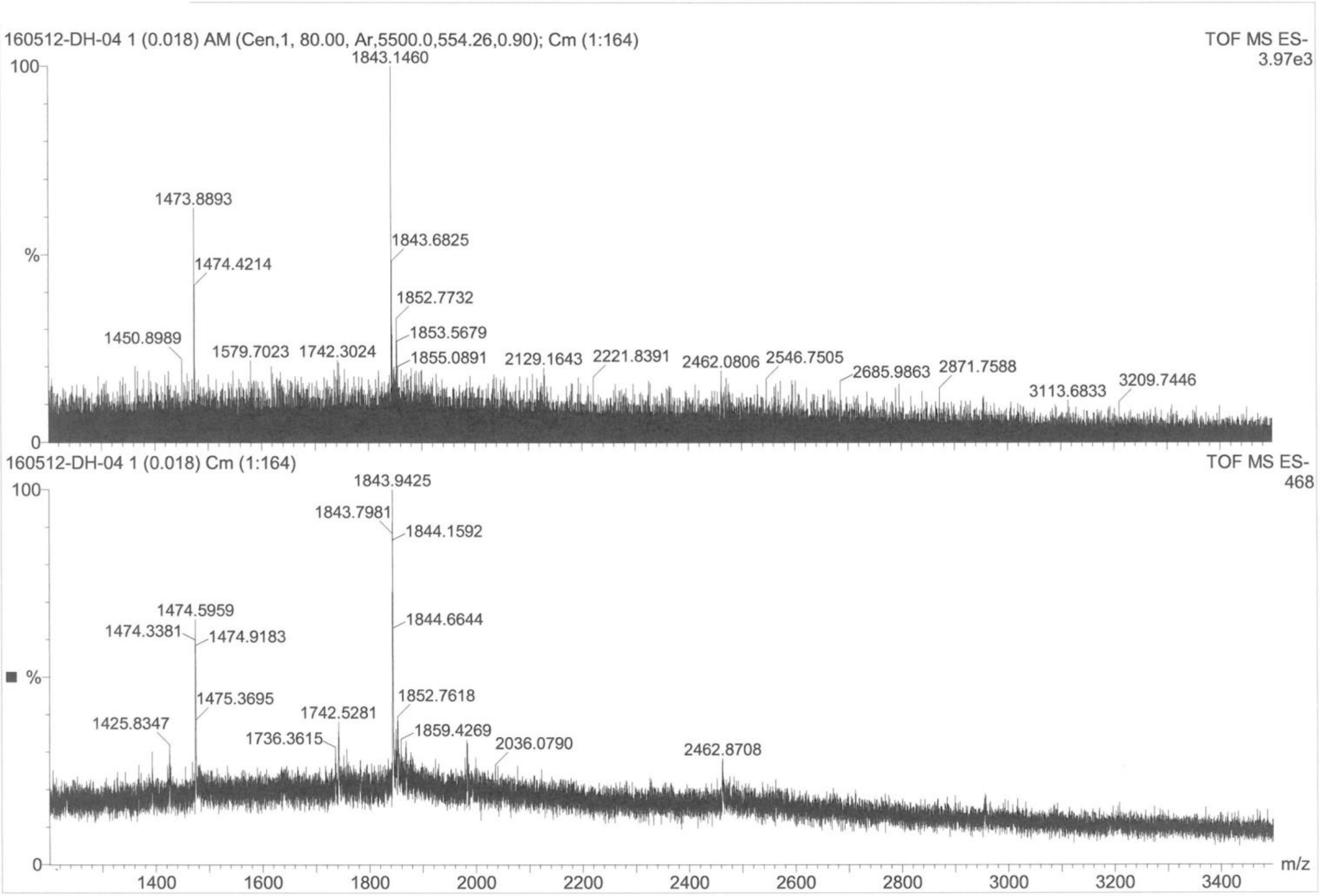

Figure S50. ESI-TOF mass spectrum of **ON1** (upper panel, leucine encephalin $[M]^{-}$ 554.2615 used as internal standard).

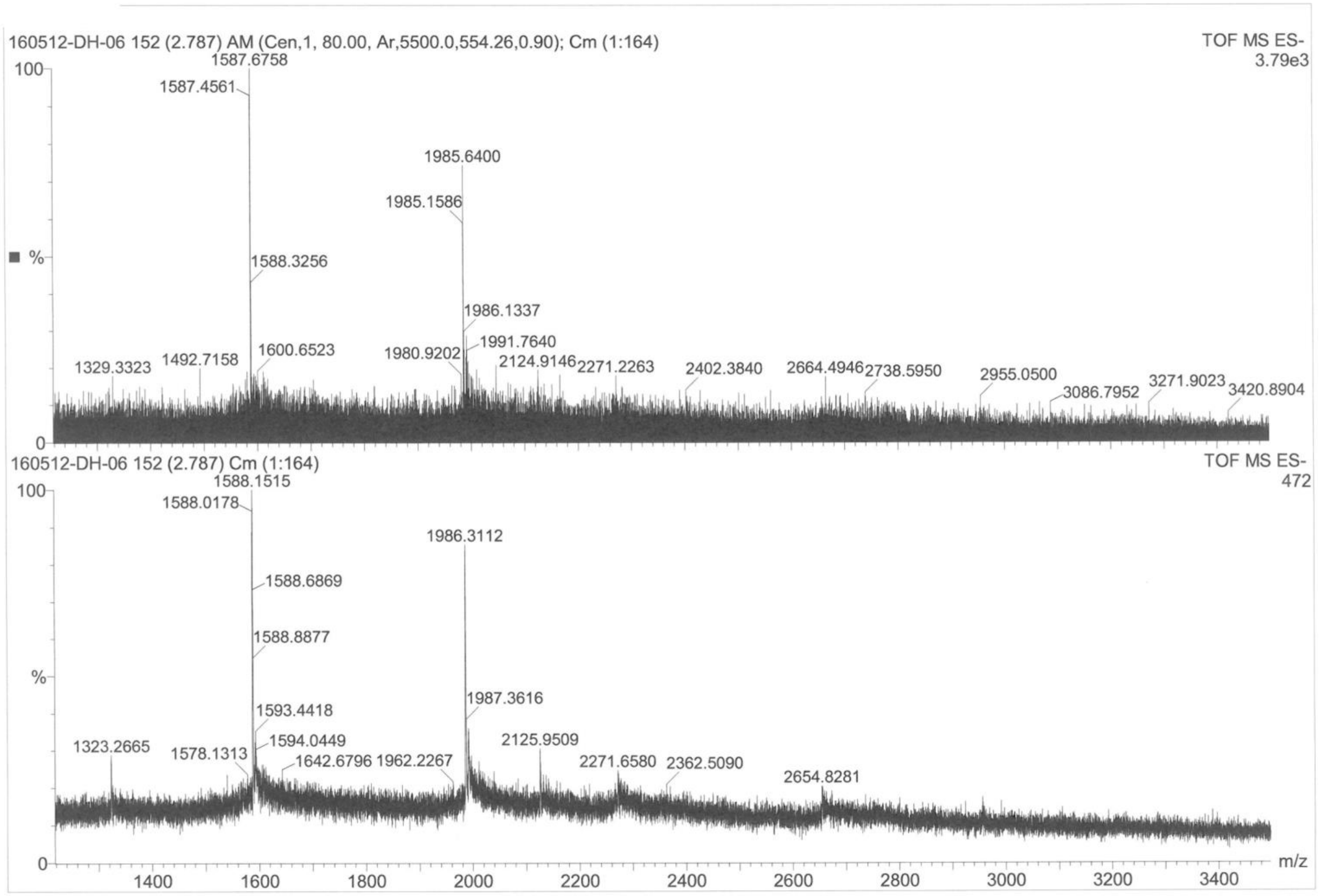

Figure S51. ESI-TOF mass spectrum of **ON2** (upper panel, leucine encephalin $[M]^-$ 554.2615 used as internal standard).

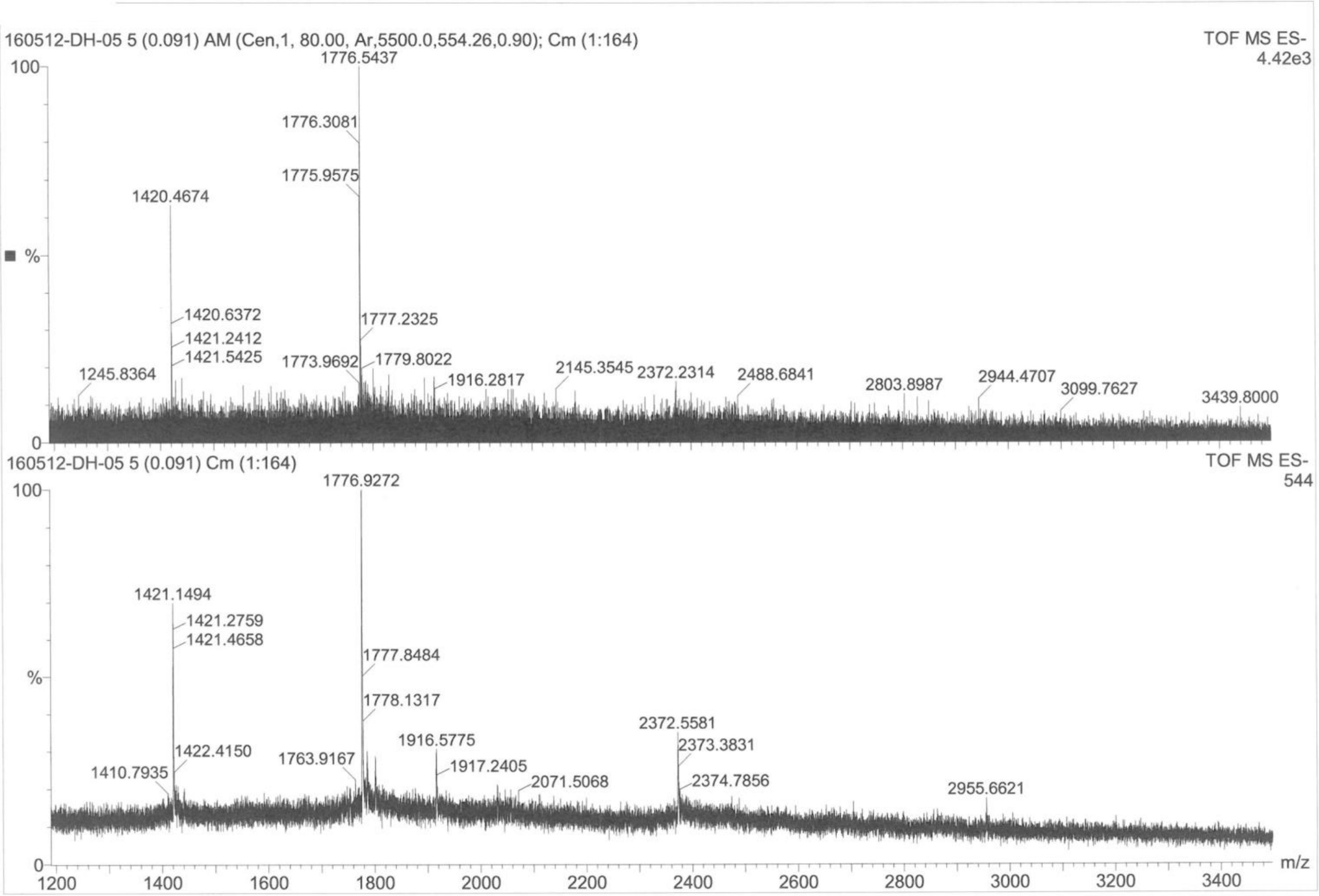

Figure S52. ESI-TOF mass spectrum of **ON3** (upper panel, leucine encephalin $[M]^-$ 554.2615 used as internal standard).

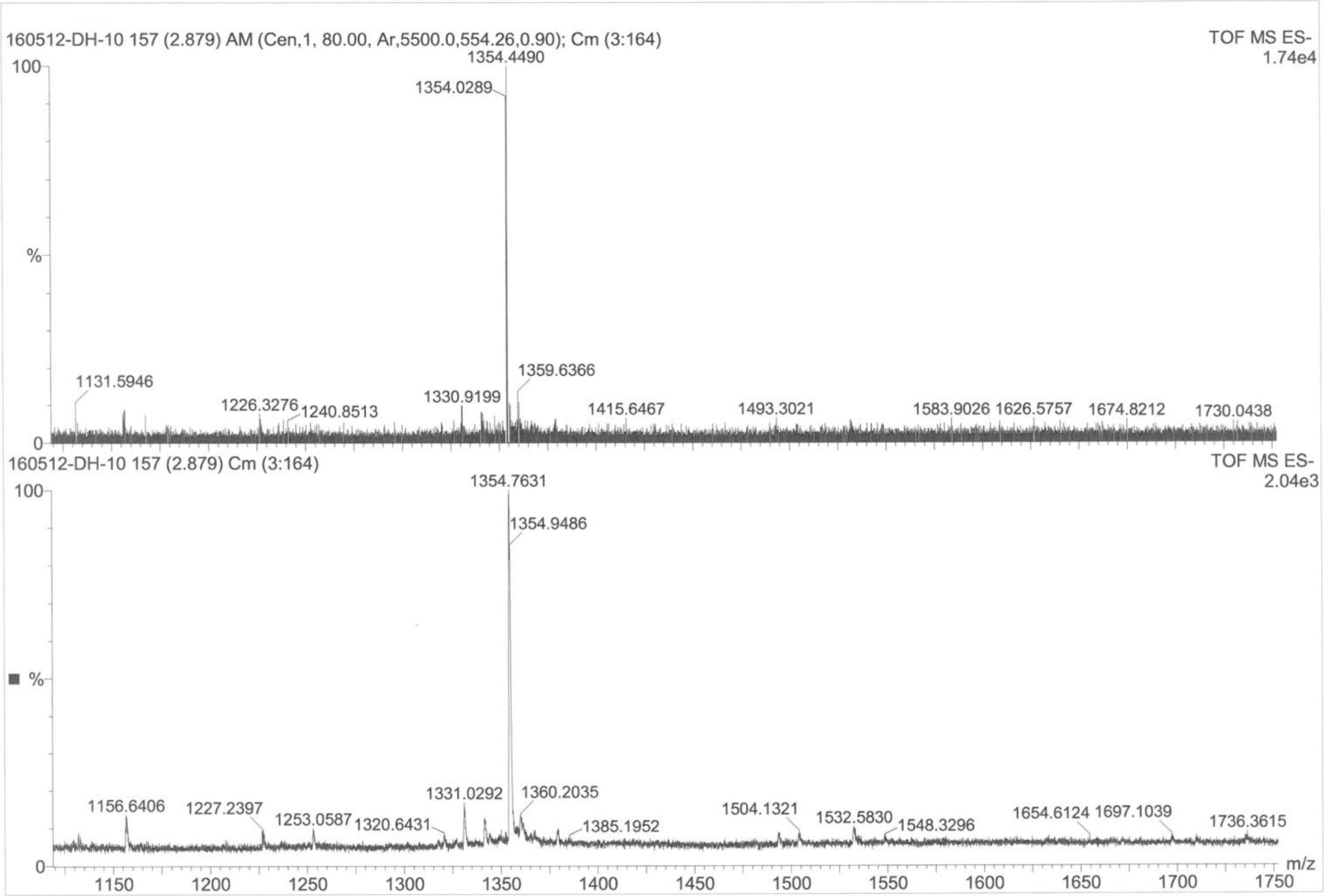

Figure S53. ESI-TOF mass spectrum of **ON4** (upper panel, leucine encephalin [M]$^{-}$ 554.2615 used as internal standard).

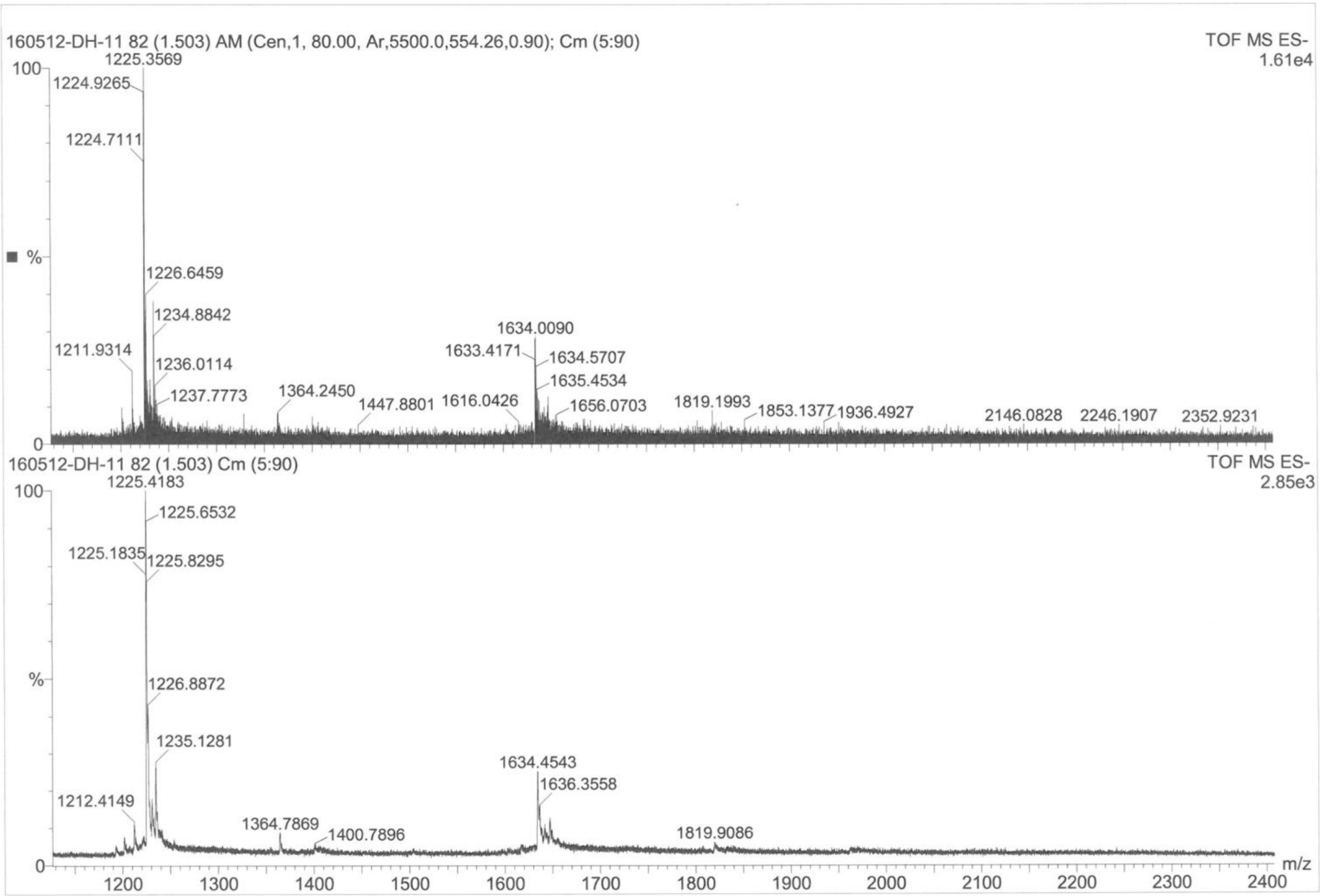

Figure S54. ESI-TOF mass spectrum of **ON5** (upper panel, leucine encephalin $[M]^{-}$ 554.2615 used as internal standard).

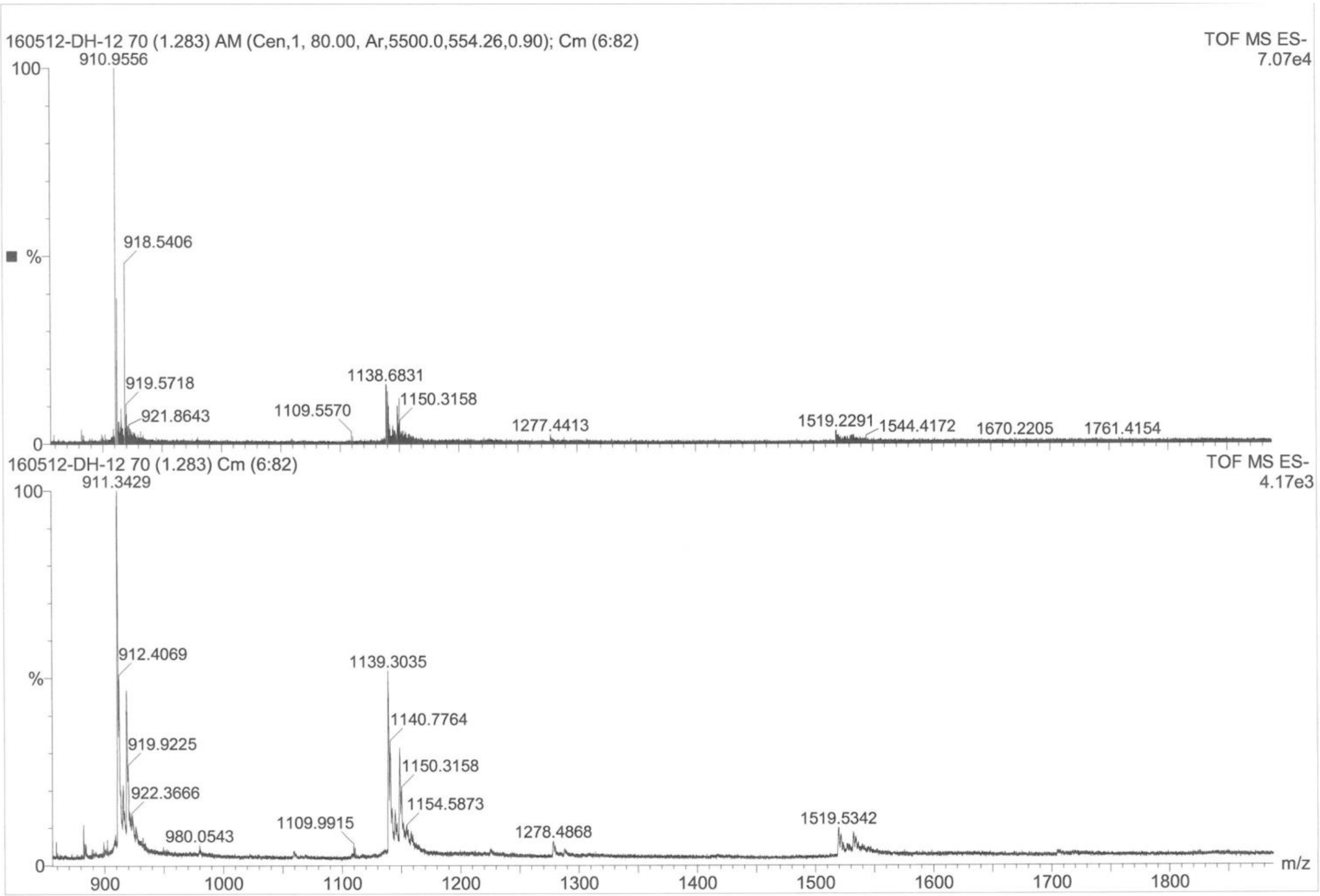

Figure S55. ESI-TOF mass spectrum of **ON6** (upper panel, leucine encephalin $[M]^-$ 554.2615 used as internal standard).

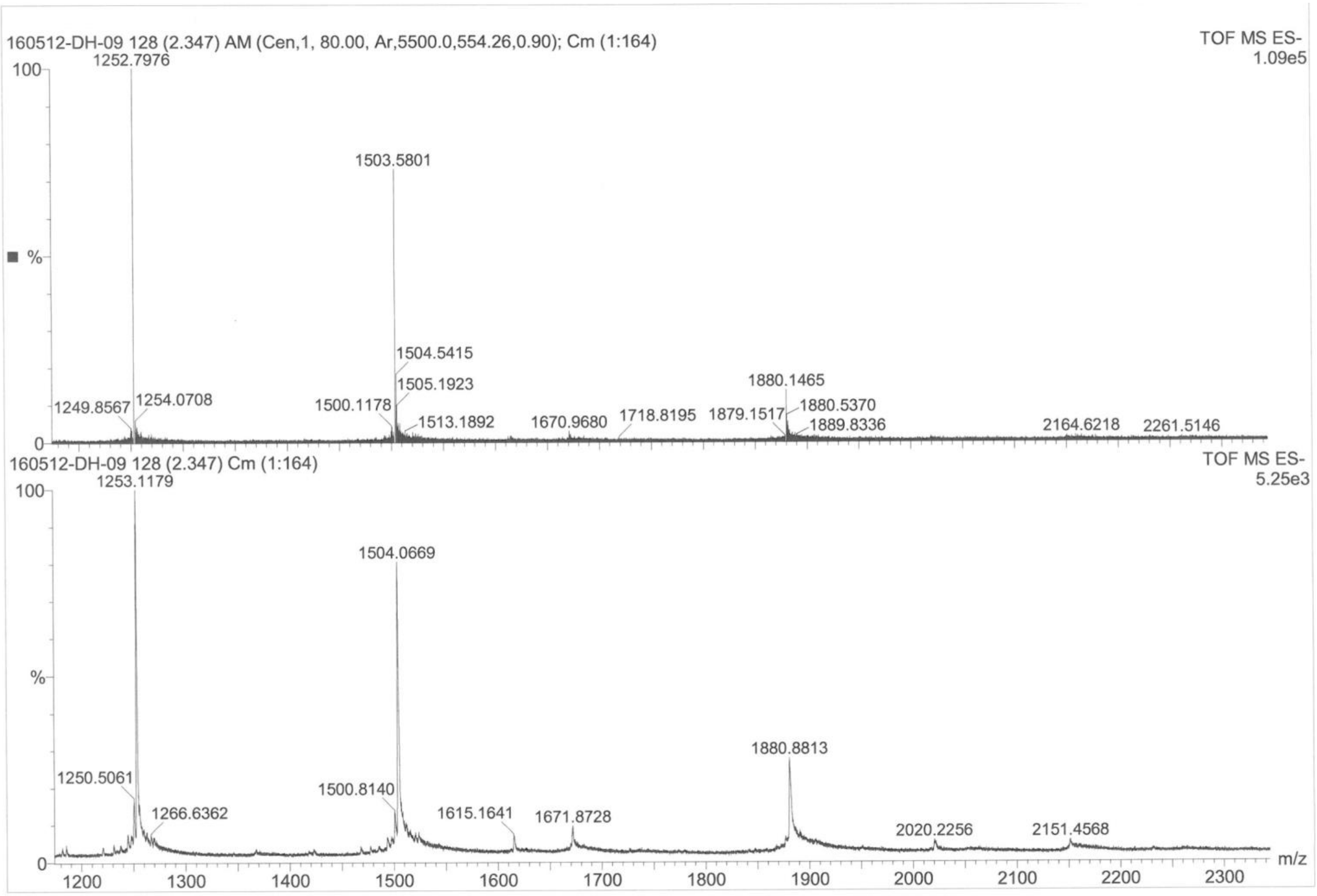

Figure S56. ESI-TOF mass spectrum of **ON7** (upper panel, leucine encephalin $[M]^-$ 554.2615 used as internal standard).

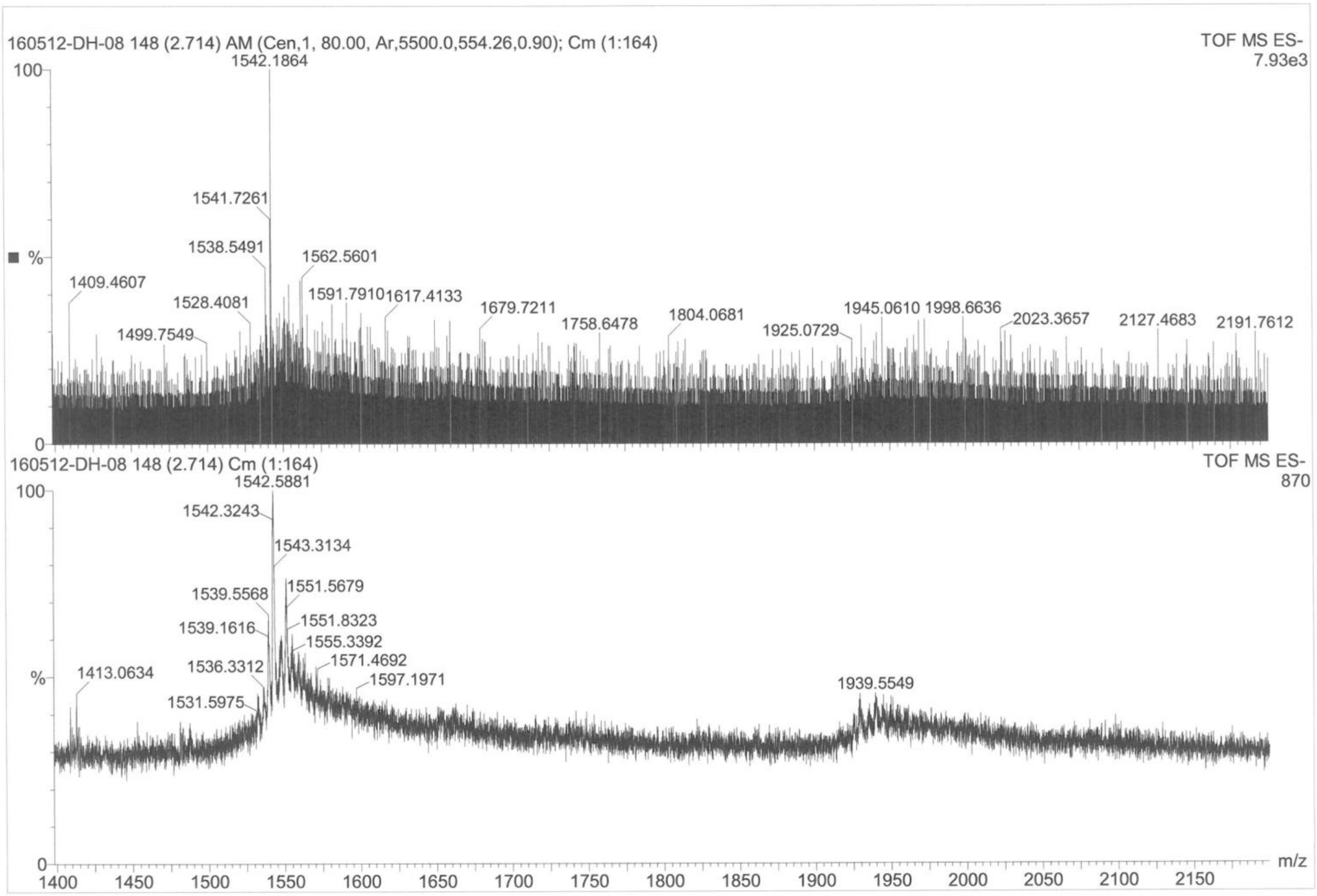

Figure S57. ESI-TOF mass spectrum of **ON8** (upper panel, leucine encephalin $[M]^-$ 554.2615 used as internal standard).

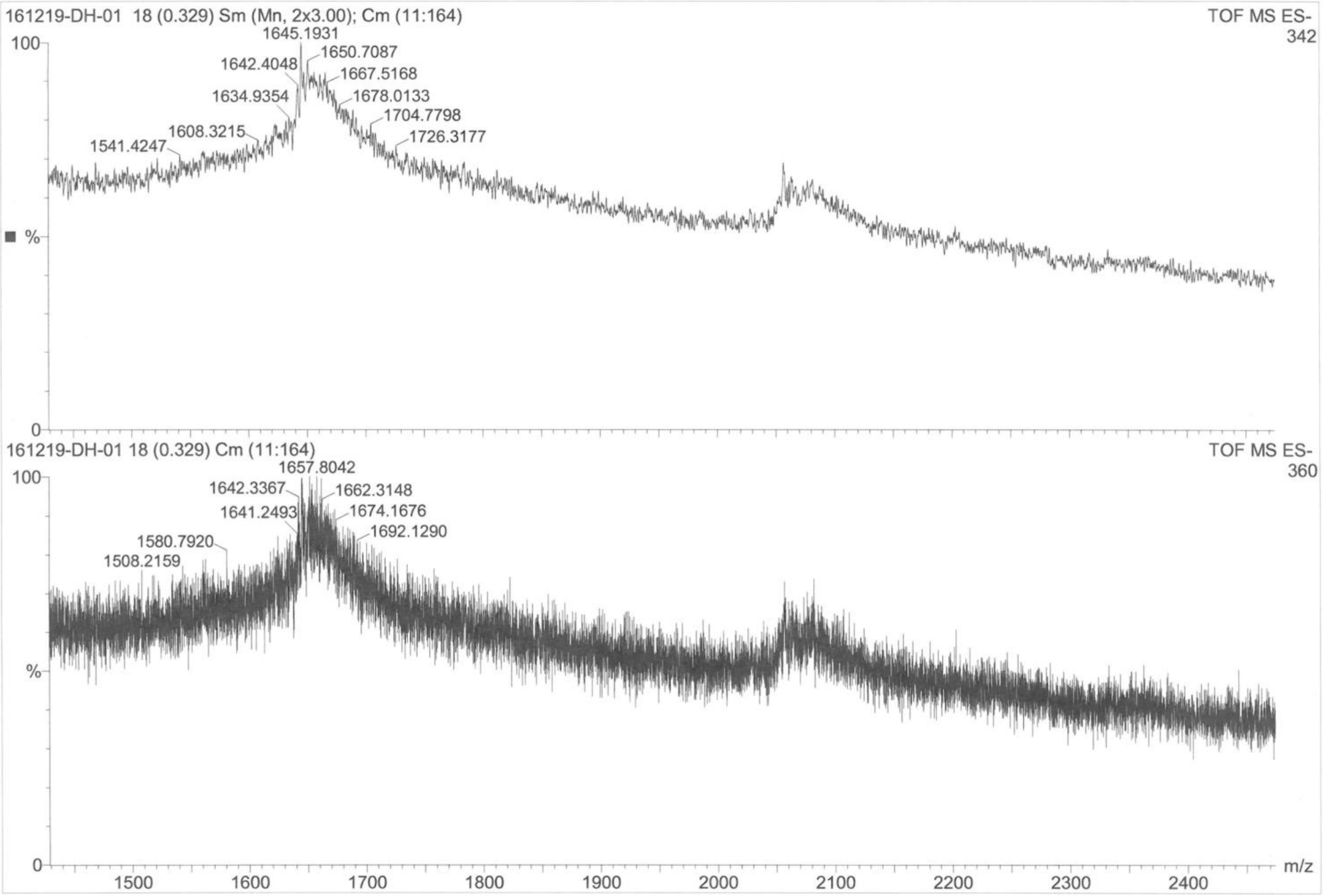

Figure S58. ESI-TOF mass spectrum of **ON9** (upper panel, leucine encephalin $[M]^-$ 554.2615 used as internal standard).

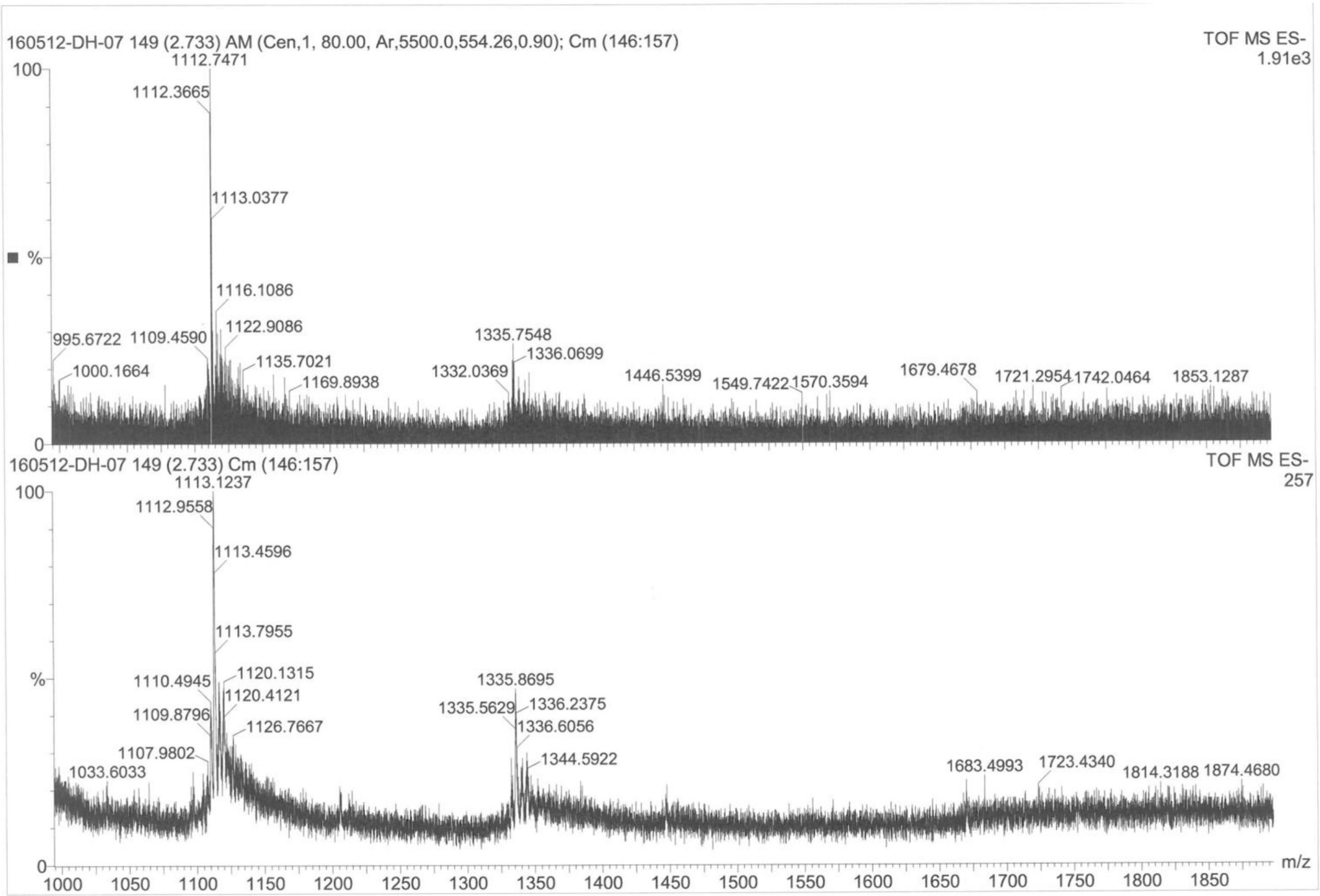

Figure S59. ESI-TOF mass spectrum of **ON10** (upper panel, leucine encephalin $[M]^-$ 554.2615 used as internal standard).

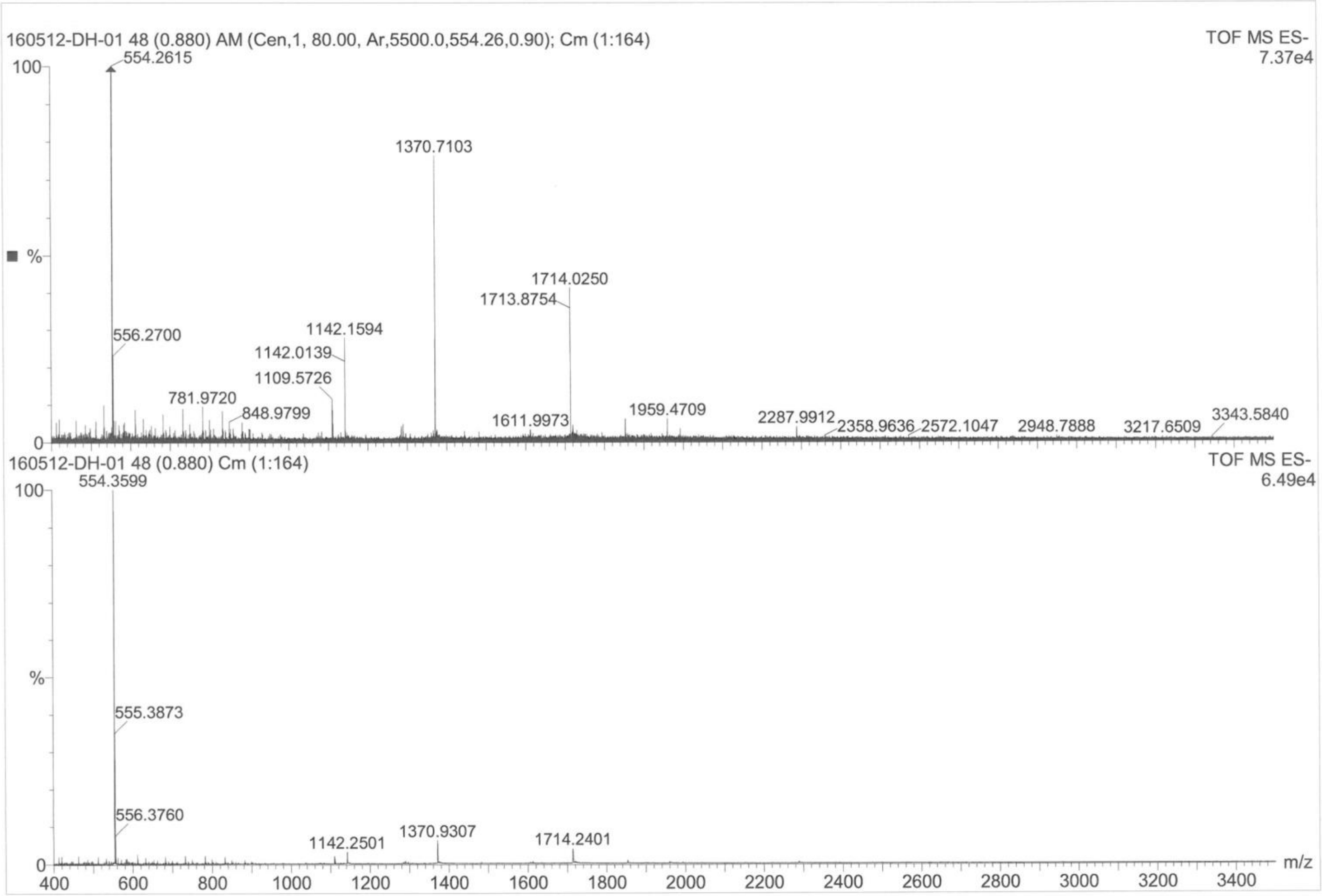

Figure S60. ESI-TOF mass spectrum of **ON12** (upper panel, leucine encephalin $[M]^-$ 554.2615 used as internal standard).

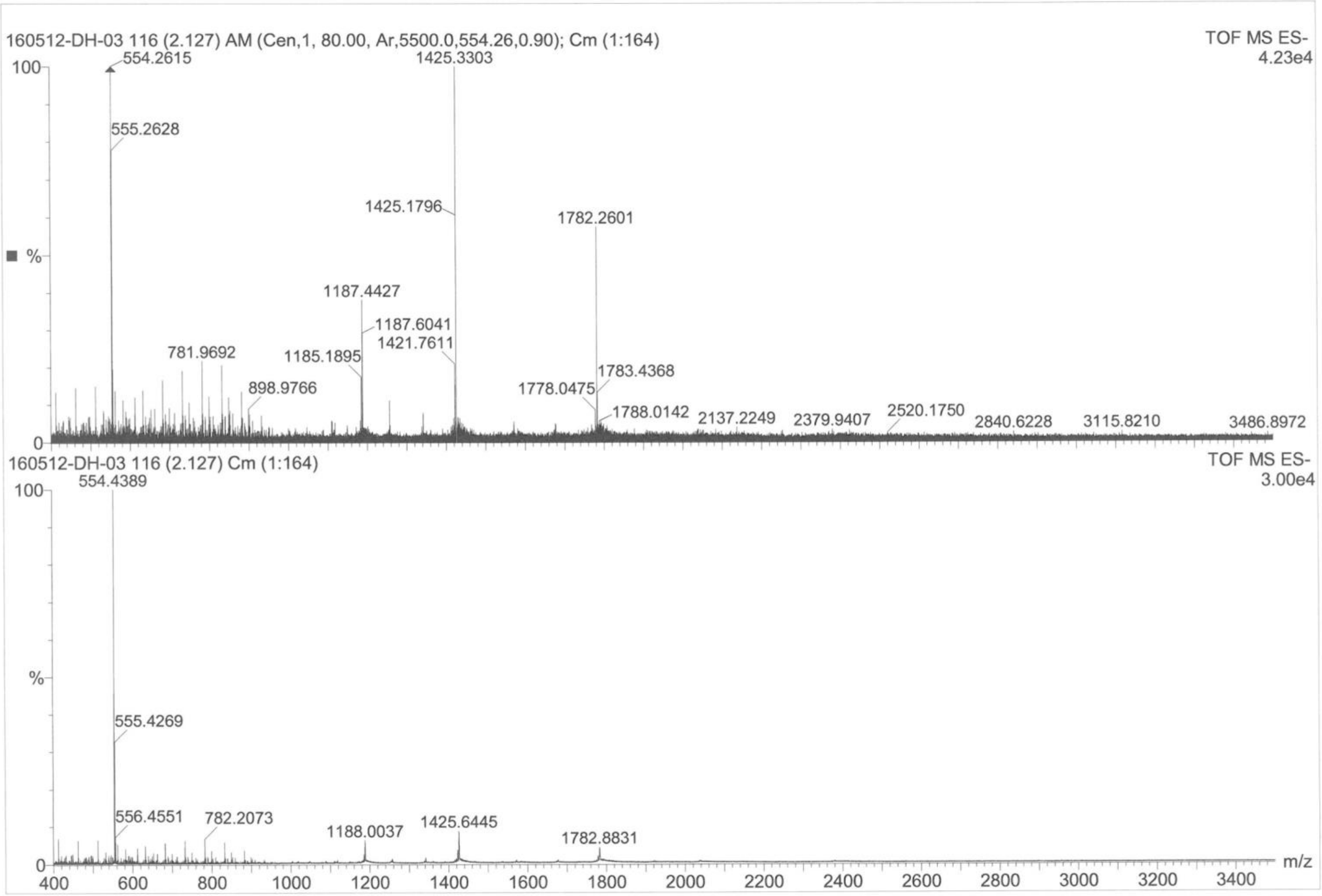

Figure S61. ESI-TOF mass spectrum of **ON13** (upper panel, leucine encephalin [M]$^{-}$ 554.2615 used as internal standard).

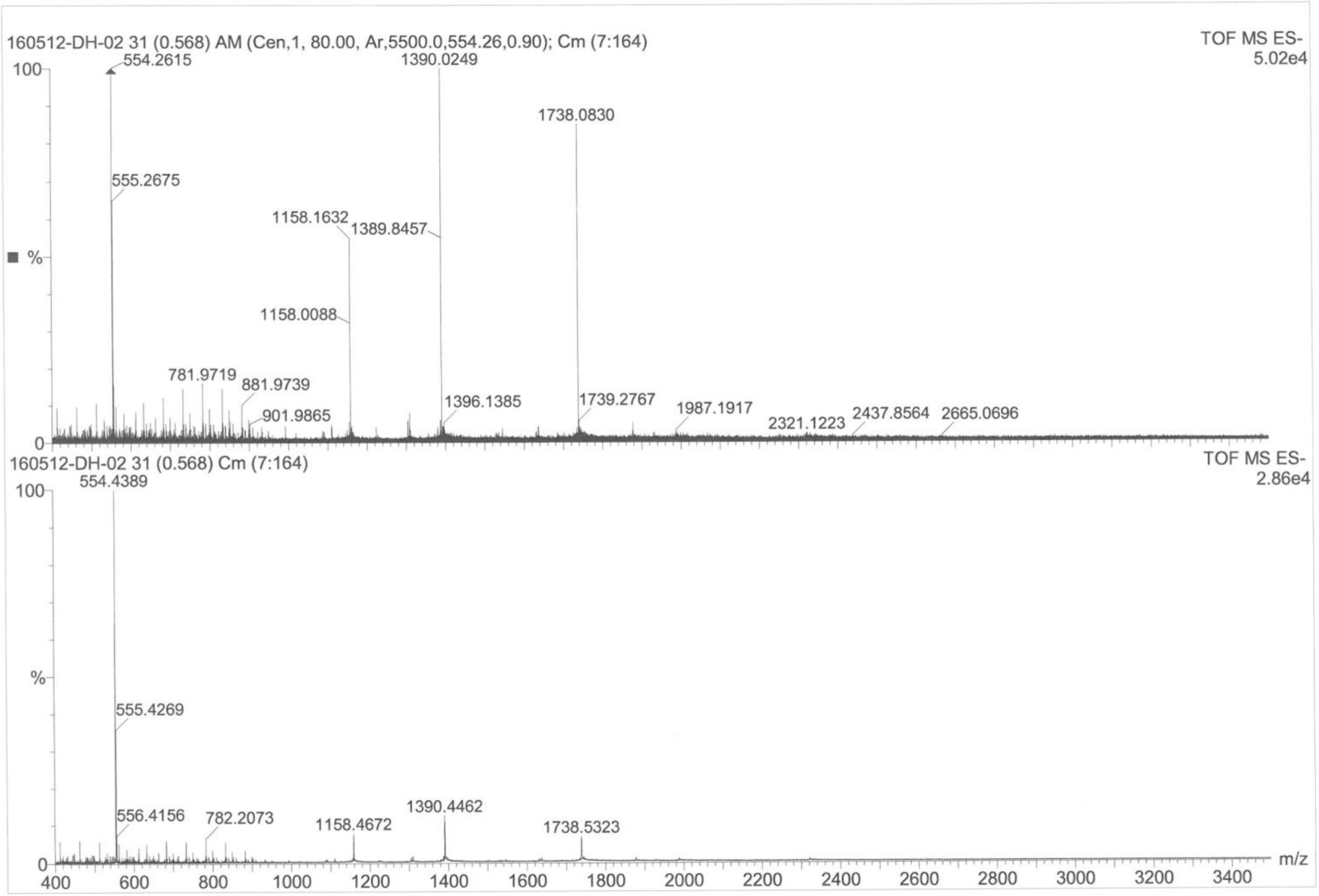

Figure S62. ESI-TOF mass spectrum of **ON14** (upper panel, leucine encephalin $[M]^-$ 554.2615 used as internal standard).

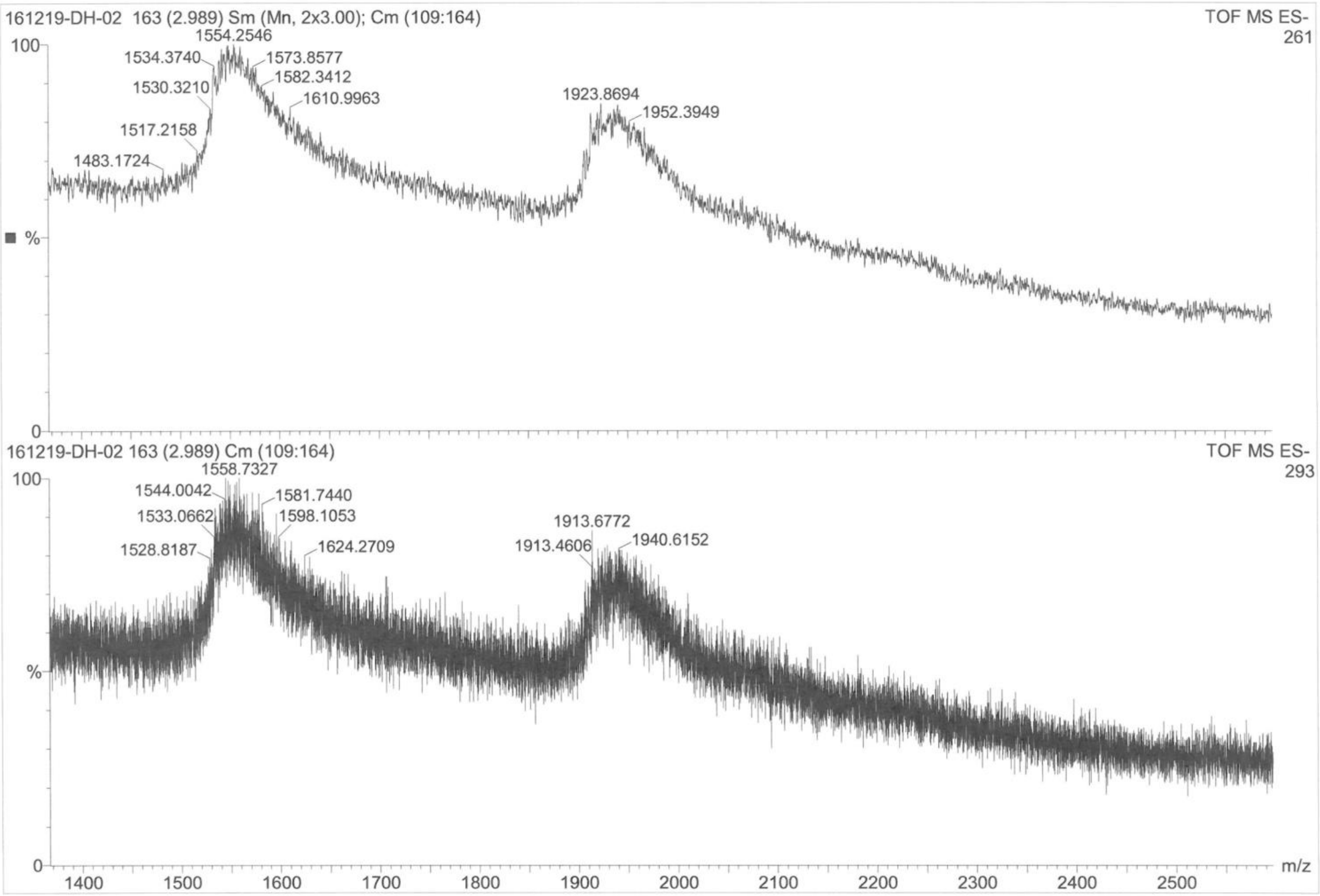

Figure S63. ESI-TOF mass spectrum of **ON15** (upper panel, leucine encephalin $[M]^-$ 554.2615 used as internal standard).

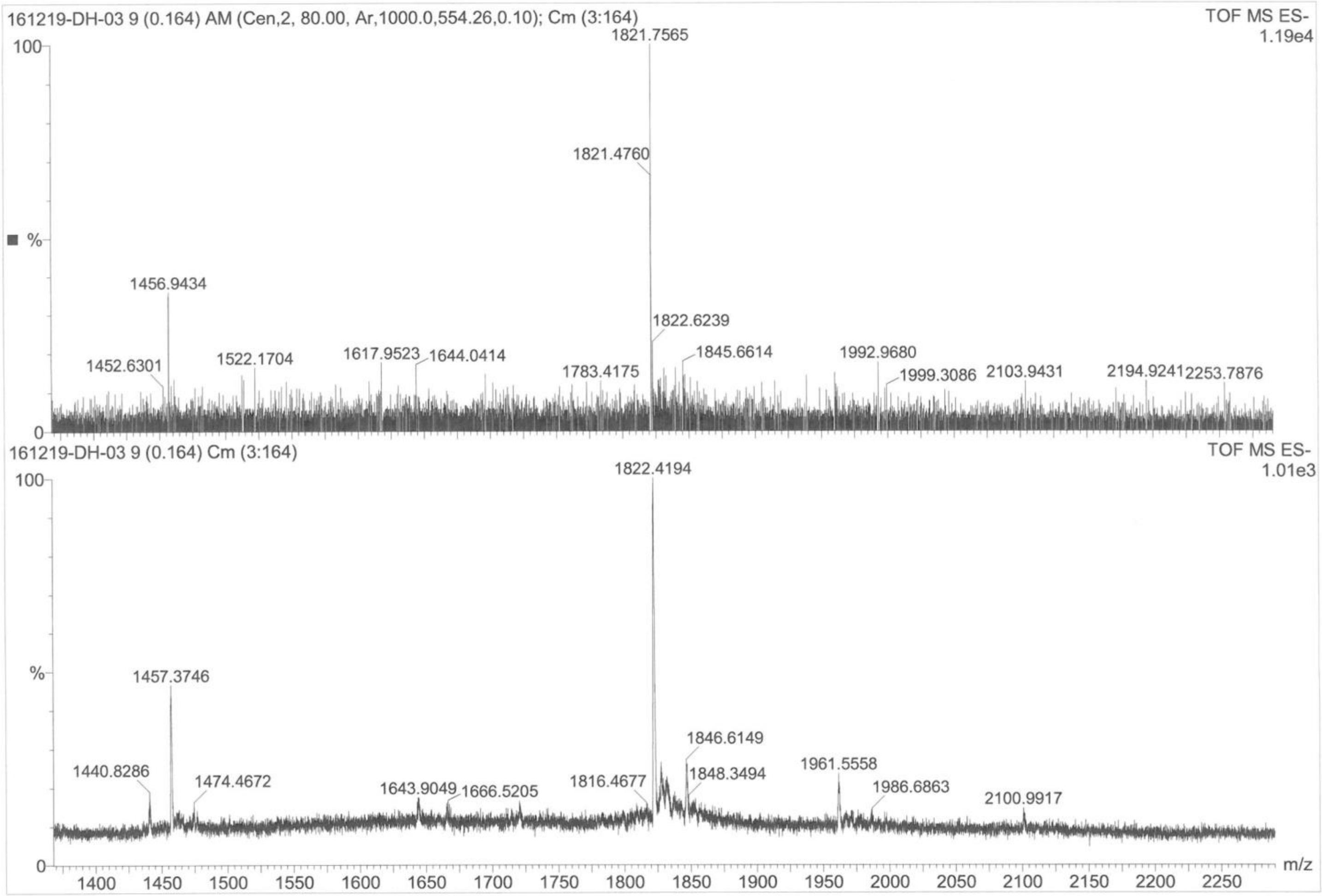

Figure S64. ESI-TOF mass spectrum of **ON17** (upper panel, leucine encephalin $[M]^-$ 554.2615 used as internal standard).

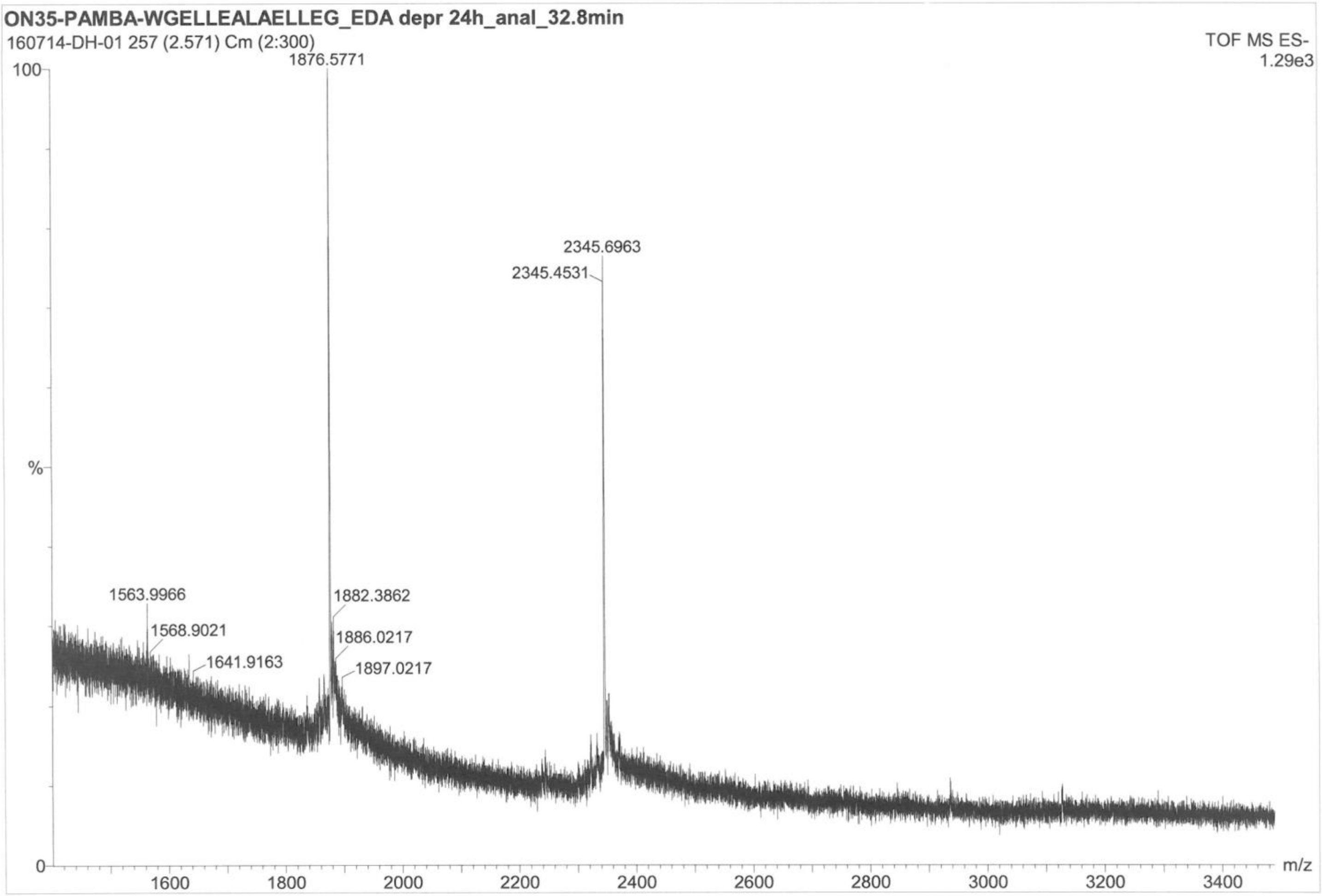

Figure S65. ESI-TOF mass spectrum of **ON18** (leucine encephalin $[M]^-$ 554.2615 used as internal standard).

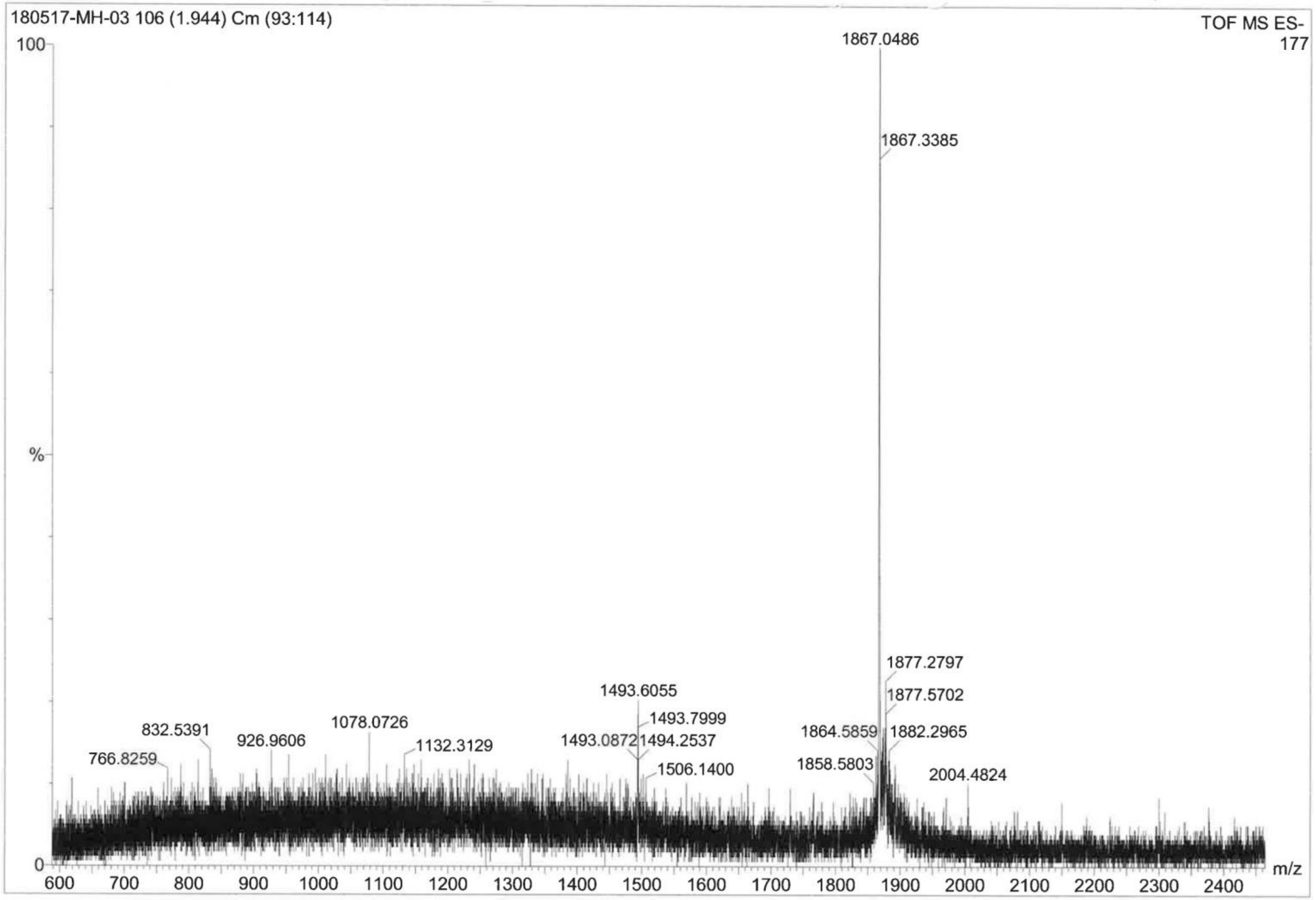

Figure S66. ESI-TOF mass spectrum of **ON19** (leucine encephalin $[M]^-$ 554.2615 used as internal standard).

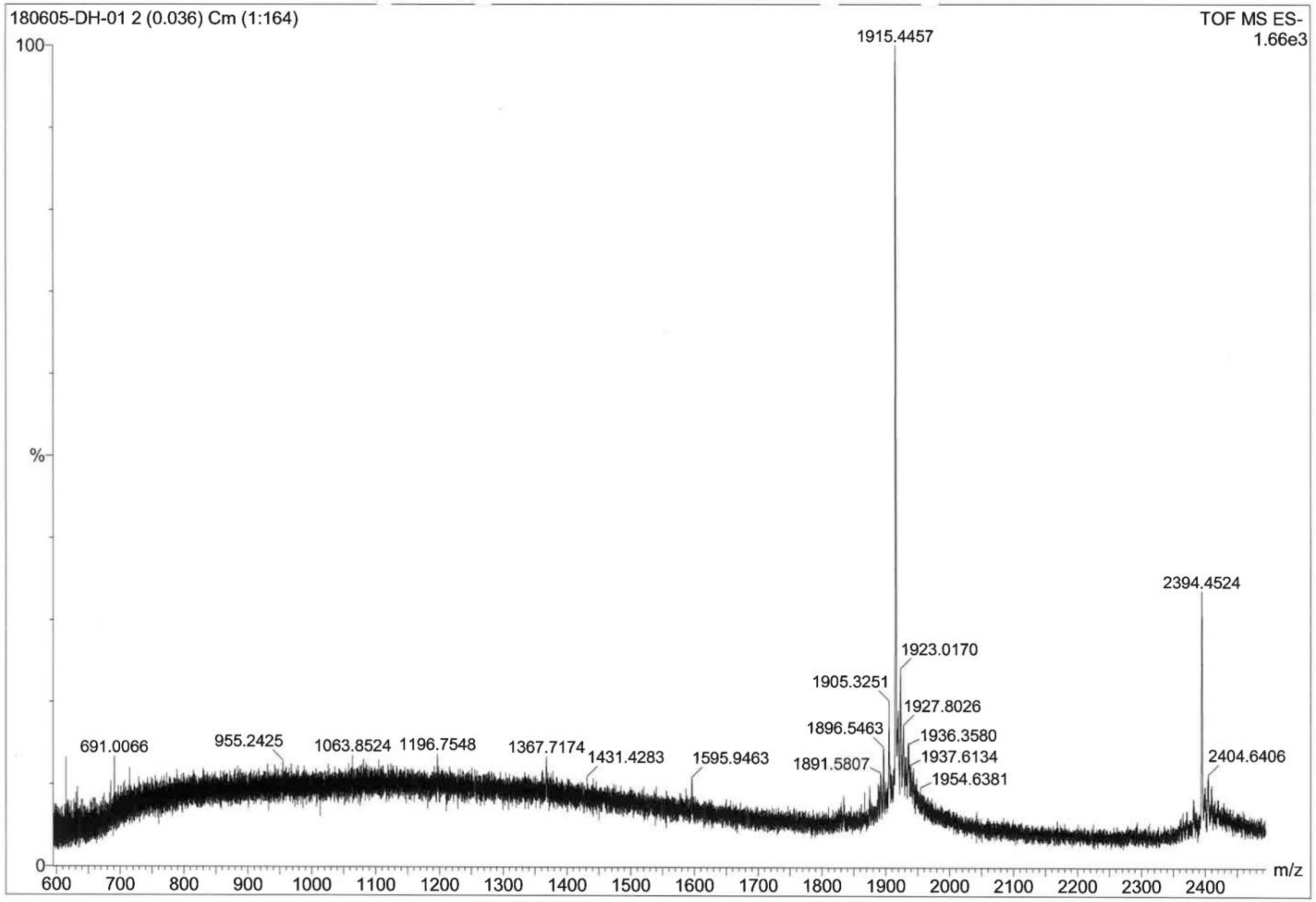

Figure S67. ESI-TOF mass spectrum of **ON20** (leucine encephalin [M]$^-$ 554.2615 used as internal standard).

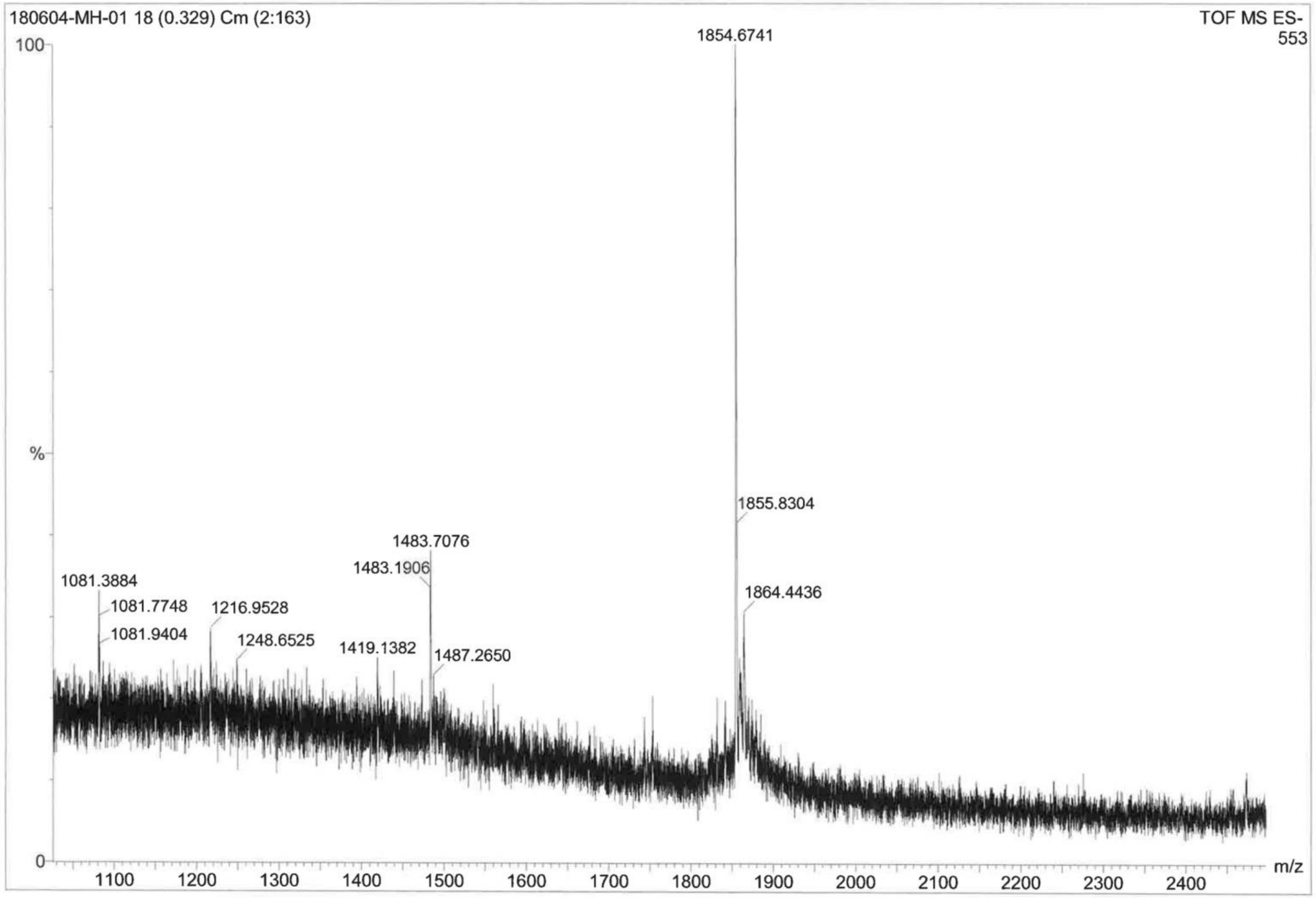

Figure S68. ESI-TOF mass spectrum of **ON22** (leucine encephalin $[M]^-$ 554.2615 used as internal standard).

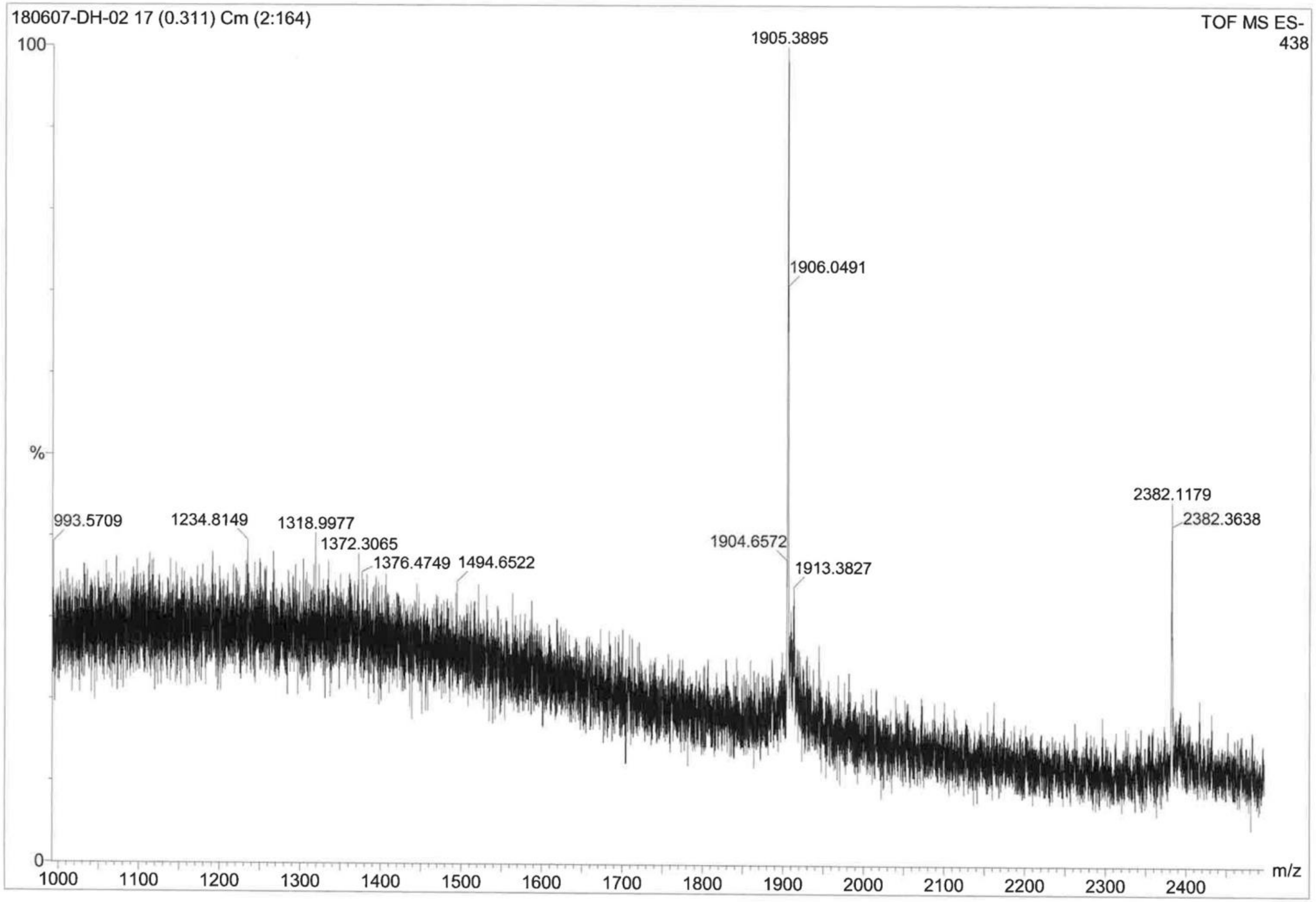

Figure S69. ESI-TOF mass spectrum of **ON23** (leucine encephalin $[M]^{-}$ 554.2615 used as internal standard).

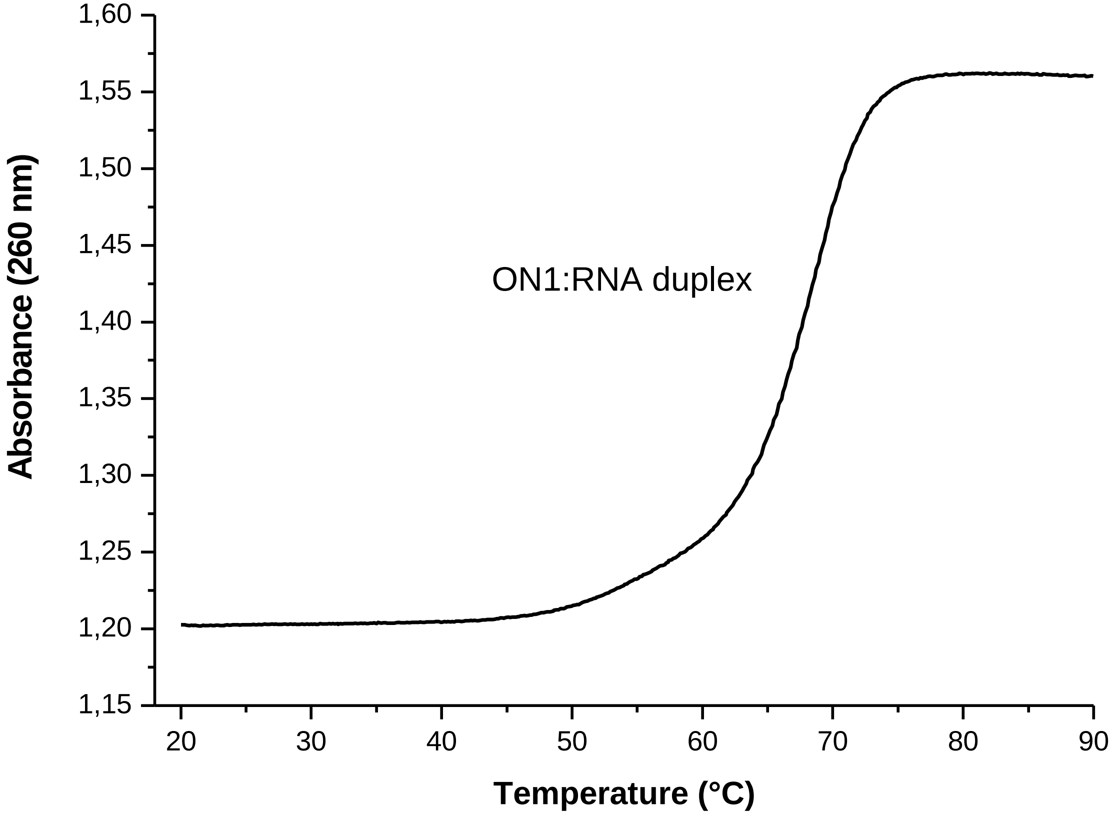

Figure S70. Melting curve of duplex formed by **ON1** with complementary RNA

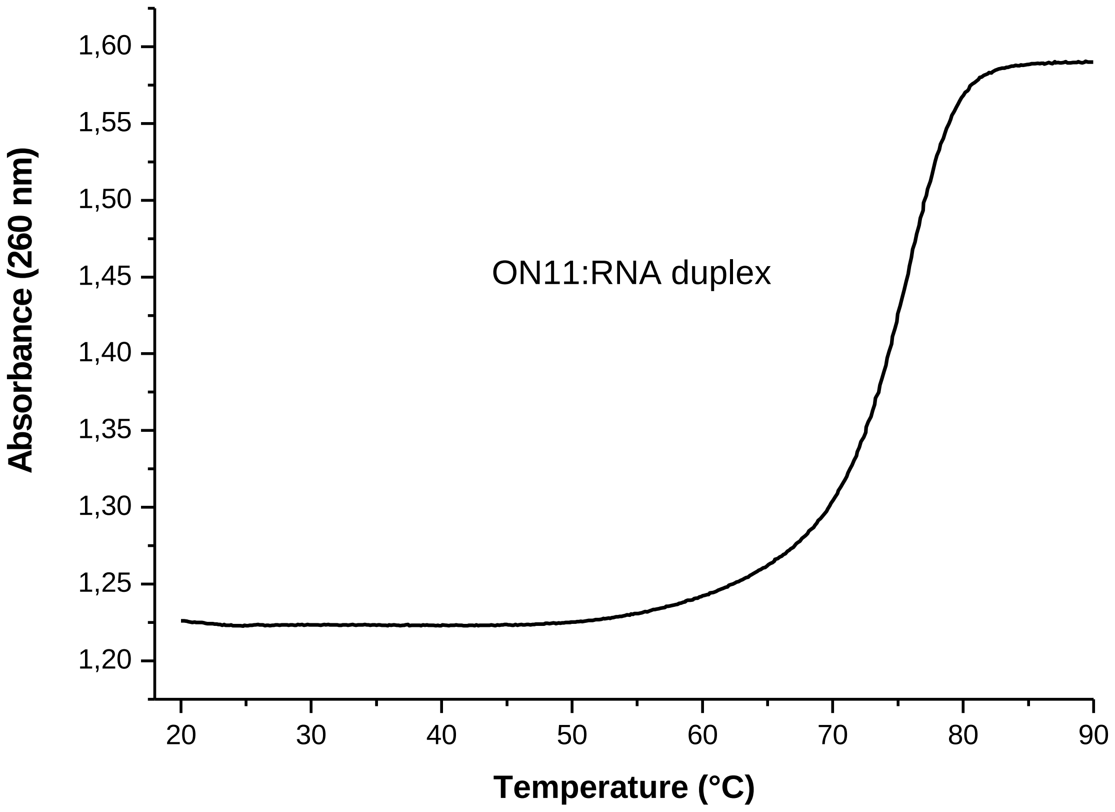

Figure S71. Melting curve of duplex formed by **ON11** with complementary RNA

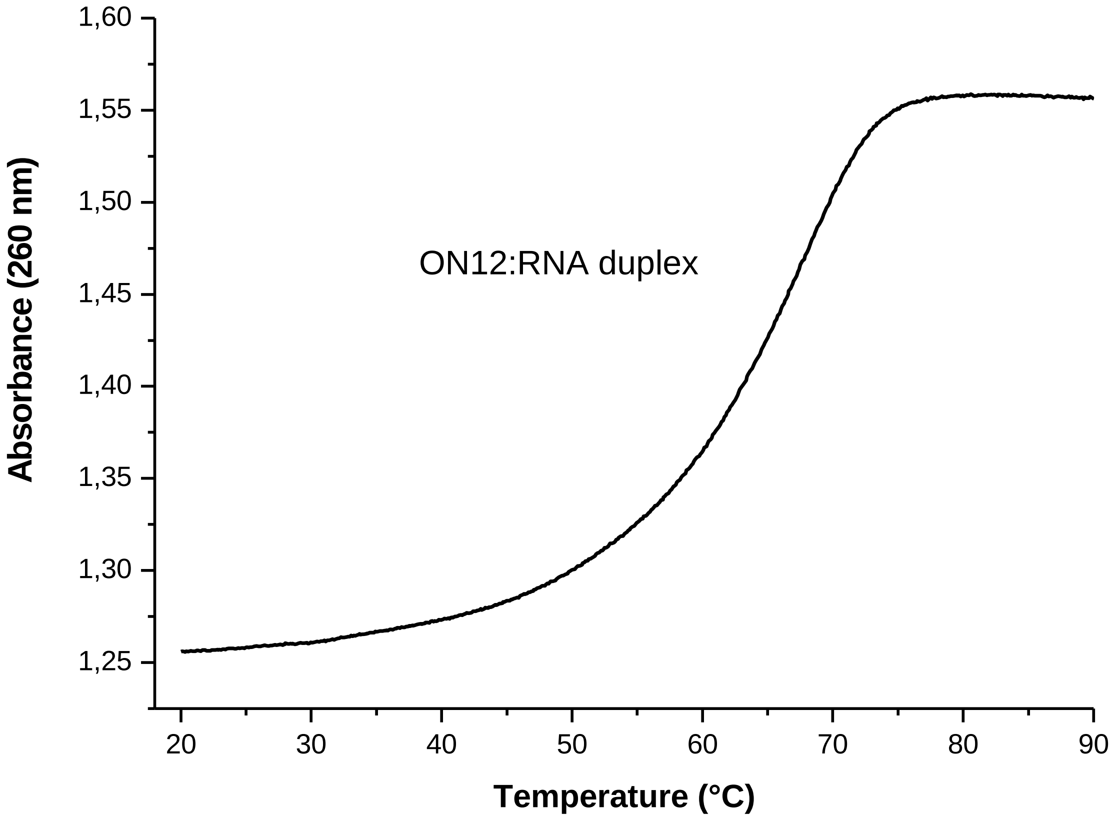

Figure S72. Melting curve of duplex formed by **ON12** with complementary RNA

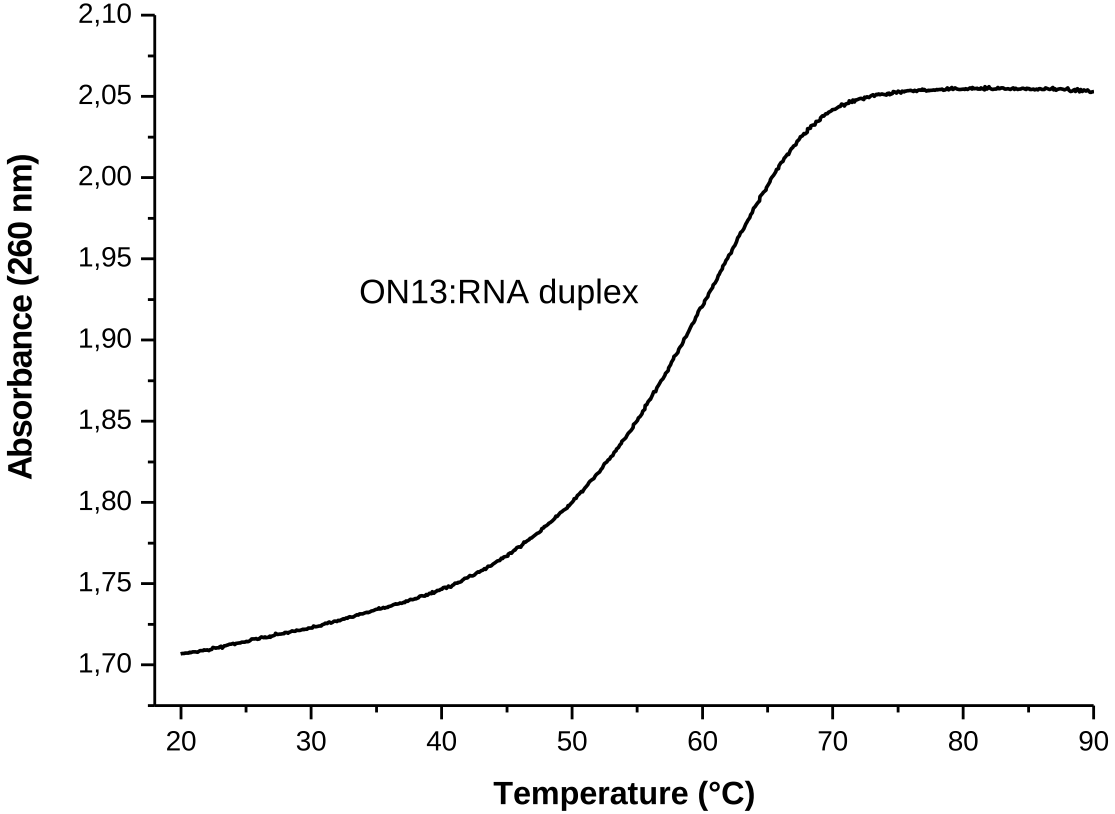

Figure S73. Melting curve of duplex formed by **ON13** with complementary RNA

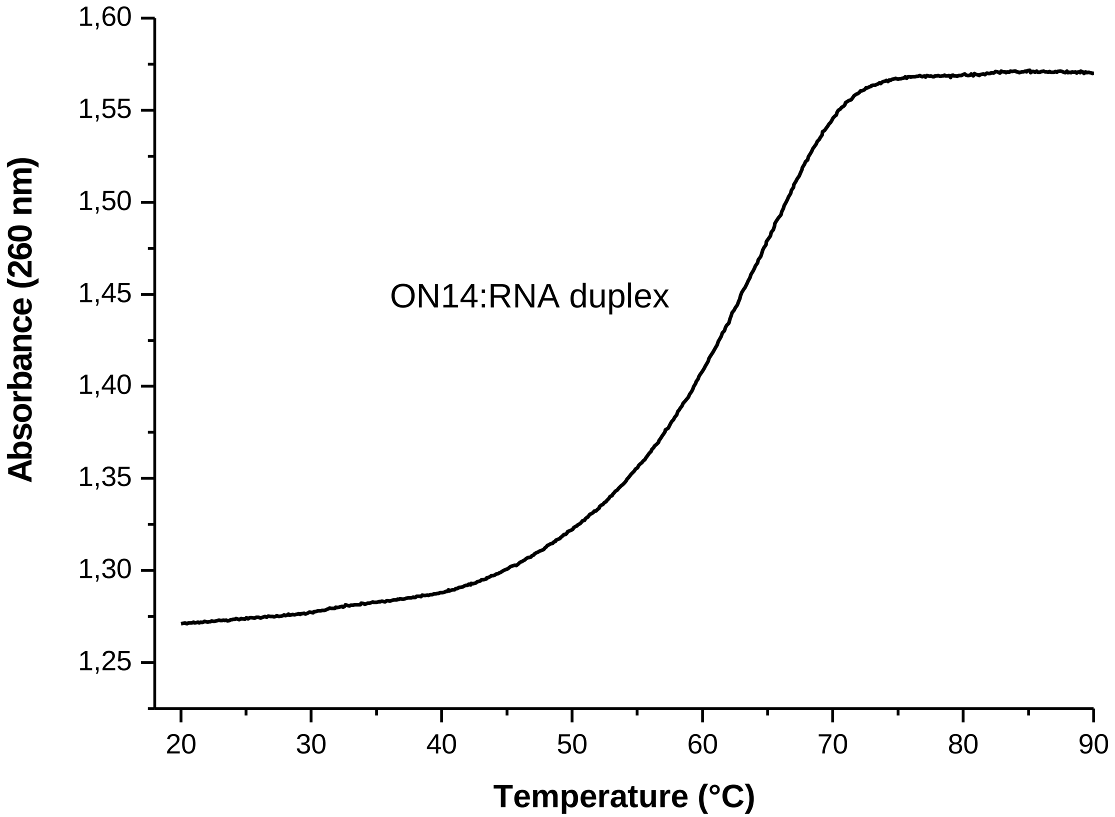

Figure S74. Melting curve of duplex formed by **ON14** with complementary RNA

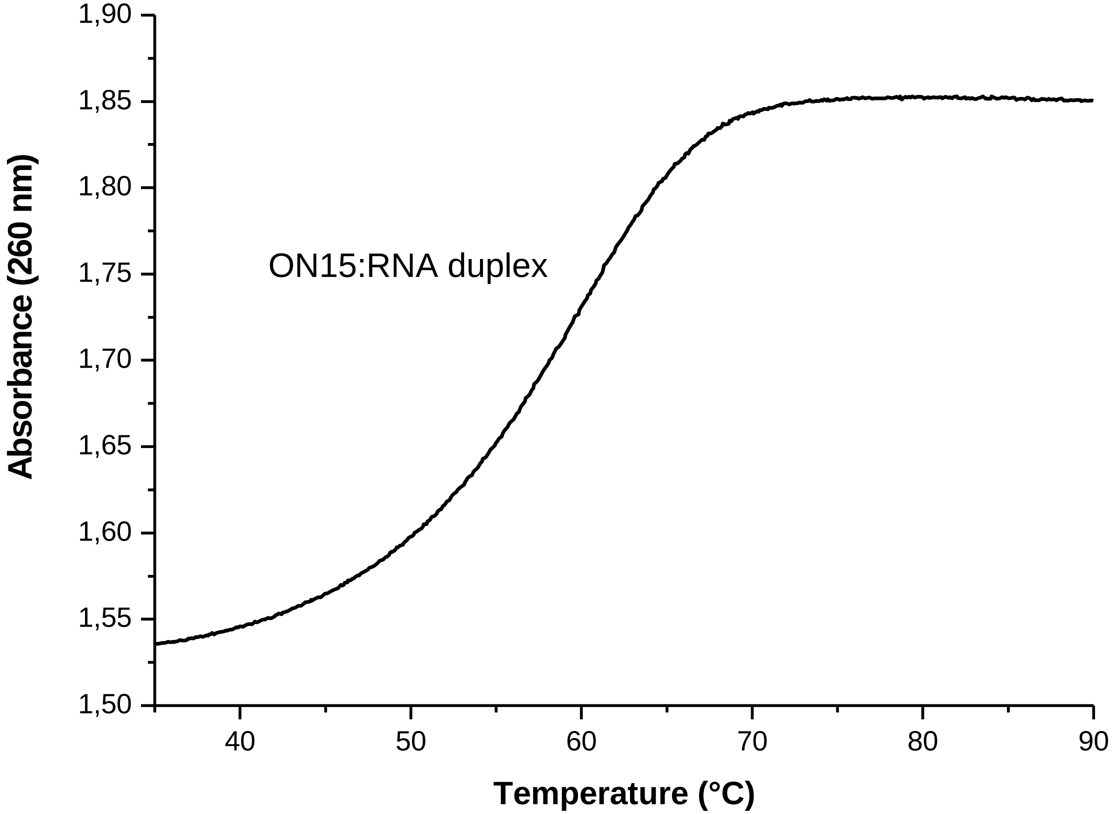

Figure S75. Melting curve of duplex formed by **ON15** with complementary RNA

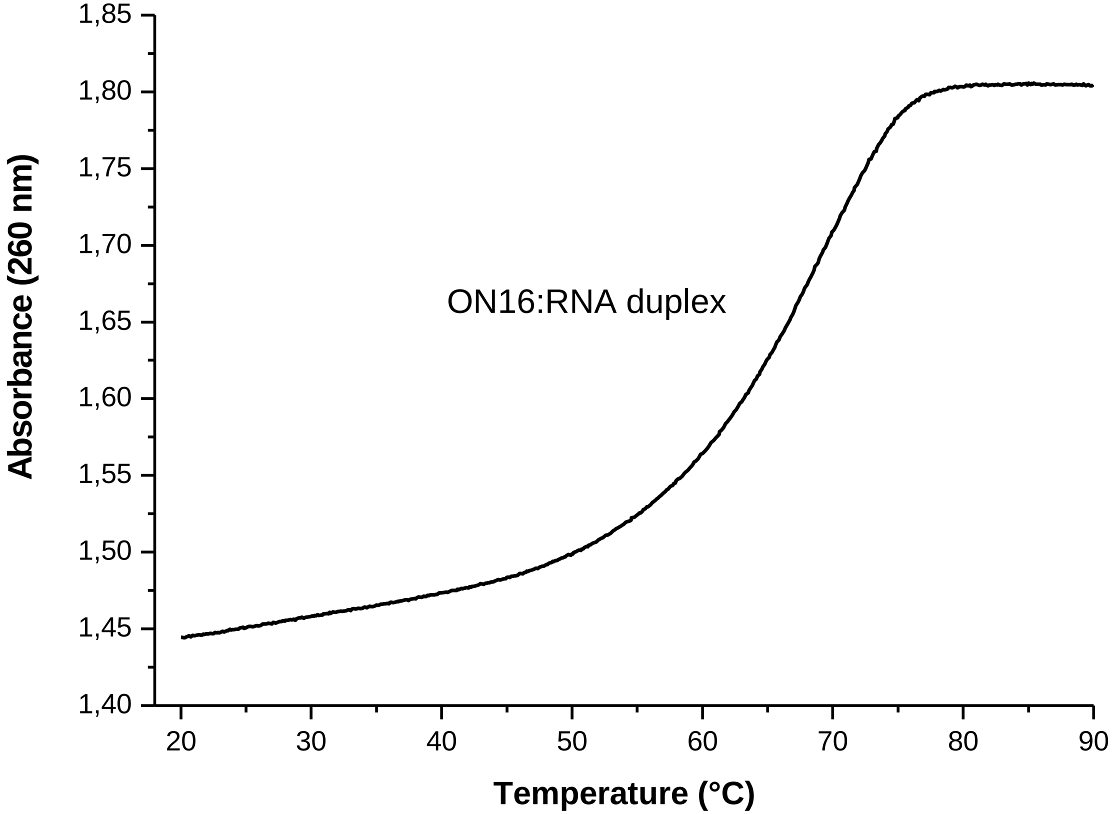

Figure S76. Melting curve of duplex formed by **ON16** with complementary RNA

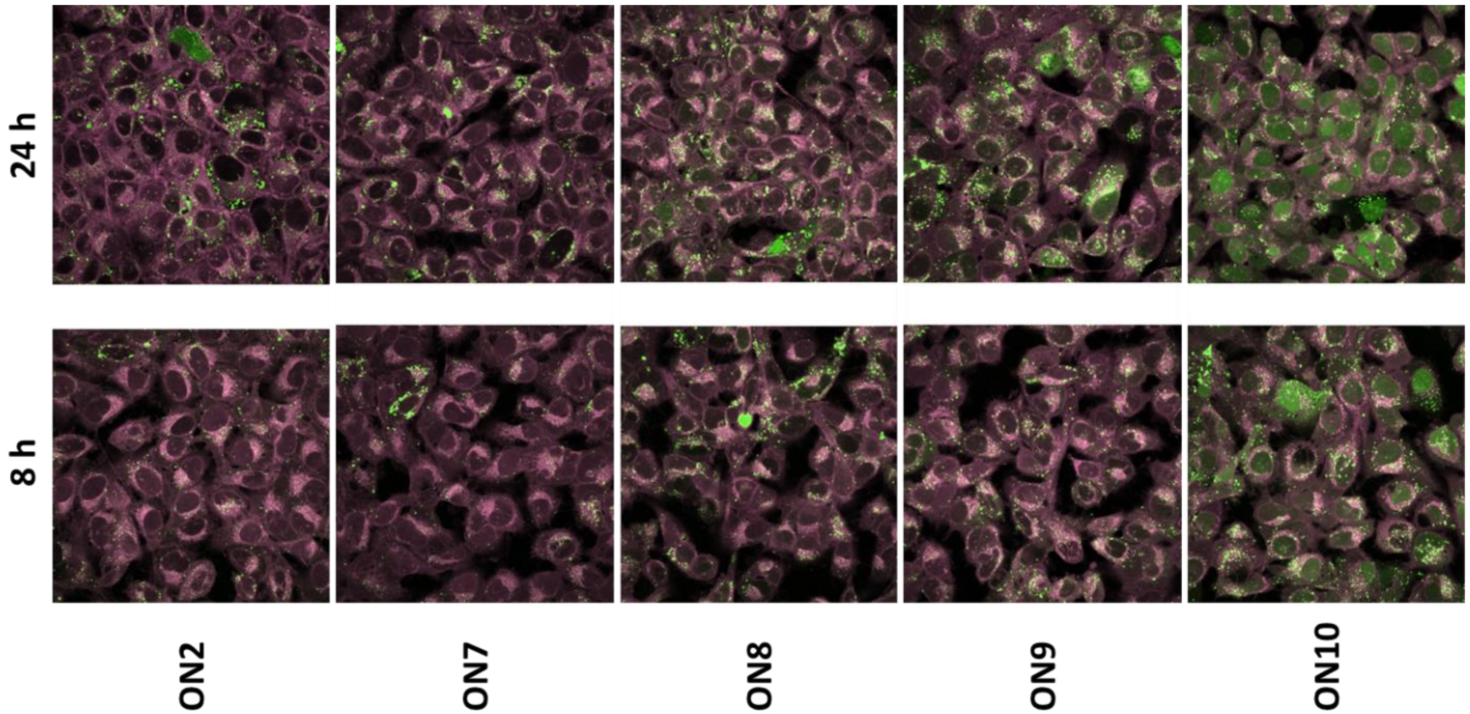

Figure S77. **PS backbone improves endosomal escape and nuclear localization of partial 2′-*O*-AECM modified oligonucleotides**. Confocal laser scanning microscopy images of U-2 OS cells treated with 4 µM of either 18-mer fluorescent-labelled fully 2′-*O*-AECM modified (**ON2**, **CCUCUUACCUCAGUUACA**-6Fam), partially PS 2′-OMe/ 2′-*O*-AECM mixmer (**ON7**, C*C*U*C*U*U***ACCUCAGUUACA**-6Fam), fully PS 2′-*O*-AECM/ 2′-OMe mixmer (**ON8**, **C*C*U*C*U*U***A*C*C*U*C*A***G*U*U*A*C*A***-6Fam), fully PS 2′-*O*-AECM (**ON9**, **C*C*U*C*U*U*A*****C***C***U***C***C***A***G*U*U*A*C*A***-6Fam) and fully PS 2′-OMe (**ON10**, C*C*U*C*U*U*A*C*C*U*C*A*G*U*U*A* C*A*-6Fam) ONs for 8 h and 24 h. Cells were washed before being processed for microscopy and using the CellMask™ Deep Red dye to stain membrane structures. The fluorescein-labelled ONs are visualized in green and membranes in pink. Images represent the central plane of the cell in the *Z*-axis.

Preparation of 2′-*O*-[(*N*-(aminoethyl)carbamoyl)methyl]uridine (2′-*O*-AECM-U monomer). Compound **8** (0.2 g, 0.47 mmol) (Scheme 2) dissolved in of anhydrous methanol (3.5 mL) was treated with ethylenediamine (0.16 mL, 2.37 mmol) at room temperature for 20 h. Volatiles were evaporated *in vacuo* and the crude residue was further treated with 25% aqueous ammonia (8 mL) at ambient temperature for 20 h. Water was partially evaporated under reduced pressure and the residue was freeze-dried. Small amount (25 mg) of resulted crude product was re-dissolved in Milli-Q water (1 mL) and purified using RP HPLC on C18 column (Supelco Discovery® BIO Wide Pore C18-5) with a linear gradient from 0 to 50% of CH_3CN-water (1:1) mixture over 20 min at 25 °C. t_R = 14 min. ES-MS, calcd. *m/z* $[M+H]^+$ 345.14, found 345.14.

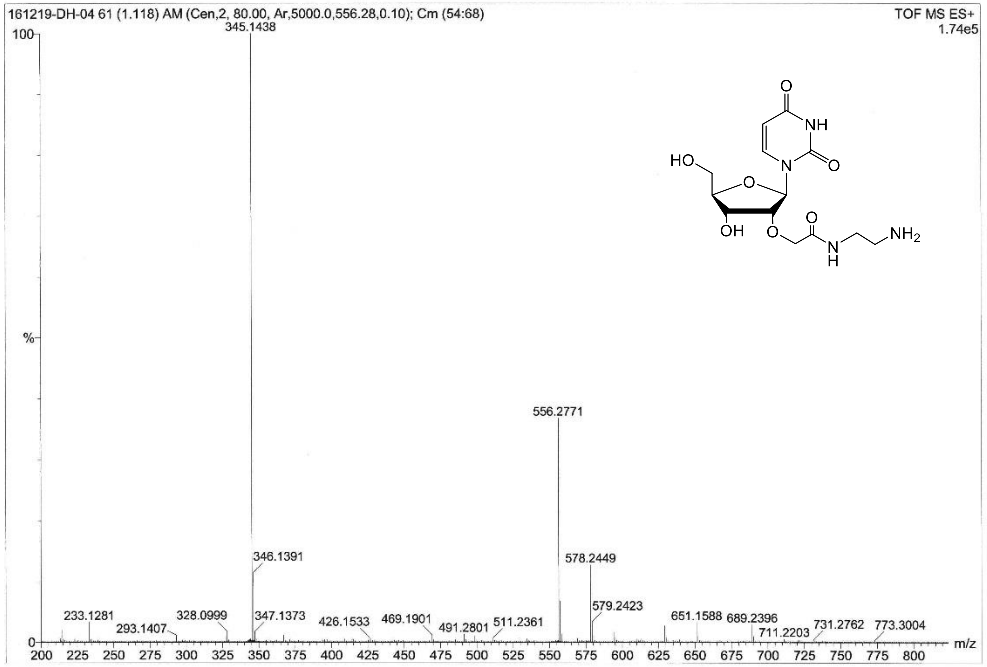

Figure S78. Synthesis procedure and ESI-TOF mass spectrum of 2′-*O*-AECM-U monomer (leucine encephalin $[M]^+$ 556.2771 used as internal standard).

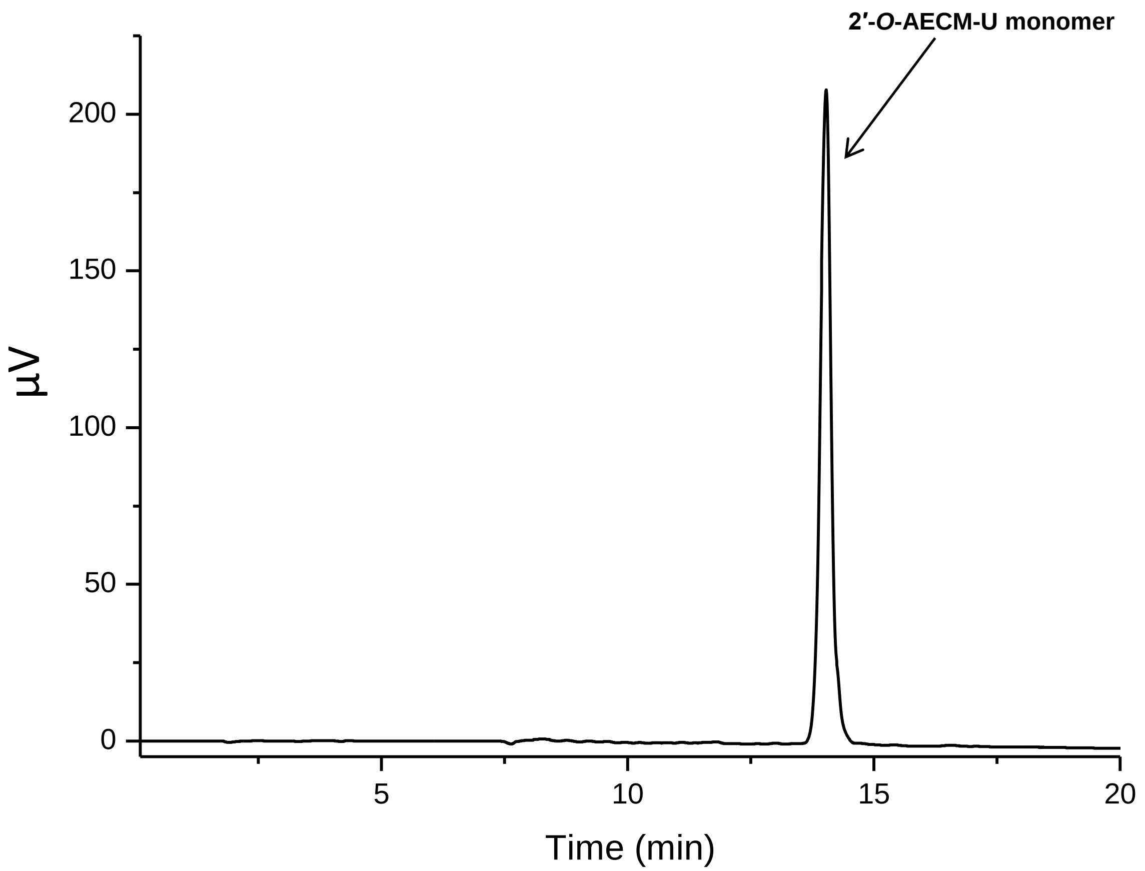

Figure S79. RP-HPLC chromatogram of pure 2′-*O*-AECM-U monomer.

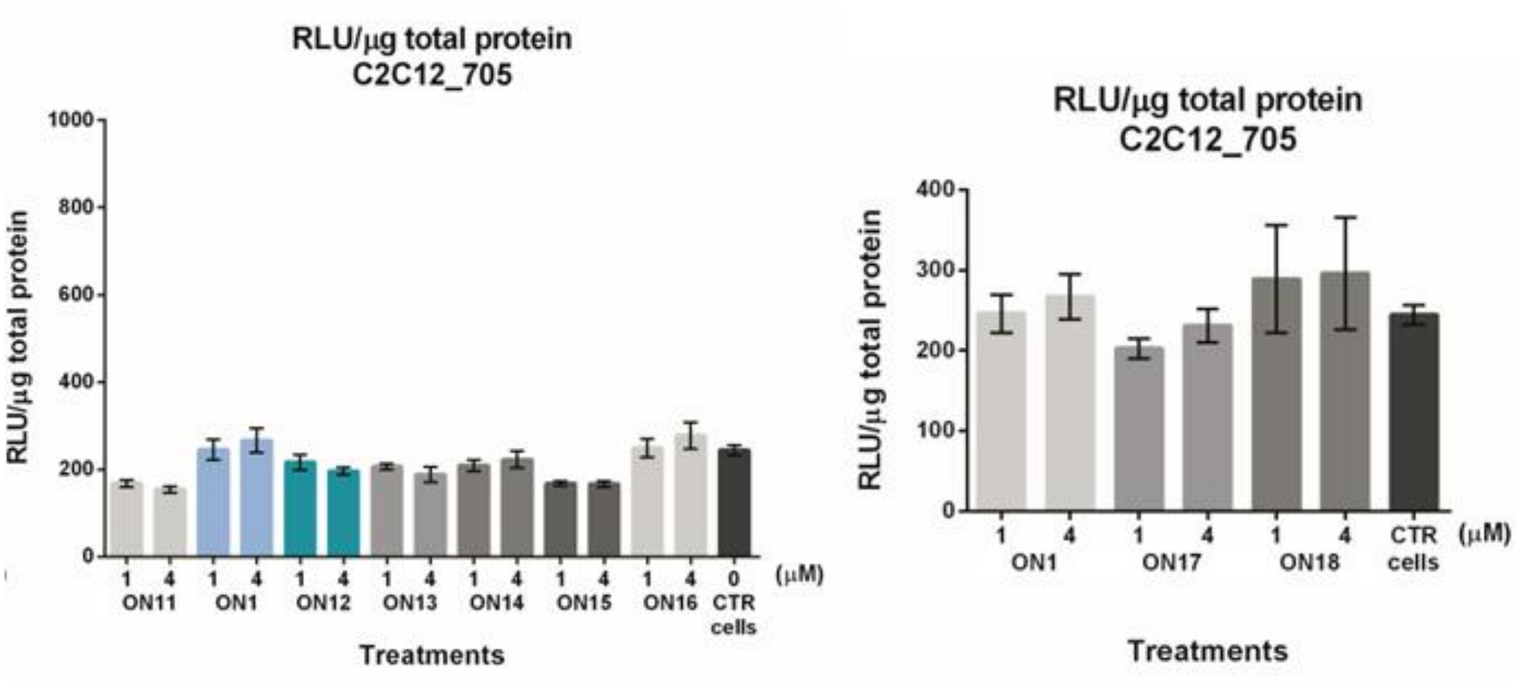

Figure S80. **Luciferase production following splice-correction with ON1 or ONs11-18 by ‘naked’ delivery in Ca^{2+} enriched media in C2C12_705 reporter cell line.** Graphs represent the relative luminescence units (RLU) normalized by micrograms of total protein from C2C12_705 reporter cell line when concentrations of 1 and 4 µM of the 2′-OMe **ON11**, 2′-OMe PS **ON16** or 2′-*O*-AECM **ON1**, **ON12**, **ON13, ON14**, **ON15**, **ON17,** or either ELL-peptide-conjugate **ON18** were delivered ‘naked’ in Ca^{2+} enriched media and the effect measured after 72 h. Data represent the mean ± SEM obtained from four independent experiments.

Splice-correction of a mutated *BTK* intron 4 by 2′-*O*-AECM modified ONs

The 2′-*O*-AECM modified ON (**ON19**) and the corresponding 2′-*O*-AECM modified ON-ELL-peptide conjugate (**ON20**) have the same sequence as the already published 2′-OMe PS ON (**ON21**) proven to give efficient splice-correction [1] (Table 1). Several ONs with different unrelated sequences were used as negative controls: the 2′-OMe PS ONs (**ON16** and **ON24**), the corresponding 2′-*O*-AECM modified control ONs (**ON17** and **ON22**), and the analogous CPO-ELL-peptide conjugate controls (**ON18** and **ON23**) (Table 1). The ONs were delivered to the cells using the CEM method and ON concentrations of 0.25 and 1.0 µM (Figure 5). All the *BTK*-specific ONs were efficiently correcting the splicing as compared to untreated cells as well as to the control ONs (p<0.0001). Differences between **ON19**, **ON20** and **ON21** were not statistically significant, but it is clear that the AECM modified phosphodiester linked ON essentially is on par with the 2′-OMe PS ON despite the absence of PS linkages. Importantly, the result correlates well with the outcome of splice-correction in the U-2 OS_705 cell line. Collectively, this shows efficient correction of two reporters with different readouts using ONs devoid of PS modification.

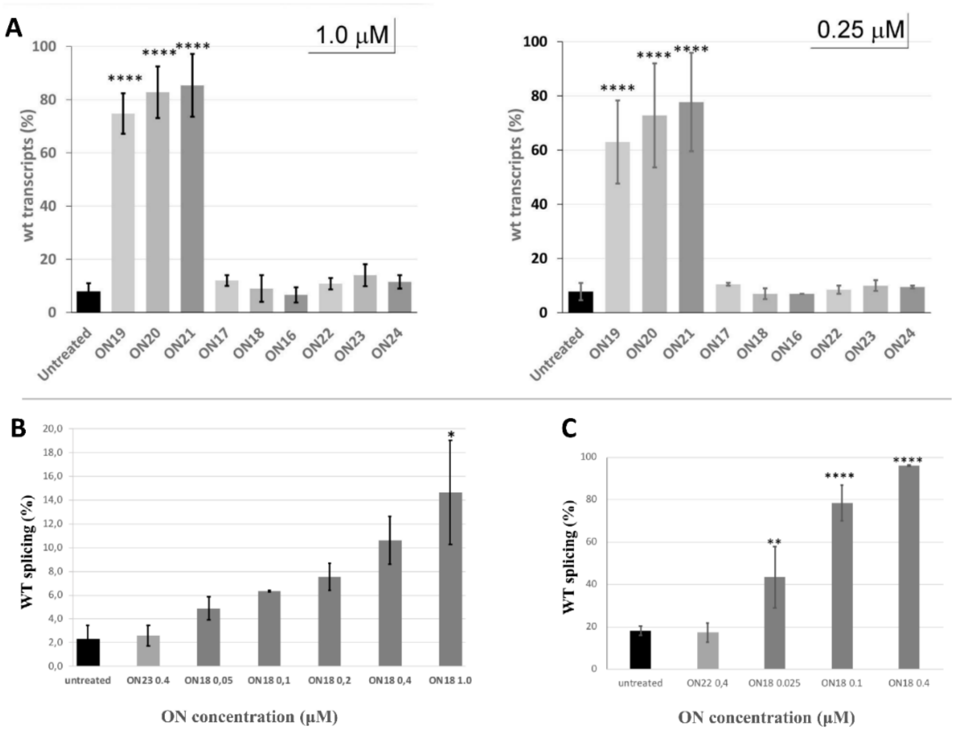

Figure S81. (A, upper panel) Extent of splice-correction of mutated *BTK* intron 4 in stably transfected U-2 OS cells. Graphs represent percentage of splice corrected transcripts as determined by RT-PCR. RNA was extracted from cells after 3 days gymnosis with ONs in Ca^{2+} enriched media at 1.0 µM (left panel) and 0.25 µM (right panel), and RT-PCR performed and analyzed on 1.2% agarose gels (examples of gels are shown in Supplementary Figure S81). The fragments were quantified and the percentage of wt RNA calculated using the QuantityOne software. *HPRT* was used as RNA quality control. UT = untreated cells, NC = negative PCR control, **ON19** = 2′-*O*-AECM ON, **ON20** = 2′-*O*-AECM ELL-ON, **ON21** = 2′-OMe PS ON. Controls with irrelevant sequences: **ON16** and **ON24** = 2′-OMe PS ON controls, **ON17** and **ON22** = 2′-*O*-AECM controls, and **ON18** and **ON23** = CPO-ELL-peptide controls. The graphs show the mean of 5 independent experiments performed in duplicates. Data represent the mean ± SEM (*BTK* ONs n=5) while the different negative controls vary, some being present in all, and some in selected experiments n=2-5. **** p<0.0001 against irrelevant controls. Statistics determined using Oneway ANOVA. Lower panel shows the splice-correction at different concentrations by the 2′-*O*-AECM ELL-peptide-conjugate **ON18** in (B) HeLa705 cells and (C) U2OS-705 cells as determined by RT-PCR (examples of gels are shown in Supplementary Figure S83). ONs at indicated concentrations were added in DMEM medium supplemented with 10% fetal bovine serum and 9 mM extra $CaCl_2$ and incubated for 72 h. Data shows the mean of at least two independent experiments, error bars indicate SEM. ON22 and ON23 are irrelevant control ONs. Statistical analysis was made using one-way ANOVA and ON-treatments compared to untreated cells * p<0.05, **p<0.01, **** p<0.0001.

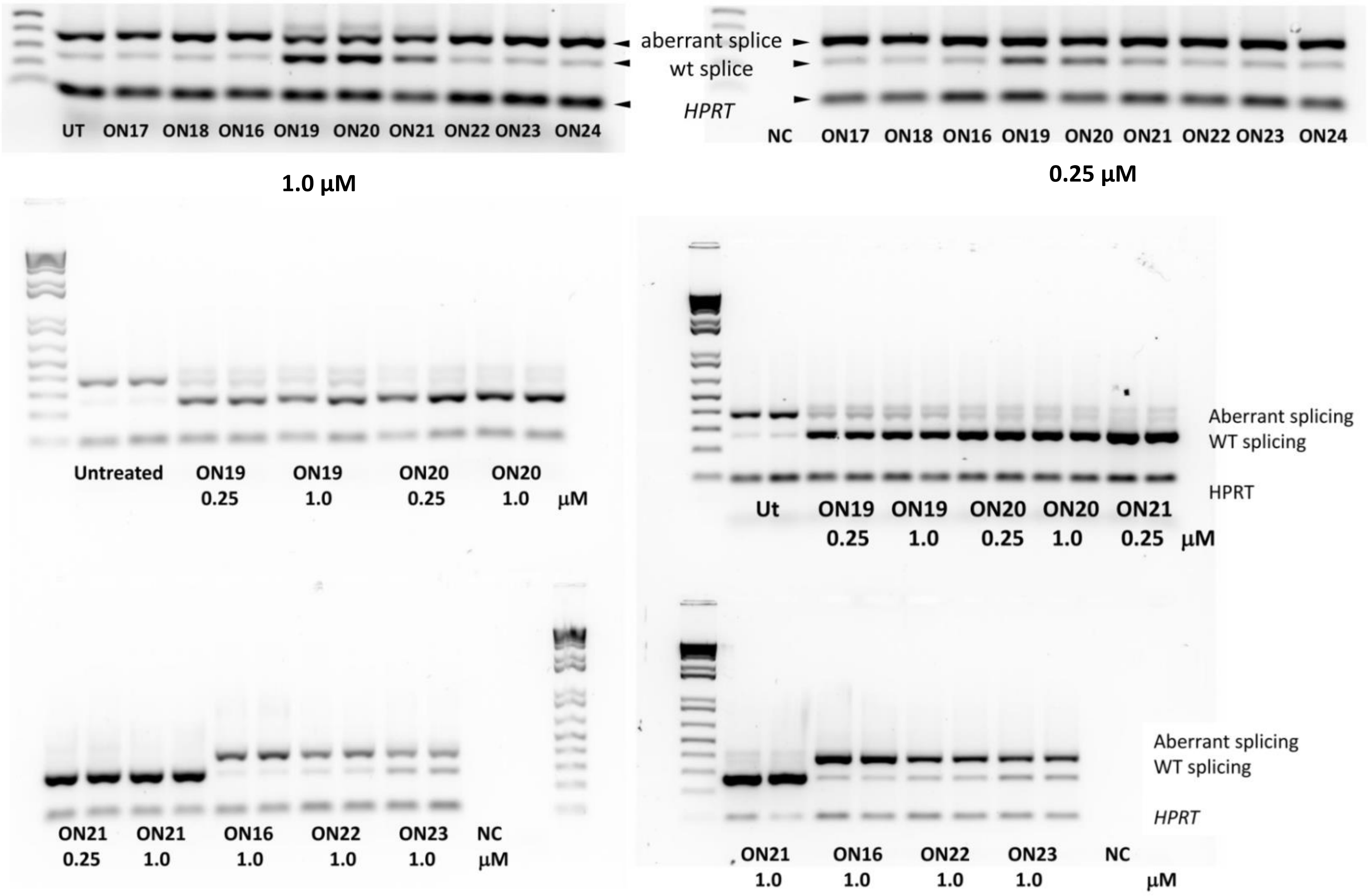

Figure S82. **Representative gels providing examples of data from splice-correction of mutated BTK intron 4 as determined by RT-PCR**. RNA was extracted from cells after 3 days gymnosis with ONs (Table 1) at 1.0 µM and 0.25 µM. The RT-PCR performed and analyzed on 1.2% agarose gels in 0.5xTBE buffer. ONs at 1.0 µM and 0.25 µM as indicated. Duplicate samples from gymnosis at indicated concentrations. UT = untreated cells, NC = negative PCR control.

1. Bestas B, PM Moreno, KE Blomberg, DK Mohammad, AF Saleh, T Sutlu, JZ Nordin, P Guterstam, MO Gustafsson, S Kharazi, et al. (2014). Splice-correcting oligonucleotides restore BTK function in X-linked agammaglobulinemia model. J Clin Invest 124:4067–4081.

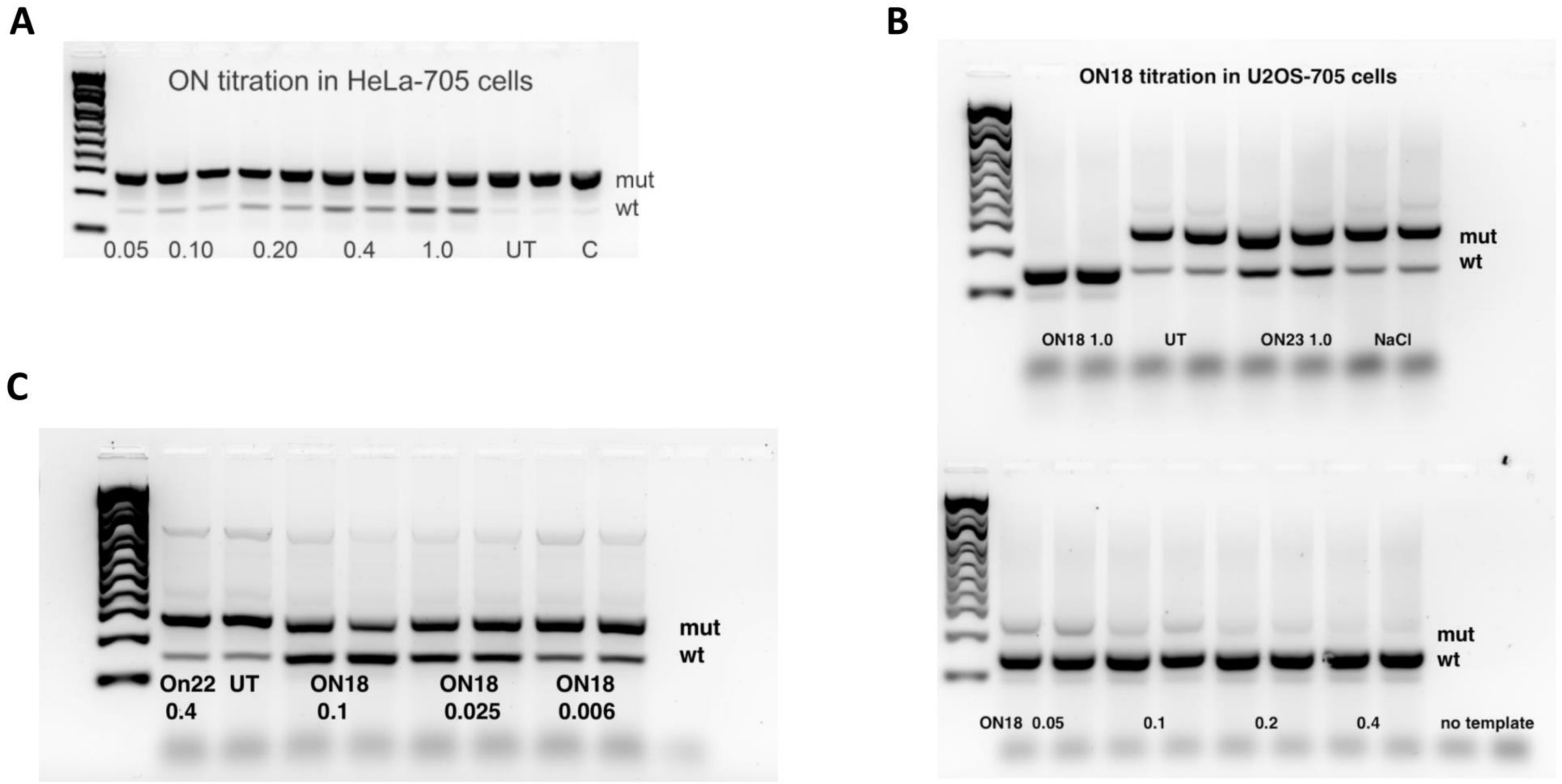

Figure S83. **Representative gels providing examples of data from splice-correction of mutated β-globin intron as determined by RT-PCR**. RNA was extracted from cells after 3 days gymnosis with ONs at indicated concentrations (μM). RT-PCRs were performed and analyzed on 1.2% agarose gels in 0.5xTBE buffer. ON 18 titration on (A) Hela705-cells, (B) U2OS-705 cells at 0.5 – 1.0 μM, and (C) U2OS-705 cells at 0.1-0.006 μM. Duplicate samples at indicated concentrations. UT = untreated cells, NaCl = 0.9% NaCl added in same volume as added ONs.

Cytotoxicity evaluation of 2′-*O*-AECM modified oligonucleotides and monomers.

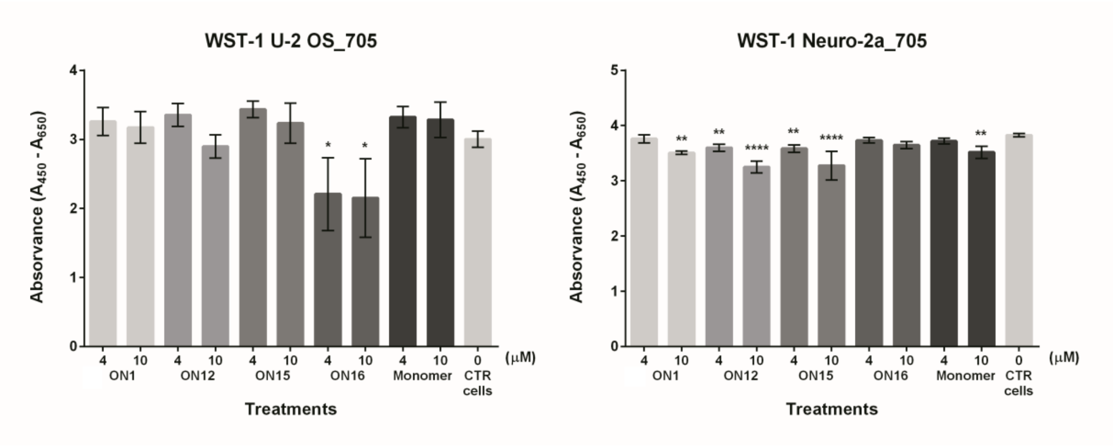

Figure S84. **WST-1 cytotoxicity evaluation of different 2′-*O*-AECM containing ONs and 2′-*O*-AECM-U**. Graphs represent the absorbance values (from the difference between 450 and 650 nm of the samples against a background control) for U-2 OS_705 and Neuro-2a_705 reporter cell lines untreated and with calcium plus 4 or 10 µM ON (or 2′-*O*-AECM-U monomer) for 72 h. Data represents the mean ± SEM obtained from two independent experiments.

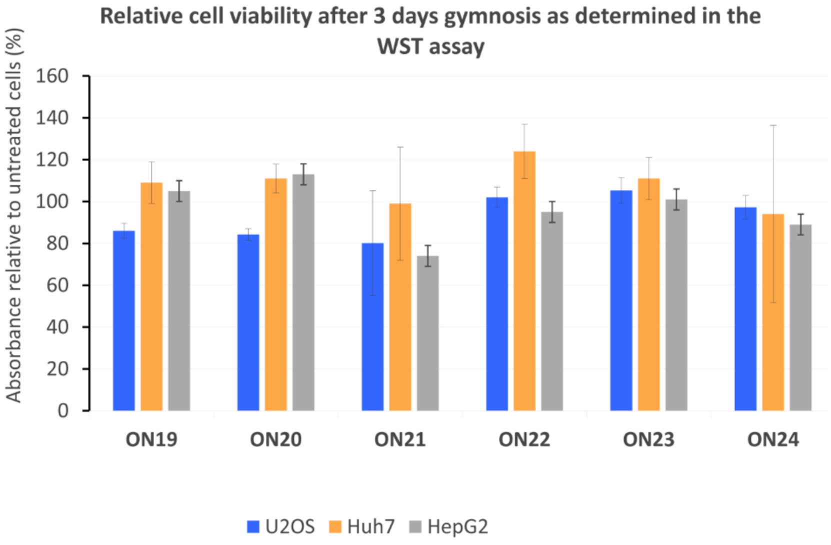

Figure S85. **Additional toxicity analysis (WST-1) in three different cell lines with ONs19-24.** Cells were seeded at a density of 7x10^3 cells per well in a 96-well plate the day before ON treatment using 1.0 µM of the respective ON as described for the splice-switching assay. After 72 h, medium was removed and new medium supplemented with WST-1 reagent (final dilution 1:10) was added to the wells. The cells were incubated for 2 h at 37 °C, 5% CO2 in 95% humidity, followed by absorbance measurements at 450 and 650 nm, for formazan product absorbance and reference wavelength, respectively. Values are presented relative to untreated cells. All analyses were performed in triplicate. No statistically significant differences were found between the tested ONs using Oneway Annova with multiple comparisons. N=3.

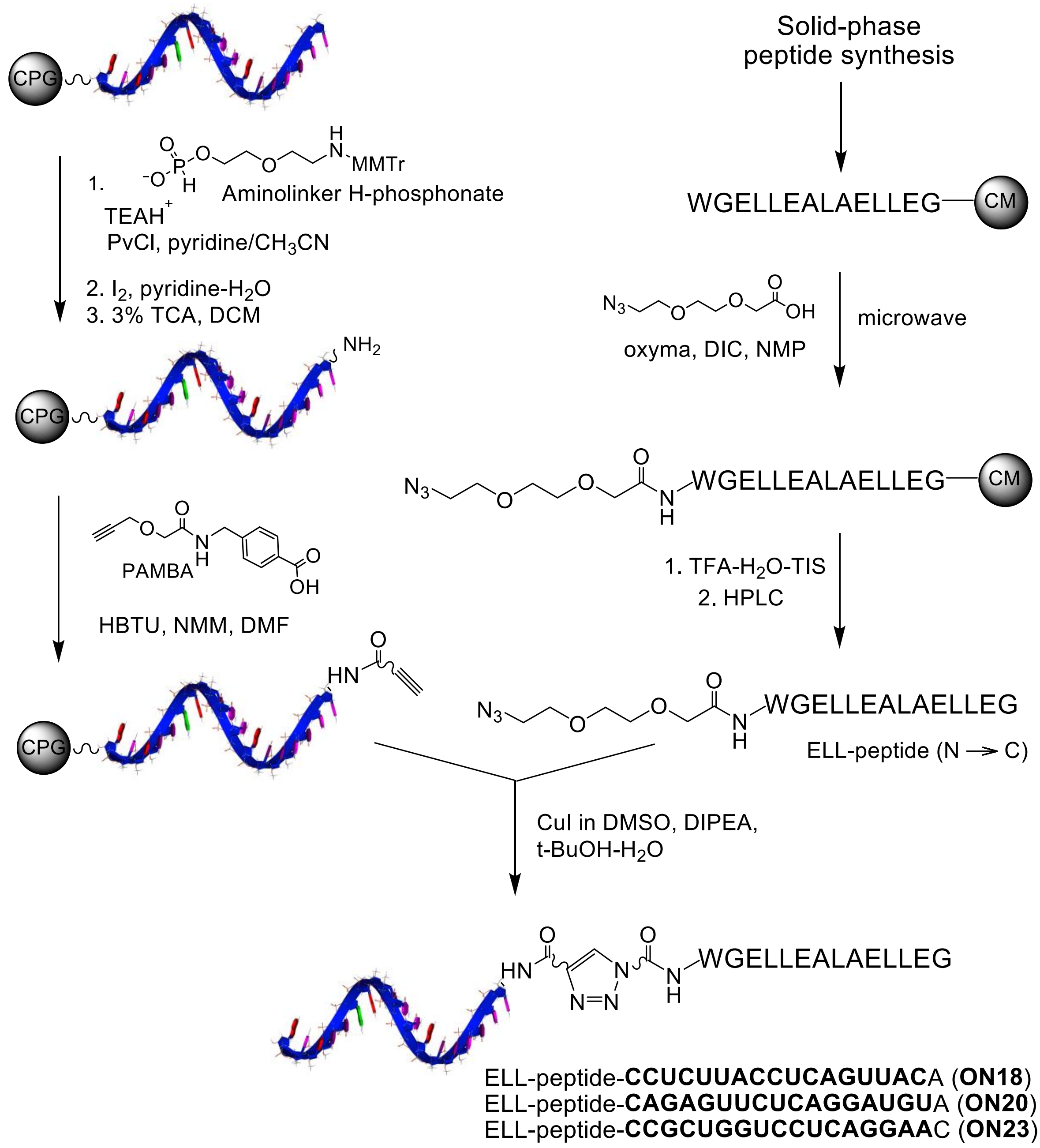

Scheme S1. **Schematic representation of the synthesis of the 2′-*O*-AECM modified oligonucleotide–ELL-peptide conjugates (ON18, ON20 and ON23).** Abbreviations: MMTr, 4-methoxytrityl; TEA, triethylamine; Pv, pivaloyl; TCA, trichloroacetic acid; DCM, dichloromethane; PAMBA, 4-((2-(prop-2-yn-1-yloxy)acetamido)methyl) benzoic acid; HBTU, 2-(1*H*-benzotriazole-1-yl)-1,1,3,3-tetramethyluronium hexafluorophosphate; NMM, 4-methylmorpholine; DMF, *N*,*N*-dimethylformamide; oxyma, ethyl (hydroxyimino)cyanoacetate; DIC, *N,N′*-diisopropylcarbodiimide; NMP, 1-methyl-2-pyrrolidinone; TFA, trifluoroacetic acid; TIS, triisopropylsilane; DMSO, dimethyl sulfoxide; DIPEA, *N*,*N*-diisopropylethylamine; t-Bu, tert-butyl; CM, Rink Amide ChemMatrix® resin.
